# The evolution of the global disease burden of polycystic ovary syndrome and the role of regional heterogeneity in high body mass index exposure: a spatiotemporal analysis based on the global burden of disease 2021

**DOI:** 10.3389/frph.2025.1600995

**Published:** 2025-11-04

**Authors:** Ying Feng, Lei Wu, Jianrong Liu, Yongliang Feng

**Affiliations:** ^1^ The Reproductive Medicine Department of Shanxi Provincial People’s Hospital, Shanxi Medical University, Taiyuan, China; ^2^ The Oncology Department of Shanxi Provincial People’s Hospital, Shanxi Medical University, Taiyuan, China; ^3^ School of Public Health, Shanxi Medical University, Taiyuan, China

**Keywords:** polycystic ovary syndrome, body mass index, disease burden, global health, non-communicable diseases, menopause

## Abstract

**Introduction:**

This study utilized data from the Global Burden of Disease (GBD) 2021 study to conduct a comprehensive analysis of the global burden of polycystic ovary syndrome (PCOS) from 1990 to 2021.

**Methods:**

We reported the trends of prevalence, incidence, years lived with disability (YLDs), and disability-adjusted life years (DALYs) in 204 countries and regions, stratified by age and sociodemographic index (SDI). All estimates were presented as absolute numbers and age-standardized rates (ASR) per 100,000 population, with 95% uncertainty intervals [95% UI] and estimated annual percentage changes (EAPCs) with 95% confidence intervals [95% CI]. In addition, we examined the association between high body mass index (BMI) exposure and PCOS, including its comorbidity burden across different age groups, taking into account regional variations in high BMI exposure.

**Results:**

In 2021, the global age-standardized prevalence rate (ASPR) of PCOS was 867.7 per 100,000 [95% UI: 618.7–1,195.3], representing a significant increase of 0.75 compared with 1990 [95% CI: 0.71–0.78]. The total number of PCOS cases reached 69.5 million [95% UI: 49.5–95.7 million]. The highest incidence was observed in adolescent females aged 15–19 years and in women of childbearing age aged 30–34 years. Low- and middle-SDI regions exhibited faster increases in incidence (EAPC: 1.76 [95% CI: 1.71–1.81]) compared with high-SDI regions, where incidence remained relatively stable (EAPC: 0.03 [95% CI: −0.14–0.2]). Among countries, the United States had the highest ASPR (1,958.7 per 100,000 [95% UI: 1,463.5–2,556.9]), while Central Europe had the lowest (111.7 per 100,000 [95% UI: 77–159.2]). Southeast Asia showed the fastest-growing PCOS burden (EAPC: 2.22 [95% CI: 2.11–2.32]). Among women exposed to high BMI, the PCOS burden demonstrated age- and region-specific heterogeneity. The overall disease burden decreased with age, particularly when high BMI exposure was below the 40th percentile (P40). Endometrial cancer burden increased with high BMI exposure in women aged 20–44 years, peaking during childbearing age, while the burden plateaued or declined in women aged 44–54 years and those over 55. Ovarian cancer showed an inverted U-shaped association across most age groups, peaking at BMI P40-60 in perimenopausal women. Mental disease burden was positively and synergistically associated with PCOS, with the highest impact at BMI P30–60. In contrast, metabolic disease burden was significantly elevated in postmenopausal women, exhibiting a pattern distinct from that of PCOS.

**Conclusion:**

These findings highlight the urgent need for stratified interventions targeting high-risk populations. Personalized screening and preventive measures can effectively mitigate long-term reproductive and metabolic complications associated with PCOS.

## 1 Introduction

Polycystic ovary syndrome (PCOS), as the most common endocrine and metabolic disease in women of childbearing age, is mainly clinically manifested as hyperandrogenemia, ovulation disorders and polycystic ovary morphology (1, 2). According to the Global Burden of Disease (GBD) 2021 database, PCOS affects approximately 182 million women aged 15–49 worldwide, with an age-standardized prevalence rate of 8.67%. The disease shows significant geographical differences between regions. The prevalence rates in South Asia (17.1%, 95% UI: 15.3–19.0%) and Middle Eastern countries and regions (16.9%, 95% UI: 15.0–18.8%) were much higher than those in Nordic countries and regions (7.3%, 95% UI: 6.4%–8.4%). This geographical difference in PCOS prevalence may stem from the interaction of multiple factors, such as genetic susceptibility, environmental exposure factors, and the accessibility of medical resources, making PCOS an important driver of reproductive health inequality worldwide (3, 4).

Simultaneously, the rapidly rising prevalence of obesity, a risk factor for PCOS, has further increased the burden of disease. The prevalence of obesity (BMI ≥ 30 kg/m^2^) among women of reproductive age increased from 9.2% in 1990 to 21.3% in 2019 (5, 6). In particular, approximately 62.4% of women with PCOS are overweight or obese (BMI ≥ 25 kg/m^2^), and there are significant regional differences: the combined proportion of regions in high-income countries is more than 70%, whereas that in low-income countries is less than 40%. This regional structural difference further exacerbates the “vicious cycle” of PCOS development. Visceral fat accumulation promotes the production of high levels of androgens by activating the ovarian P450c17α enzyme. Hyperinsulinemia further inhibits the synthesis of sex hormone-binding globulin (SHBG), thus forming the “obesity-insulin resistance-reproductive dysfunction” pathophysiological metabolic axis (7). This synergistic effect of metabolic and endocrine disorders has led to a triple “geographic divergence” in the regional burden of long-term PCOS complications in these countries. In the metabolic field, obese women with PCOS in South Asian countries and regions have twice the risk of type 2 diabetes compared to similar populations in Northern Europe (8). In the field of cardiovascular diseases, the disability-adjusted life years (DALYs) rate of PCOS-related myocardial infarction in Middle Eastern countries and regions was 3.1 times that in Western European countries and regions, reflecting significant regional differences in medical access and chronic disease management capacity (9). In terms of cancer risk, the incidence of endometrial cancer in obese women with PCOS in Latin America (34.8/100 000) was 80% higher than the overall incidence in East Asia (19.3/100 000), which may be closely related to the interaction of environmental factors, such as the polymorphism of genes related to estrogen metabolism and the difference in dietary structure (10).

The dynamic evolution of the disease burden associated with PCOS in the past decade further highlights the public health crisis. From 2013 to 2019, the disability-adjusted life years (DALYs) attributable to PCOS-related comorbidities continued to increase at an average annual rate of 2.9% (95% CI: 2.8%–3.0%) globally, with significant regional differences. Sub-Saharan Africa has a relatively weak maternal and child health system, and the growth rate of metabolic syndrome-related disability-adjusted life years (DALYs) is as high as 3.6%. High-income countries (e.g., Western Europe, North America, Australia, and New Zealand) have achieved an average annual decline of 0.7% in disability-adjusted life years (DALYs) for cardiovascular diseases through comprehensive intervention strategies. This differentiation trend can be further quantified by the Sociodemographic Index (SDI): the annual growth rate of the disease burden of PCOS in regions with low SDI (3.1%) is 2.1 times that in regions with high SDI (1.5%), highlighting the continuous expansion of global health inequality related to obesity (11).

Based on the Global Burden of Disease (GBD 2021) database, this study systematically evaluated the global disease burden of polycystic ovary syndrome (PCOS) and its complications from 1990 to 2021 and attempted to construct a spatiotemporal analysis framework of “high BMI risk exposure–PCOS–comorbidity burden” to comprehensively assess the relationships among these factors. By quantifying the high BMI risk exposure levels in different regions for PCOS and its core comorbidities (including the association of disease burden of endometrial cancer, depression and cardiovascular disease), the mediating role of different socioeconomic development levels (SDI) in regional differences in disease burden was revealed, and the trajectory of disease evolution to 2050 under different scenarios was predicted. The research results will provide a key practical basis for optimizing global resource allocation and promoting the formulation of regional precise prevention and control strategies, and contribute to the realization of the Sustainable Development Goal of global reproductive health equity.

## 2 Materials and methods

### 2.1 Overview

The GBD 2021 provides a comprehensive estimation of the global prevalence, incidence, Years Lived with Disability (YLDs), Disability-Adjusted Life Years (DALYs), and Health-Adjusted Life Expectancy for 371 diseases and injuries across 204 countries and territories, along with 811 subnational regions. This analysis used 100,983 data sources. Notably, estimates of the health losses attributable to the COVID−19 pandemic were introduced for the first time in this report (12).

### 2.2 Data sources

The GBD 2021 data were derived from a wide range of sources, including vital registration systems, autopsy records, population censuses, household surveys, disease-specific registries, health service utilization data, and other relevant information (12). According to the database structure, all PCOS-related data were reported exclusively for females, and the values labeled as “both sexes” were identical to those for females, reflecting the female-specific nature of this condition. Reliance on modelled GBD estimates rather than raw country-level registry data entails inherent limitations that may affect the accuracy and robustness of the conclusions. Moreover, the data were constrained by diagnostic heterogeneity, lack of visceral fat measurements, and potential confounding factors.

### 2.3 Disease definition

PCOS is an endocrine-metabolic disorder characterized by hyperandrogenism, ovulatory dysfunction, and polycystic ovarian morphology [American College of Obstetricians and Gynecologists (ACOG), 2018]. As defined in the GBD 2021 study, a diagnosis of PCOS must fulfill at least two of the following core criteria as outlined by the NIH/Rotterdam/AE-PCOS: (1) hyperandrogenism (either clinical or biochemical manifestations); (2) oligo/anovulation; (3) polycystic ovarian morphology (defined as ≥20 follicles in one ovary or an ovarian volume ≥10 ml), while excluding secondary endocrine disorders such as congenital adrenal hyperplasia and hyperprolactinemia. The GBD framework ensures the comparability and methodological consistency of global epidemiological data by integrating and weighting three evidence-based diagnostic systems (GBD Collaborative Network, 2022) (13).

### 2.4 Estimation of disease burden and socio-demographic stratification

Disability-Adjusted Life Years (DALYs) quantified the PCOS burden as the sum of Years Lived with Disability (YLDs), weighted by international disability scores and adjusted via Bayesian models, and Years of Life Lost (YLLs), calculated using 86.6 years as the standard life expectancy (GBD 2021) to account for regional differences (12, 14). The Socio-Demographic Index (SDI), reflecting regional development, combined per capita GNI (PPP), average years of schooling (≥15 years), and total fertility rate, normalized to a 0–100 scale, with higher scores indicating greater development (15, 16). Countries were classified into five SDI quintiles, and age was classified into 20 subgroups from under 5 to over 95 years (12).

### 2.5 Estimated annual percentage change (EAPC)

The EAPC is a highly effective and commonly utilized metric for monitoring trends in indicators such as prevalence and incidence over designated periods. This methodology has been applied in numerous studies (17, 18).

### 2.6 Data processing and disease model

The GBD 2021 study employed a standardized age grouping and underwent standardization processes developed by the Institute for Health Metrics and Evaluation (IHME) to ensure comparability of data across various countries and regions. In examining PCOS, the study utilized the DisMod-MR 2.1 statistical model, which integrates medical records, survey data, and other relevant sources to estimate the disease burden associated with PCOS. This model can automatically address data gaps and diagnostic discrepancies, ultimately yielding accurate estimates categorized by age, region, and year (15, 19).

### 2.7 Statistical methods

To assess the evolving health burden associated with PCOS and infertility, we employed direct standardization to adjust for the structural characteristics of the specific population. Furthermore, Pearson's correlation analysis was used to evaluate the relationship between the age-standardized PCOS disease burden and the SDI. A piecewise regression model was employed to analyze the temporal trend of the disease burden associated with PCOS from 1990 to 2021. Spearman's rank correlation coefficient was utilized to evaluate the relationship between the SDI and the disease burdens of PCOS and infertility. Furthermore, a risk factor analysis was conducted to identify the primary drivers contributing to the infertility burden linked to PCOS (18, 20, 21). This study employed a Bayesian age–period–cohort (BAPC) model to predict the disease burden trends. Constructed using the Integrated Nested Laplace Approximation (INLA) algorithm, the BAPC model offers higher fitting accuracy and narrower confidence intervals than traditional linear methods while retaining the categorical structure of the standard APC framework. Calculations were performed using the BAPC package in R under default settings to ensure reproducibility and robustness (22). This study employed a Bayesian age–period–cohort (BAPC) model to predict the disease burden trends. Constructed using the Integrated Nested Laplace Approximation (INLA) algorithm, the BAPC model offers higher fitting accuracy and narrower confidence intervals than traditional linear methods while retaining the categorical structure of the standard APC framework. Calculations were performed using the BAPC package in R under default settings to ensure reproducibility and robustness (23). The PCOS disease burden was reported per 100,000 individuals with 95% uncertainty interval (UI). Regional associations were assessed using Spearman's rank correlation and locally weighted regression (Loess) to identify non-linear relationships and capture dynamic trends between BMI risk exposure and PCOS complications. Statistical analyses and visualizations were performed using R (version 4.4.3), with a two-sided *p* < 0.05 considered statistically significant (24, 25).

## 3 Result

### 3.1 Global level

From 1990 to 2021, the prevalence of PCOS increased consistently. By the end of 2021, the total number of PCOS cases reported worldwide had reached approximately 69 million [95% (UI): 49,531,420–95,724,479.2], reflecting an ASPR of 867.7 per 100,000 individuals [95% (UI): 618.7–1,195.3], with an EAPC of 0.75 [95% (CI): 0.71–0.78], as detailed in Table 1. Owing to the lack of data on YLLs associated with PCOS, YLDs were consistent with the DALYs. The total number of YLDs and DALYs attributed to PCOS was 607,756.9 [95% (UI): 272,745.2–1,268,607.2], with an ASR of DALYs calculated at 7.6 per 100,000 individuals [95% (UI): 3.4–15.9] and an EAPC of 0.73 [95% (CI): 0.69–0.76], as presented in Table 2.

**Table 1:** Changes in the number of incident cases, prevalent cases, age-standardized incidence rate (ASIR), age-standardized prevalence rate (ASPR), and estimated annual percent change (EAPC) of PCOS globally and in GBD regions in 1990 and 2021.

Location	Incidence				
	1990		2021		EAPC_CI
	Numbers (95% UI)	Age-standardized rate (per 100,000) (95% UI)	Numbers (95% UI)	Age-standardized rate (per 100.000) (95% UI).	
Global	1,476,225.3 (1,057,983.5–2,045,276.9)	24.2 (17.4–33.5)	2,301,505.6 (1,655,989.2–3,167,177.8)	30.7 (22.1–42.4)	0.73 (0.71–0.76)
High SDI	437,301.5 (319,330.7–606,631.9)	58.8 (43–81.4)	494,212.2 (367,071.5–670,948)	70.2 (52–95.3)	0.1 (−0.08–0.28)
High-middle SDI	261,030.5 (187,118.7–358,760.3)	24.2 (17.3–33.5)	305,810 (216,061.4–424,907.9)	34.5 (24.3–48.3)	1.45 (1.36–1.54)
Middle SDI	505,653.9 (359,588–703,114.3)	23.8 (16.9–33.1)	812,669 (572,963.7–1,126,099.7)	37.2 (26.2–51.8)	1.52 (1.48–1.55)
Low-middle SDI	209,333.5 (148,000.1–293,837.3)	14.4 (10.4–20)	481,689 (338,285.7–670,310.3)	22.1 (15.5–30.7)	1.5 (1.44–1.55)
Low SDI	61,881.6 (43,355.6–87,726)	9.9 (7.2–13.9)	205,458.3 (144,223.9–291,379.8)	13.8 (9.8–19.3)	1.14 (1.11–1.17)
World-Bank-High Income	541,762.2 (396,058.4–744,374.6)	61.1 (44.5–84.3)	577,079 (426,601.2–781,420.4)	71.2 (52.5–96.1)	0.11 (−0.04–0.26)
World-Bank-Upper-Middle Income	463,803.1 (332,049.6–640,647.5)	20.4 (14.4–28.4)	603,552.4 (425,593.2–838,856.1)	31.3 (22.1–43.7)	1.41 (1.35–1.47)
World-Bank-Lower-Middle Income	422,468.2 (299,183.9–590,008.8)	17.1 (12.3–23.8)	975,836 (690,009.2–1,352,544.3)	25.7 (18.2–35.7)	1.48 (1.42–1.53)
World-Bank Low Income	47,156.8 (33,104–66,751.5)	12 (8.6–16.7)	143,359.8 (100,369.6–202,455.9)	15.4 (11–21.7)	0.89 (0.87–0.91)
Commonwealth High Income	49,458.5 (35,917.4–66,965.6)	54.9 (39.9–74.5)	68,169.1 (48,878.6–95,023.6)	68.9 (49.3–96.3)	0.56 (0.45–0.66)
Commonwealth-Middle-Income	198,460.5 (141,926.1–276,702.6)	13.8 (10–19.1)	512,268.7 (365,368.6–709,122.7)	21.3 (15.1–29.5)	1.56 (1.48–1.64)
Commonwealth Low Income	25,801.3 (17,924.7–36,698.2)	9.5 (6.7–13.4)	66,869.2 (47,321.7–94,457.9)	13.6 (9.7–19.1)	1.29 (1.22–1.35)
South Asia- WB	172,469.3 (123,555.6–240,042.5)	12.6 (9.1–17.4)	439,909.5 (315,913.9–605,360.4)	20.8 (14.8–28.7)	1.86 (1.73–1.99)
Europe&Central Asia - WB	240,880.2 (172,360.6–331,955)	32.3 (23–44.8)	245,638.9 (174,383.4–339,550.2)	39 (27.6–54.2)	0.88 (0.79–0.98)
East-Asia& Pacific - WB	561,276.6 (402,130.6–778,408.4)	25.8 (18.4–35.7)	745,314.1 (538,524.5–1,032,153.1)	42.4 (30.6–59.1)	1.77 (1.72–1.83)
Middle East & North Africa - WB	104,300.6 (71,982.3–148,482.6)	30.8 (21.5–43.5)	191,408.6 (134,800.9–272,006)	39.5 (27.9–56.2)	0.9 (0.81–0.98)
Sub-Saharan Africa - WB	74,115.1 (51,917.3–105,312.5)	11.3 (8.1–15.9)	231,557.7 (162,753.7–327,991.2)	14.7 (10.5–20.7)	0.76 (0.7–0.83)
Latin America & Caribbean - WB	187,978.5 (130,497.3–267,918.2)	33.7 (23.4–47.9)	242,534.7 (170,902.2–339,080.7)	40 (28.2–56.1)	0.32 (0.22–0.41)
Advanced Health System	567,808.6 (414,557.7–781,167.8)	49.1 (35.8–67.9)	608,234 (449,376.3–823,362.3)	59.7 (43.9–80.6)	0.42 (0.31–0.52)
Basic Health System	640,190.4 (455,028.5–889,468.9)	23.1 (16.4–32.2)	961,899.9 (678,292–1,340,880.5)	37.3 (26.2–52.1)	1.6 (1.55–1.66)
Limited Health System	251,237.2 (177,968.6–352,944.5)	12.9 (9.4–18)	669,132.2 (477,165.3–930,045.3)	19.7 (14–27.3)	1.52 (1.45–1.59)
Minimal Health System	15,964.9 (11,131.5–22,579.7)	9.4 (6.7–13.1)	60,572.4 (42,401.7–85,645.5)	13.3 (9.4–18.7)	1.17 (1.1–1.24)
Asia	793,636 (568,761.9–1,097,309.8)	21 (15.1–29)	1,294,489.7 (927,249.6–1,790,026.9)	30.7 (21.9–42.5)	1.36 (1.32–1.4)
Central Asia	5,347.8 (3,686.1–7,702.4)	6.8 (4.7–9.7)	8,122.3 (5,654.1–11,355.5)	9.1 (6.3–12.7)	1.05 (0.99–1.12)
Southeast Asia	174,333.2 (122,499–243,431.4)	29.3 (20.6–40.8)	357,262.3 (254,498.6–495,461.4)	53.6 (37.9–74.9)	2.24 (2.14–2.35)
East Asia	218,485.8 (154,343.7–301,132.9)	15.6 (11.1–21.8)	268,458.5 (190,157.1–373,877.4)	27.3 (19.2–38.4)	1.79 (1.64–1.95)
Africa	122,302.2 (85,897.2–174,431.5)	15 (10.7–21.2)	319,908.8 (224,226.3–449,944.6)	17.8 (12.6–25)	0.43 (0.36–0.51)
Central Africa	7,931.2 (5,511.5–11,340.2)	9.5 (6.8–13.4)	29,218 (20,173.9–41,544.2)	13 (9.2–18.3)	0.91 (0.77–1.04)
Eastern Africa	26,106.6 (18,300.8–37,162.2)	11.4 (8.2–15.9)	79,974.7 (56,147.7–113,877.5)	15.1 (10.7–21.2)	0.97 (0.91–1.03)
Northern Africa	48,437 (33,514.5–69,161.3)	30.8 (21.5–44)	89,112.6 (62,107.6–127,629.8)	40.2 (28.3–57.7)	0.84 (0.81–0.87)
Western Africa	22,102.2 (15,436.6–31,453.4)	10.3 (7.4–14.3)	81,827.7 (57,545.3–116,614.9)	14.1 (10–19.8)	0.79 (0.65–0.94)
North America	134,114.4 (94,942.4–187,145.5)	58.1 (40.9–81.3)	203,410 (150,001.7–271,131.6)	73.2 (54.4–96.6)	−0.55 (−1.03 to −0.06)
North Africa and Middle East	128,593.5 (88,937.3–183,308.6)	28.7 (20.1–40.6)	242,427.1 (171,058.1–342,784.6)	37.3 (26.3–52.8)	0.92 (0.88–0.96)
Southern Sub-Saharan Africa	11,799.7 (8,261–16,787.8)	17.2 (12.1–24.3)	18,377.8 (12,888.4–26,038)	21 (14.7–29.8)	0.63 (0.54–0.72)
Eastern Sub-Saharan Africa	25,902 (18,214–36,986.2)	10.4 (7.5–14.6)	76,605.1 (53,945.1–108,540.5)	13.1 (9.3–18.4)	0.8 (0.76–0.83)
Western Sub-Saharan Africa	24,710.3 (17,219.7–35,159.5)	10.3 (7.4–14.4)	92,826.2 (65,226.5–132,560.9)	14.2 (10.1–20)	0.79 (0.64–0.93)
Central Sub-Saharan Africa	6,063 (4,247.4–8,685.3)	8.8 (6.3–12.4)	23,871.9 (16,637.7–33,976)	13 (9.2–18.4)	1.21 (1.06–1.36)
America	320,708.9 (225,689.3–448,079.9)	40.9 (28.8–57.2)	444,934.9 (330,613.1–592,182.4)	50.4 (37.3–67.5)	0.01 (−0.23–0.25)
Tropical Latin America	22,284.6 (15,199.8–32,004)	11.7 (8–16.7)	23,769.1 (16,611.4–32,960.1)	12.3 (8.4–17.2)	−0.27 (−0.44 to −0.1)
Central Latin America	119,114.9 (81,948.2–168,844.6)	53.7 (37–75.9)	143,013.7 (100,629.4–200,290.7)	57.2 (40–80)	−0.17 (−0.34–0)
Latin America and Caribbean	175,877 (121,576.9–250,897.8)	34.7 (24.1–49.4)	221,379.9 (155,474.9–309,629)	40.4 (28.3–56.6)	0.22 (0.11–0.34)
Andean Latin America	25,250.9 (17,294.5–36,023.1)	50 (34.4–71)	42,634.6 (29,473.4–60,429.4)	64.7 (44.7–91.9)	0.79 (0.69–0.89)
Southern-Latin America	12,429.8 (8,602.2–17,727.2)	23.3 (16.1–33.3)	21,574.4 (15,329.6–30,970)	36.6 (25.9–52.4)	1.42 (1.22–1.63)
United-States-of America	127,848.5 (90,504–178,310.7)	61.3 (43.2–85.9)	194,555.5 (142,842.7–258,038.3)	76.8 (57.1–100.9)	−0.64 (−1.15 to −0.12)
Region-of-the-Americas	320,708.9 (225,689.3–448,079.9)	40.9 (28.8–57.2)	444,934.9 (330,613.1–592,182.4)	50.4 (37.3–67.5)	0.01 (−0.23–0.25)
American Samoa	22 (15.5–30.8)	37.2 (26.2–52.2)	31.4 (21.7–45.9)	52 (36.2–75.8)	0.96 (0.76–1.16)
Oceania	2,013.1 (1,401.2–2,847)	23.8 (16.7–33.5)	5,300.9 (3,692.1–7,439.8)	32.9 (23–46.3)	0.81 (0.66–0.96)
Europe	237,069.8 (169,674.3–326,634.9)	34.5 (24.5–47.9)	239,357.7 (169,918.3–330,720.8)	42.9 (30.4–59.5)	0.98 (0.88–1.07)
Eastern Europe	8,328.3 (5,913.3–11,639.2)	4.2 (3–5.9)	7,596.1 (5,457.5–10,592.6)	5.3 (3.7–7.5)	0.89 (0.85–0.93)
Western Europe	206,740.9 (146,330.9–286,172.1)	69.9 (49.2–97.8)	206,192.2 (145,653.5–286,689.1)	75.2 (53.1–105)	0.14 (0.1–0.18)
Central Europe	4,244.9 (2,905.8–6,223.9)	3.6 (2.5–5.3)	3,175.5 (2,221.1–4,441)	4.3 (3–6.1)	0.53 (0.46–0.59)

Location	Prevalence				
	1990		2021		EAPC_CI
	Numbers (95% UI)	Age-standardized rate (per 100,000) (95% UI)	Numbers (95% UI)	Age-standardized rate (per 100,000) (95% UI)	
Global	36,651,157.2 (26,227,943.2–50,603,929.8)	676.8 (485.5–932.6)	69,473,252.4 (49,531,420–95,724,479.2)	867.7 (618.7–1,195.3)	0.75 (0.71–0.78)
High SDI	13,783,058.5 (10,021,809.4–19,223,038.3)	1,479.8 (1,075.7–2,053.4)	17,573,919.8 (12,981,150.8–23,876,520.6)	1,720.7 (1,270.5–2,331.7)	0.03 (−0.14–0.2)
High-middle SDI	7,047,030 (4,981,606.3–9,727,416.6)	621.7 (439.3–859.5)	11,180,894.9 (7,874,794.9–15,590,881.4)	877.5 (616.7–1,221.5)	1.16 (1.11–1.21)
Middle SDI	10,580,143.2 (7,410,905.3–14,674,450.8)	576.7 (404.4–798.9)	24,613,369.9 (17,452,531.6–34,074,253.7)	970.2 (687.2–1,340.9)	1.76 (1.71–1.81)
Low-middle SDI	4,094,311.3 (2,868,780.9–5,765,430.7)	368.3 (260.4–514.5)	12,118,405.4 (8,434,181–17,027,257.3)	592.4 (413–831.8)	1.67 (1.62–1.71)
Low SDI	1,121,931 (784,807–1,611,458.4)	252.4 (178.4–359.2)	3,938,251.3 (2,745,129.9–5,589,751.2)	361.6 (255.3–509.8)	1.24 (1.21–1.28)
World-Bank-High Income	16,748,910.2 (12,163,633.3–22,944,490.4)	1,558.1 (1,131.3–2,128.3)	20,132,268.6 (14,827,038.9–27,314,275.5)	1,763.5 (1,300.4–2,396.9)	−0.01 (−0.16–0.14)
World-Bank-Upper-Middle Income	10,574,147.8 (7,408,942.4–14,750,935.7)	485.9 (340.5–676.8)	20,345,626.5 (14,332,447.6–28,213,302)	812.6 (570.8–1,123.3)	1.64 (1.55–1.74)
World-Bank-Lower-Middle Income	8,440,600.8 (5,965,275.5–11,808,115.2)	434.9 (307.7–606.4)	26,163,393 (18,272,756.9–36,695,088.6)	716.5 (501.1–1,005.3)	1.85 (1.77–1.93)
World-Bank Low Income	862,591.4 (600,137.6–1,229,503.7)	315.3 (221.7–446.3)	2,783,244.8 (1,917,591.7–3,959,813.4)	416.5 (287.9–587.3)	0.96 (0.94–0.99)
Commonwealth High Income	1,607,542.9 (1,166,394.2–2,225,879.2)	1,385.5 (1,002–1,920.4)	2,378,784 (1,706,949.9–3,311,197.9)	1,752.5 (1,257.7–2,428.2)	0.56 (0.46–0.67)
Commonwealth-Middle-Income	4,046,031.8 (2,880,612.3–5,648,186.5)	351.7 (252.6–489.4)	13,473,435.8 (9,409,885.7–18,889,846.4)	595.4 (416.6–833.7)	1.92 (1.84–1.99)
Commonwealth Low Income	448,535.3 (311,553.3–649,696.5)	235.6 (164.5–337.4)	1,404,409.1 (973,640.1–1,990,177.4)	352 (244.3–497.7)	1.42 (1.36–1.47)
South Asia- WB	3,511,154.2 (2,504,013.6–4,894,814.3)	321.5 (230.9–446.3)	11,848,596.9 (8,333,792.5–16,574,375.3)	570.5 (401.4–797)	2.15 (2.04–2.27)
Europe & Central Asia - WB	7,614,157.3 (5,371,668.9–10,561,179.5)	890.8 (628–1,235.1)	8,761,385.4 (6,157,123.2–12,324,344.8)	1,024.5 (717.6–1,443.8)	0.38 (0.35–0.41)
East-Asia& Pacific - WB	13,980,093.9 (10,018,861.1–19,417,295.3)	691.5 (497.2–961.3)	25,534,892.8 (18,404,255–35,803,825.3)	1,093.8 (782.4–1,533.4)	1.61 (1.55–1.66)
Middle East & North Africa - WB	1,987,856.5 (1,387,094.8–2,815,222.8)	806.9 (563.6–1,142.9)	5,367,433.4 (3,762,038.5–7,603,740.7)	1,032.4 (723.5–1,463.6)	0.9 (0.82–0.99)
Sub-Saharan Africa - WB	1,340,479.3 (935,579–1,925,004.9)	290.6 (204.6–414.4)	4,539,775.9 (3,134,250.4–6,466,291.6)	397.7 (278.4–563.1)	0.93 (0.86–1.01)
Latin America & Caribbean - WB	3,721,323.6 (2,562,708.7–5,210,753.5)	827.7 (570.9–1,156.3)	7,008,084.4 (4,942,278.9–9,743,064.9)	1,007.2 (709.2–1,399.4)	0.34 (0.23–0.44)
Advanced Health System	17,349,361.4 (12,584,994.4–23,781,916.8)	1,271.4 (921.5–1,738.6)	21,154,221.8 (15,573,659.3–28,771,671.3)	1,484.3 (1,096.8–2,026.7)	0.13 (−0.01–0.27)
Basic Health System	14,066,187.7 (9,900,136.1–19,602,002)	568.1 (400.2–790.6)	30,516,465.6 (21,616,756.1–42,543,674.2)	959.8 (677.4–1,335.9)	1.75 (1.67–1.83)
Limited Health System	4,925,706.2 (3,482,413.5–6,925,214.7)	328.3 (234.8–459)	16,662,344.4 (11,625,899.1–23,359,835.5)	547.7 (382.8–765.4)	1.87 (1.79–1.94)
Minimal Health System	285,218.6 (199,008.7–413,707.9)	244.7 (172–350.8)	1,091,809.4 (756,623.3–1,554,184.5)	347.7 (242.2–492.1)	1.11 (1.05–1.18)
Asia	18,598,841.4 (13,403,782.7–25,748,422.2)	566.7 (409.5–783.9)	40,591,623.1 (28,924,411.8–56,833,433.1)	843.1 (599.1–1,178)	1.45 (1.39–1.5)
Central Asia	118,817.9 (79,820.2–174,950)	175.7 (118.4–257.8)	237,958.9 (163,368.4–333,464.7)	240.5 (164.6–337.3)	1.12 (1.06–1.18)
Southeast Asia	3,682,948.8 (2,589,198.8–5,201,509.9)	773.1 (546.2–1,094.3)	10,520,027.7 (7,378,813.9–14,809,823.5)	1,404.1 (984.5–1,974.7)	2.22 (2.11–2.32)
East Asia	5,607,180.9 (3,957,250.2–7,863,221.6)	408.1 (290–572.2)	10,490,358.5 (7,423,407.5–14,808,757.1)	740.8 (519.6–1,039.3)	2.05 (1.88–2.22)
Africa	2,299,016.3 (1,591,793.5–3,250,396.7)	398 (275.2–561.4)	6,872,240 (4,758,221.4–9,660,622.2)	508.6 (354–715.6)	0.74 (0.67–0.81)
Central Africa	145,214.1 (99,833.7–212,042.4)	248 (172–357.8)	546,587 (373,630.6–777,685.4)	339.5 (233.4–482.5)	0.9 (0.77–1.02)
Eastern Africa	462,221.8 (321,386.7–665,530.8)	299 (210.3–428)	1,580,095.5 (1,088,569–2,252,159.8)	410.8 (287.2–584.8)	1.08 (1.01–1.14)
Northern Africa	963,191.4 (662,484.9–1,371,449.6)	817.1 (562.9–1,162.1)	2,345,085.7 (1,633,867.5–3,341,591.9)	1,076.4 (750.2–1,533.8)	0.88 (0.85–0.91)
Western Africa	390,480.1 (272,779.4–565,587.5)	247.6 (174.5–354.7)	1,535,927.4 (1,069,592.2–2,198,735.1)	374.4 (264.1–532.4)	1.09 (0.92–1.27)
North America	4,469,727 (3,157,989.7–6,274,823.9)	1,483.7 (1,046.5–2,088.3)	6,362,255.1 (4,743,009.3–8,324,162.7)	1,855.8 (1,382.4–2,426.5)	−0.52 (−1.01 to −0.04)
North Africa and Middle East	2,463,301.1 (1,707,181.3–3,501,813.4)	754.3 (523–1,071.5)	6,673,431.5 (4,672,056.3–9,434,543.5)	990.2 (693.3–1,399.1)	1 (0.96–1.05)
Southern Sub-Saharan Africa	232,808.6 (160,575–334,631.4)	449.2 (310.3–643.1)	480,389.6 (328,945.2–679,589.3)	551.2 (377.3–779.2)	0.68 (0.58–0.78)
Eastern Sub-Saharan Africa	448,071 (310,256.9–646,236.2)	266.9 (188.2–383.1)	1,438,942.4 (1,001,296.8–2,063,339.5)	342.7 (242–486.2)	0.83 (0.8–0.87)
Western Sub-Saharan Africa	438,598.3 (307,139.7–633,877.8)	252.3 (177.7–360.9)	1,741,945.4 (1,209,889.2–2,493,010.7)	378 (265.9–537)	1.06 (0.89–1.23)
Central Sub-Saharan Africa	110,521.9 (76,168.2–160,819.7)	226.9 (157.8–329.5)	444,120.8 (305,191.6–640,542)	337.7 (233.3–485.4)	1.23 (1.1–1.37)
America	8,155,097.4 (5,773,666.2–11,350,176.3)	1,100.4 (779.7–1,529.1)	13,334,863.9 (9,771,560.1–18,081,294.3)	1,286.3 (942.5–1,744.3)	−0.2 (−0.46–0.06)
Tropical Latin America	448,844.2 (304,409–647,659.2)	283.9 (192.7–407.2)	746,471.7 (514,677–1,057,462.8)	308.2 (211.9–438.9)	−0.16 (−0.32–0)
Central Latin America	2,295,522 (1,578,370.6–3,199,568.8)	1,382.5 (952.5–1,915.8)	4,072,957.2 (2,852,240.6–5,662,452.4)	1,518.7 (1,063–2,110.8)	−0.1 (−0.26–0.05)
Latin America and Caribbean	3,433,164.3 (2,347,793.1–4,820,492.3)	855.4 (587.2–1,200.7)	6,353,275.2 (4,457,452.4–8,841,849.3)	1,012.5 (710.1–1,408.9)	0.22 (0.1–0.35)
Andean Latin America	466,365.8 (322,494–653,112.7)	1,230.4 (850.9–1,717.8)	1,172,864.5 (808,337.4–1,649,165.9)	1,662.5 (1,148.2–2,338.2)	1.02 (0.94–1.1)
Southern-Latin America	296,321.5 (204,840.7–431,020.1)	600.5 (416.5–874.7)	667,604.8 (469,021.5–956,761.4)	950.5 (666.4–1,360.4)	1.45 (1.23–1.66)
United-States-of America	4,254,919.1 (3,008,393.8–5,969,707)	1,568 (1,107.3–2,207)	6,051,074.9 (4,526,937.1–7,901,919.8)	1,958.7 (1,463.5–2,556.9)	−0.6 (−1.12 to −0.09)
Region-of-the-Americas	8,155,097.4 (5,773,666.2–11,350,176.3)	1,100.4 (779.7–1,529.1)	13,334,863.9 (9,771,560.1–18,081,294.3)	1,286.3 (942.5–1,744.3)	−0.2 (−0.46–0.06)
American Samoa	490.6 (341.6–691.2)	989 (687–1,397)	662.3 (463.1–968.6)	1,368.2 (952.6–1,998.6)	0.93 (0.74–1.12)
Oceania	40,226.7 (27,505–56,891.6)	621.1 (428.1–873.4)	124,484.3 (86,570.2–177,516.4)	870.3 (605–1,243.3)	0.84 (0.67–1.02)
Europe	7,534,911.5 (5,318,909.7–10,451,156.5)	932.8 (657.5–1,292.7)	8,587,063.7 (6,032,644.8–12,086,938.9)	1,104.7 (774.4–1,555.8)	0.48 (0.46–0.51)
Eastern Europe	237,422.1 (160,523.3–339,244.5)	103.8 (70–149.6)	265,678.7 (185,374.4–381,763)	132.4 (91.2–190.5)	0.96 (0.92–1.01)
Western Europe	6,815,142.5 (4,796,290.8–9,455,180.2)	1,751.6 (1,233.8–2,431.9)	7,455,929.3 (5,232,259.4–10,460,556.8)	1,944.3 (1,361.7–2,726.7)	0.21 (0.14–0.27)
Central Europe	116,152 (76,721–173,921.5)	91.9 (60.8–137.8)	119,149.2 (82,050.4–168,514)	111.7 (77–159.2)	0.58 (0.52–0.64)

**Table 2:** Changes in DALYs, YLDs, ASR_DALYs, ASR_DALYs, and EAPC of PCOS in the world and GBD regions in 1990 and 2021.

Location	DALYs				
	1990		2021		EAPC_CI
	Numbers (95% UI)	Age-standardized rate (per 100,000) (95% UI)	Numbers (95% UI)	Age-standardized rate (per 100,000) (95% UI)	
Global	323,798.6 (144,342.1–675,926.8)	6 (2.7–12.4)	607,756.9 (272,745.2–1,268,607.2)	7.6 (3.4–15.9)	0.73 (0.69–0.76)
High SDI	122,087.1 (55,322.3–254,195)	13.1 (5.9–27.2)	154,313.3 (70,664.1–314,967.1)	15.2 (7–31)	0.01 (−0.16–0.18)
High-middle SDI	61,793.6 (27,718.6–128,454.5)	5.4 (2.4–11.3)	97,184.5 (43,255.1–205,403.8)	7.7 (3.4–16.1)	1.16 (1.1–1.21)
Middle SDI	93,350.2 (41,377–195,580.3)	5.1 (2.2–10.7)	214,898.8 (95,592.6–450,661.8)	8.5 (3.8–17.8)	1.75 (1.69–1.81)
Low-middle SDI	36,511.8 (15,811.4–76,977.5)	3.3 (1.4–6.8)	106,509.7 (46,621.4–223,924.9)	5.2 (2.3–10.9)	1.62 (1.59–1.66)
Low SDI	9,836.5 (4,213.8–20,814.4)	2.2 (0.9–4.7)	34,425.2 (14,799.7–72,773.1)	3.1 (1.4–6.6)	1.23 (1.2–1.25)
World-Bank-High Income	144,805.2 (66,290.7–301,489.4)	13.8 (6.3–28.7)	173,638.6 (79,806.4–356,835.8)	15.6 (7.1–31.9)	−0.02 (−0.17–0.13)
World-Bank-Upper-Middle Income	95,385.9 (41,793.4–200,416.2)	4.2 (1.9–8.9)	182,847 (80,899.9–382,594)	7.1 (3.1–14.8)	1.64 (1.54–1.74)
World-Bank-Lower-Middle Income	72,754.3 (31,705.3–151,658.8)	3.9 (1.7–8)	222,619.6 (97,700.5–466,783.1)	6.3 (2.8–13.2)	1.8 (1.72–1.88)
World-Bank Low Income	8,090.6 (3,447.9–16,957)	2.7 (1.2–5.7)	25,782.9 (11,268.1–54,535.9)	3.6 (1.6–7.6)	0.97 (0.94–0.99)
Commonwealth High Income	14,290.3 (6,398.2–29,720.3)	12.3 (5.5–25.6)	20,984.7 (9,551.9–43,821.4)	15.5 (7–32.3)	0.55 (0.45–0.66)
Commonwealth-Middle-Income	35,888.3 (15,693.7–75,336.6)	3.1 (1.4–6.5)	117,820.5 (51,622.1–246,275.3)	5.2 (2.3–10.9)	1.87 (1.8–1.95)
Commonwealth Low Income	3,955.9 (1,685.1–8,333.6)	2.1 (0.9–4.3)	12,239.4 (5,284.6–25,711.2)	3.1 (1.3–6.5)	1.39 (1.34–1.44)
South Asia- WB	31,269.1 (13,687.6–66,187.9)	2.8 (1.2–6)	103,813.6 (45,375.4–217,015.1)	5 (2.2–10.4)	2.1 (1.99–2.21)
Europe & Central Asia - WB	67,970.2 (30,712.8–141,096.5)	8 (3.6–16.5)	77,466.9 (35,037.9–161,551.3)	9.1 (4.1–19)	0.37 (0.34–0.4)
East-Asia& Pacific - WB	121,819.4 (53,563.3–248,621.9)	6 (2.6–12.2)	221,741.8 (99,008.6–453,970.7)	9.5 (4.3–19.6)	1.61 (1.56–1.67)
Middle East & North Africa - WB	18,065.2 (7,904.8–38,138.7)	7.3 (3.2–15.3)	47,725.9 (21,256.2–101,198)	9.2 (4.1–19.5)	0.87 (0.79–0.96)
Sub-Saharan Africa - WB	11,697.6 (4,975.5–24,792.7)	2.5 (1.1–5.3)	39,456.1 (17,164.4–83,265.7)	3.4 (1.5–7.2)	0.92 (0.84–1)
Latin America & Caribbean - WB	32,765.7 (14,429.1–67,730.8)	7.3 (3.2–15)	60,944 (26,973–126,410)	8.8 (3.9–18.2)	0.32 (0.22–0.43)
Advanced Health System	153,790.1 (70,341.5–319,860.6)	11.3 (5.2–23.4)	185,952.4 (85,392.4–382,110.3)	13.1 (6–26.9)	0.12 (−0.02–0.26)
Basic Health System	123,699.3 (54,098.2–260,032.5)	5 (2.2–10.5)	266,136.4 (118,158.6–556,135.2)	8.4 (3.7–17.6)	1.75 (1.67–1.83)
Limited Health System	43,614.4 (18,973.8–91,859)	2.9 (1.3–6.1)	145,769.4 (63,565.2–306,376)	4.8 (2.1–10)	1.83 (1.76–1.9)
Minimal Health System	2,475.3 (1,058.1–5,180.5)	2.1 (0.9–4.4)	9,473.2 (4,080.6–20,118.6)	3 (1.3–6.3)	1.11 (1.03–1.18)
Asia	163,096.6 (71,792–338,188.2)	4.9 (2.2–10.3)	354,057 (156,107–731,797.9)	7.4 (3.2–15.2)	1.44 (1.39–1.5)
Central Asia	1,049.1 (438.1–2,236.9)	1.5 (0.6–3.3)	2,079.4 (886.9–4,469.5)	2.1 (0.9–4.5)	1.11 (1.05–1.17)
Southeast Asia	32,853.8 (14,429.1–66,791.8)	6.9 (3–14)	92,605.7 (41,149–191,024.1)	12.4 (5.5–25.5)	2.18 (2.09–2.28)
East Asia	48,225.9 (20,818.6–100,143)	3.5 (1.5–7.3)	89,991.4 (39,441.8–185,701.9)	6.4 (2.8–13.2)	2.06 (1.89–2.24)
Africa	20,445.4 (8,874.8–43,350.5)	3.5 (1.5–7.4)	60,192.9 (26,651.9–127,793.2)	4.4 (2–9.4)	0.69 (0.62–0.76)
Central Africa	1,262.2 (537.3–2,655.3)	2.1 (0.9–4.5)	4,768.9 (2,060.6–9,892.1)	2.9 (1.3–6.1)	0.89 (0.75–1.03)
Eastern Africa	4,043.1 (1,726.4–8,543.1)	2.6 (1.1–5.5)	13,730.2 (6,046.1–28,860.7)	3.5 (1.6–7.5)	1.07 (1.01–1.12)
Northern Africa	8,789 (3,855.5–18,483.1)	7.4 (3.2–15.6)	20,848.8 (9,244.5–43,732)	9.6 (4.2–20.1)	0.81 (0.79–0.84)
Western Africa	3,395.8 (1,453–7,141.5)	2.1 (0.9–4.5)	13,366.3 (5,760.7–28,368.4)	3.2 (1.4–6.9)	1.09 (0.91–1.26)
North America	39,977.3 (17,689.4–82,640.8)	13.3 (5.9–27.3)	56,162.3 (25,718.6–113,710.7)	16.4 (7.6–33.3)	−0.54 (−1.01 to −0.05)
North Africa and Middle East	22,386.5 (9,812.9–47,102.6)	6.8 (3–14.3)	59,116.3 (26,476.8–125,706.8)	8.8 (3.9–18.7)	0.96 (0.9–1.01)
Southern Sub-Saharan Africa	2,047.4 (881.8–4,435.2)	3.9 (1.7–8.5)	4,157.7 (1,795.1–8,746)	4.8 (2.1–10)	0.64 (0.54–0.74)
Eastern Sub-Saharan Africa	3,903.3 (1,645.1–8,198.6)	2.3 (1–4.9)	12,497.1 (5,380.7–26,310.2)	3 (1.3–6.2)	0.82 (0.79–0.86)
Western Sub-Saharan Africa	3,816.2 (1,629–8,015.4)	2.2 (0.9–4.6)	15,176.8 (6,521.5–32,235.9)	3.3 (1.4–6.9)	1.05 (0.88–1.23)
Central Sub-Saharan Africa	958.3 (406.3–1,975.2)	1.9 (0.8–4.1)	3,861.9 (1,673.7–7,930.5)	2.9 (1.3–6)	1.23 (1.08–1.38)
America	72,424.3 (32,262–149,675.4)	9.8 (4.3–20.2)	116,794.5 (53,755.4–243,772.3)	11.3 (5.2–23.5)	−0.22 (−0.49–0.04)
Tropical Latin America	4,011.1 (1,701.1–8,427.3)	2.5 (1.1–5.3)	6,557.7 (2,825.1–13,831)	2.7 (1.2–5.7)	−0.18 (−0.34–−0.01)
Central Latin America	20,163.7 (9,003.8–42,043)	12.1 (5.4–25.3)	35,314.1 (15,577.8–73,735.7)	13.2 (5.8–27.5)	−0.12 (−0.27–0.04)
Latin America and Caribbean	30,210.5 (13,328.4–62,616.6)	7.5 (3.3–15.6)	55,162.5 (24,274.2–114,672.1)	8.8 (3.9–18.3)	0.21 (0.09–0.33)
Andean Latin America	4,055.9 (1,797.3–8,748.4)	10.7 (4.8–23)	10,129.2 (4,423.9–21,176.6)	14.4 (6.3–30)	1 (0.92–1.08)
Southern-Latin America	2,627.8 (1,179.4–5,421.3)	5.3 (2.4–11)	5,893.5 (2,574.8–12,247.5)	8.4 (3.7–17.4)	1.44 (1.23–1.65)
United-States-of America	38,051.1 (16,838.4–78,573.6)	14 (6.2–28.8)	53,388.1 (24,521.8–107,618.8)	17.3 (8–34.9)	−0.62 (−1.13 to −0.1)
Region-of-the-Americas	72,424.3 (32,262–149,675.4)	9.8 (4.3–20.2)	116,794.5 (53,755.4–243,772.3)	11.3 (5.2–23.5)	−0.22 (−0.49–0.04)
American Samoa	4.3 (1.9–9)	8.7 (3.8–18.2)	5.8 (2.5–12.2)	12 (5.2–25.3)	0.92 (0.73–1.1)
Oceania	352.2 (160.6–738.9)	5.4 (2.5–11.3)	1,083.5 (471.3–2,285.6)	7.6 (3.3–15.9)	0.84 (0.67–1.01)
Europe	67,270.7 (30,434–139,695.2)	8.3 (3.8–17.3)	75,944 (34,374.6–158,081.6)	9.8 (4.4–20.5)	0.47 (0.45–0.5)
Eastern Europe	2,097.7 (868.6–4,466.8)	0.9 (0.4–2)	2,323.7 (970–4,890.4)	1.2 (0.5–2.5)	0.95 (0.91–1)
Western Europe	60,788.9 (27,584.9–126,652.6)	15.6 (7.1–32.5)	66,041.5 (29,857.2–136,985.1)	17.3 (7.8–35.9)	0.21 (0.15–0.27)
Central Europe	1,014.7 (425.9–2,088.9)	0.8 (0.3–1.7)	1,032.7 (442.9–2,156.6)	1 (0.4–2)	0.58 (0.53–0.63)

Location	YLDs				
	1990		2021		EAPC_CI
	Numbers (95% UI)	Age-standardized rate (per 100,000) (95% UI)	Numbers (95% UI)	Age-standardized rate (per 100.000) (95% UI)	
Global	323,798.589482073 (144,342.1–675,926.8)	6 (2.7–12.4)	607,756.9 (272,745.2–1,268,607.2)	7.6 (3.4–15.9)	0.73 (0.69–0.76)
High SDI	122,087.069310582 (55,322.3–254,195)	13.1 (5.9–27.2)	154,313.3 (70,664.1–314,967.1)	15.2 (7–31)	0.01 (−0.16–0.18)
High-middle SDI	61,793.5829121228 (27,718.6–128,454.5)	5.4 (2.4–11.3)	97,184.5 (43,255.1–205,403.8)	7.7 (3.4–16.1)	1.16 (1.1–1.21)
Middle SDI	93,350.1643457733 (41,377–195,580.3)	5.1 (2.2–10.7)	214,898.8 (95,592.6–450,661.8)	8.5 (3.8–17.8)	1.75 (1.69–1.81)
Low-middle SDI	36,511.8299999773 (15,811.4–76,977.5)	3.3 (1.4–6.8)	106,509.7 (46,621.4–223,924.9)	5.2 (2.3–10.9)	1.62 (1.59–1.66)
Low SDI	9,836.51670213337 (4,213.8–20,814.4)	2.2 (0.9–4.7)	34,425.2 (14,799.7–72,773.1)	3.1 (1.4–6.6)	1.23 (1.2–1.25)
World-Bank-High Income	148,344.057971075 (67,911.7–308,843.6)	13.8 (6.3–28.7)	176,971.3 (81,338.8–363,690.1)	15.6 (7.1–31.9)	−0.02 (−0.17–0.13)
World-Bank-Upper-Middle Income	92,337.6034404174 (40,457.1–194,018.7)	4.2 (1.9–8.9)	176,013.8 (77,879.2–368,272.8)	7.1 (3.1–14.8)	1.64 (1.54–1.74)
World-Bank-Lower-Middle Income	75,357.6971773956 (32,839.8–157,087.2)	3.9 (1.7–8)	230,128.8 (100,994.3–482,517.2)	6.3 (2.8–13.2)	1.8 (1.72–1.88)
World-Bank Low Income	7,537.83570331432 (3,212.6–15,799)	2.7 (1.2–5.7)	24,214.9 (10,583.8–51,228.4)	3.6 (1.6–7.6)	0.95 (0.92–0.98)
Commonwealth High Income	14,290.2893678241 (6,398.2–29,720.3)	12.3 (5.5–25.6)	20,984.7 (9,551.9–43,821.4)	15.5 (7–32.3)	0.55 (0.45–0.66)
Commonwealth-Middle-Income	35,888.3394775411 (15,693.7–75,336.6)	3.1 (1.4–6.5)	117,820.5 (51,622.1–246,275.3)	5.2 (2.3–10.9)	1.87 (1.8–1.95)
Commonwealth Low Income	3,955.91199916162 (1,685.1–8,333.6)	2.1 (0.9–4.3)	12,239.4 (5,284.6–25,711.2)	3.1 (1.3–6.5)	1.39 (1.34–1.44)
South Asia- WB	31,269.0991459791 (13,687.6–66,187.9)	2.8 (1.2–6)	103,813.6 (45,375.4–217,015.1)	5 (2.2–10.4)	2.1 (1.99–2.21)
Europe & Central Asia - WB	67,970.2186712524 (30,712.8–141,096.5)	8 (3.6–16.5)	77,466.9 (35,037.9–161,551.3)	9.1 (4.1–19)	0.37 (0.34–0.4)
East-Asia& Pacific - WB	121,819.36675242 (53,563.3–248,621.9)	6 (2.6–12.2)	221,741.8 (99,008.6–453,970.7)	9.5 (4.3–19.6)	1.61 (1.56–1.67)
Middle East & North Africa - WB	18,065.1887639013 (7,904.8–38,138.7)	7.3 (3.2–15.3)	47,725.9 (21,256.2–101,198)	9.2 (4.1–19.5)	0.87 (0.79–0.96)
Sub-Saharan Africa - WB	11,697.5579656459 (4,975.5–24,792.7)	2.5 (1.1–5.3)	39,456.1 (17,164.4–83,265.7)	3.4 (1.5–7.2)	0.92 (0.84–1)
Latin America & Caribbean - WB	32,765.70733679 (14,429.1–67,730.8)	7.3 (3.2–15)	60,944 (26,973–126,410)	8.8 (3.9–18.2)	0.32 (0.22–0.43)
Advanced Health System	153,790.104897037 (70,341.5–319,860.6)	11.3 (5.2–23.4)	185,952.4 (85,392.4–382,110.3)	13.1 (6–26.9)	0.12 (−0.02–0.26)
Basic Health System	123,699.300319931 (54,098.2–260,032.5)	5 (2.2–10.5)	266,136.4 (118,158.6–556,135.2)	8.4 (3.7–17.6)	1.75 (1.67–1.83)
Limited Health System	43,614.4187239326 (18,973.8–91,859)	2.9 (1.3–6.1)	145,769.4 (63,565.2–306,376)	4.8 (2.1–10)	1.83 (1.76–1.9)
Minimal Health System	2,475.33932968792 (1,058.1–5,180.5)	2.1 (0.9–4.4)	9,473.2 (4,080.6–20,118.6)	3 (1.3–6.3)	1.11 (1.03–1.18)
Asia	163,096.641322195 (71,792–338,188.2)	4.9 (2.2–10.3)	354,057 (156,107–731,797.9)	7.4 (3.2–15.2)	1.44 (1.39–1.5)
Central Asia	1,049.10104293378 (438.1–2,236.9)	1.5 (0.6–3.3)	2,079.4 (886.9–4,469.5)	2.1 (0.9–4.5)	1.11 (1.05–1.17)
Southeast Asia	32,853.7695118544 (14,429.1–66,791.8)	6.9 (3–14)	92,605.7 (41,149–191,024.1)	12.4 (5.5–25.5)	2.18 (2.09–2.28)
East Asia	48,225.9138354218 (20,818.6–100,143)	3.5 (1.5–7.3)	89,991.4 (39,441.8–185,701.9)	6.4 (2.8–13.2)	2.06 (1.89–2.24)
Africa	20,445.4270504934 (8,874.8–43,350.5)	3.5 (1.5–7.4)	60,192.9 (26,651.9–127,793.2)	4.4 (2–9.4)	0.69 (0.62–0.76)
Central Africa	1,262.21358463437 (537.3–2,655.3)	2.1 (0.9–4.5)	4,768.9 (2,060.6–9,892.1)	2.9 (1.3–6.1)	0.89 (0.75–1.03)
Eastern Africa	4,043.08130172246 (1,726.4–8,543.1)	2.6 (1.1–5.5)	13,730.2 (6,046.1–28,860.7)	3.5 (1.6–7.5)	1.07 (1.01–1.12)
Northern Africa	8,789.0076299909 (3,855.5–18,483.1)	7.4 (3.2–15.6)	20,848.8 (9,244.5–43,732)	9.6 (4.2–20.1)	0.81 (0.79–0.84)
Western Africa	3,395.7592061856 (1,453–7,141.5)	2.1 (0.9–4.5)	13,366.3 (5,760.7–28,368.4)	3.2 (1.4–6.9)	1.09 (0.91–1.26)
North America	39,977.2844158563 (17,689.4–82,640.8)	13.3 (5.9–27.3)	56,162.3 (25,718.6–113,710.7)	16.4 (7.6–33.3)	−0.54 (−1.01 to −0.05)
North Africa and Middle East	22,386.4722474427 (9,812.9–47,102.6)	6.8 (3–14.3)	59,116.3 (26,476.8–125,706.8)	8.8 (3.9–18.7)	0.96 (0.9–1.01)
Southern Sub-Saharan Africa	2,047.42256138018 (881.8–4,435.2)	3.9 (1.7–8.5)	4,157.7 (1,795.1–8,746)	4.8 (2.1–10)	0.64 (0.54–0.74)
Eastern Sub-Saharan Africa	3,903.29284241345 (1,645.1–8,198.6)	2.3 (1–4.9)	12,497.1 (5,380.7–26,310.2)	3 (1.3–6.2)	0.82 (0.79–0.86)
Western Sub-Saharan Africa	3,816.2345113481 (1,629–8,015.4)	2.2 (0.9–4.6)	15,176.8 (6,521.5–32,235.9)	3.3 (1.4–6.9)	1.05 (0.88–1.23)
Central Sub-Saharan Africa	958.262724818009 (406.3–1,975.2)	1.9 (0.8–4.1)	3,861.9 (1,673.7–7,930.5)	2.9 (1.3–6)	1.23 (1.08–1.38)
America	72,424.3224473781 (32,262–149,675.4)	9.8 (4.3–20.2)	116,794.5 (53,755.4–243,772.3)	11.3 (5.2–23.5)	−0.22 (−0.49–0.04)
Tropical Latin America	4,011.06973274821 (1,701.1–8,427.3)	2.5 (1.1–5.3)	6,557.7 (2,825.1–13,831)	2.7 (1.2–5.7)	−0.18 (−0.34 to −0.01)
Central Latin America	20,163.6854279241 (9,003.8–42,043)	12.1 (5.4–25.3)	35,314.1 (15,577.8–73,735.7)	13.2 (5.8–27.5)	−0.12 (−0.27–0.04)
Latin America and Caribbean	30,210.4768160263 (13,328.4–62,616.6)	7.5 (3.3–15.6)	55,162.5 (24,274.2–114,672.1)	8.8 (3.9–18.3)	0.21 (0.09–0.33)
Andean Latin America	4,055.89352141989 (1,797.3–8,748.4)	10.7 (4.8–23)	10,129.2 (4,423.9–21,176.6)	14.4 (6.3–30)	1 (0.92–1.08)
Southern-Latin America	2,627.82901292101 (1,179.4–5,421.3)	5.3 (2.4–11)	5,893.5 (2,574.8–12,247.5)	8.4 (3.7–17.4)	1.44 (1.23–1.65)
United-States-of America	38,051.1197225066 (16,838.4–78,573.6)	14 (6.2–28.8)	53,388.1 (24,521.8–107,618.8)	17.3 (8–34.9)	−0.62 (−1.13 to −0.1)
Region-of-the-Americas	72,424.3224473781 (32,262–149,675.4)	9.8 (4.3–20.2)	116,794.5 (53,755.4–243,772.3)	11.3 (5.2–23.5)	−0.22 (−0.49–0.04)
American Samoa	4.33704784748954 (1.9–9)	8.7 (3.8–18.2)	5.8 (2.5–12.2)	12 (5.2–25.3)	0.92 (0.73–1.1)
Oceania	352.204781576058 (160.6–738.9)	5.4 (2.5–11.3)	1,083.5 (471.3–2,285.6)	7.6 (3.3–15.9)	0.84 (0.67–1.01)
Europe	67,270.7098701165 (30,434–139,695.2)	8.3 (3.8–17.3)	75,944 (34,374.6–158,081.6)	9.8 (4.4–20.5)	0.47 (0.45–0.5)
Eastern Europe	2,097.6940278542 (868.6–4,466.8)	0.9 (0.4–2)	2,323.7 (970–4,890.4)	1.2 (0.5–2.5)	0.95 (0.91–1)
Western Europe	60,788.9195069732 (27,584.9–126,652.6)	15.6 (7.1–32.5)	66,041.5 (29,857.2–136,985.1)	17.3 (7.8–35.9)	0.21 (0.15–0.27)
Central Europe	1,014.73450311381 (425.9–2,088.9)	0.8 (0.3–1.7)	1,032.7 (442.9–2,156.6)	1 (0.4–2)	0.58 (0.53–0.63)

### 3.2 SDI regional level

From 1990 to 2021, significant disparities were observed in ASPR changes across various regions of the world. According to the GBD 2021, the total number of PCOS cases in high SDI regions was estimated at 17 million [95% (UI): 12,981,150.8–23,876,520.6], with an ASPR of 1,720.7 per 100,000 individuals [95% (UI): 1,270.5–2,331.7], followed by middle and high-middle SDI regions. However, over the past three decades, the most significant changes in ASPR have been observed in middle and low-middle SDI regions, with an EAPC of 1.76 [95% (CI): 1.71–1.81] and 1.67 [95% (CI): 1.62–1.71] respectively. In contrast, the ASPR in high SDI regions remained relatively stable, with an EAPC of 0.03 [95% (CI): −0.14–0.2] (Table 1). A similar trend was observed in the ASIR and EAPC of 0.03 [95% (CI): −0.14–0.2] (Table 1). A similar trend was observed in the ASIR and DALYs data, indicating consistent patterns in the changes in the PCOS disease burden across various SDI regions. An analysis encompassing five SDI regions revealed a general positive correlation between the PCOS disease burden and SDI from 1990 to 2021.

### 3.3 GBD regional level

**Figure 1:**
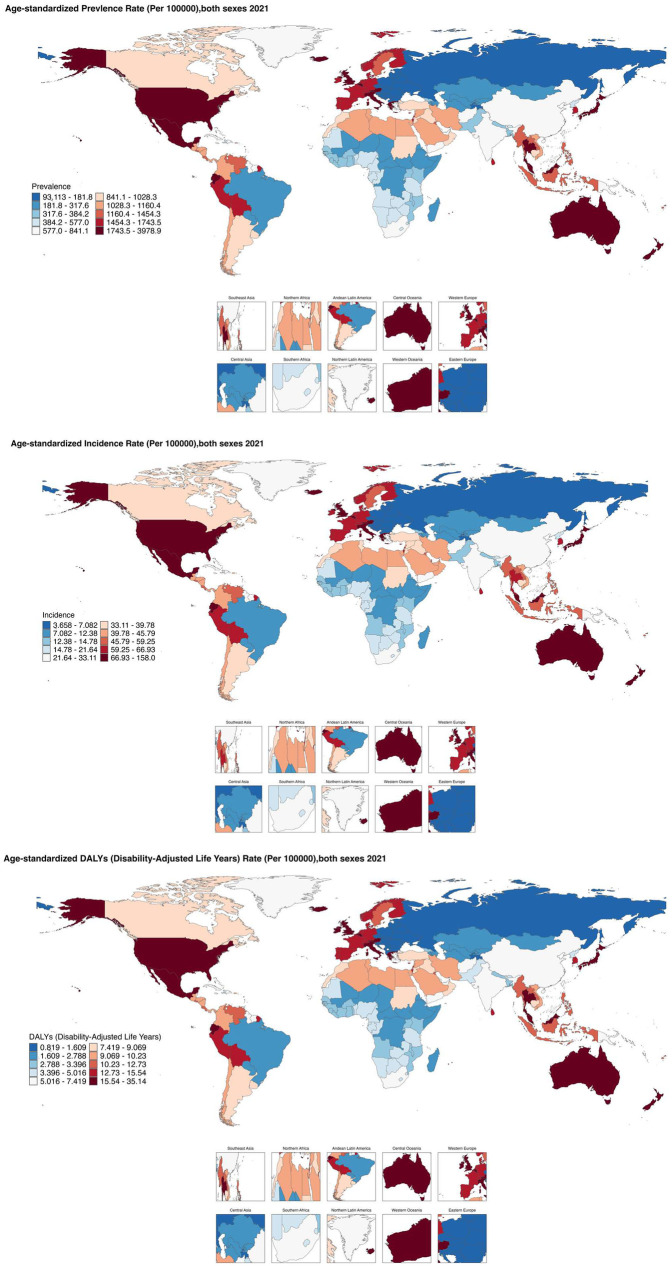
Global regional trends of PCOS ASPR, ASIR and ASR_DALYs in 2021.

**Figure 2:**
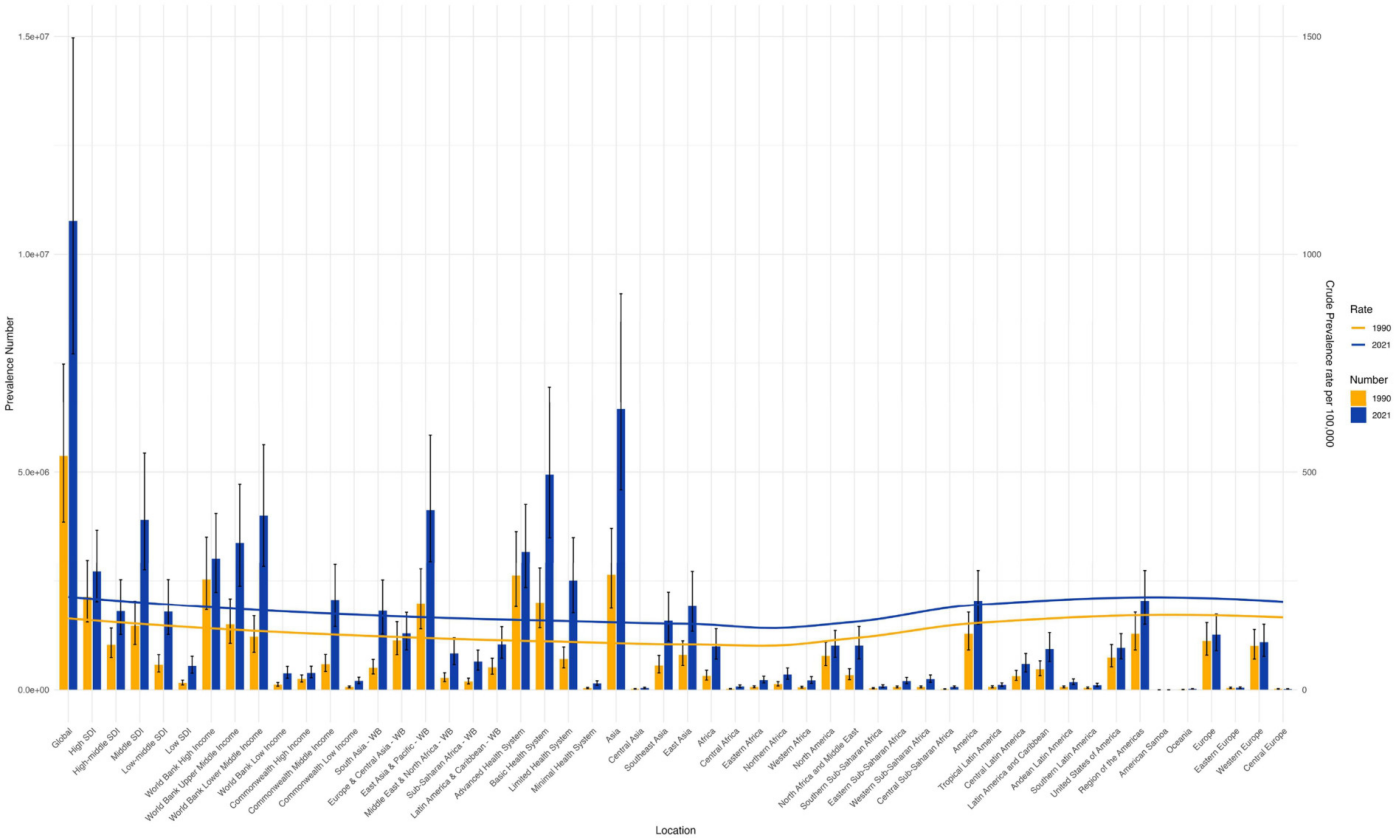
Shows the trends of the number of cases and age-standardized rates (ASRs) of PCOS globally and in each GBD region in 1990 and 2021. (S1 ASIR; S2 ASR_DALYs; S3 ASR_YLDs).

**Figure 3:**
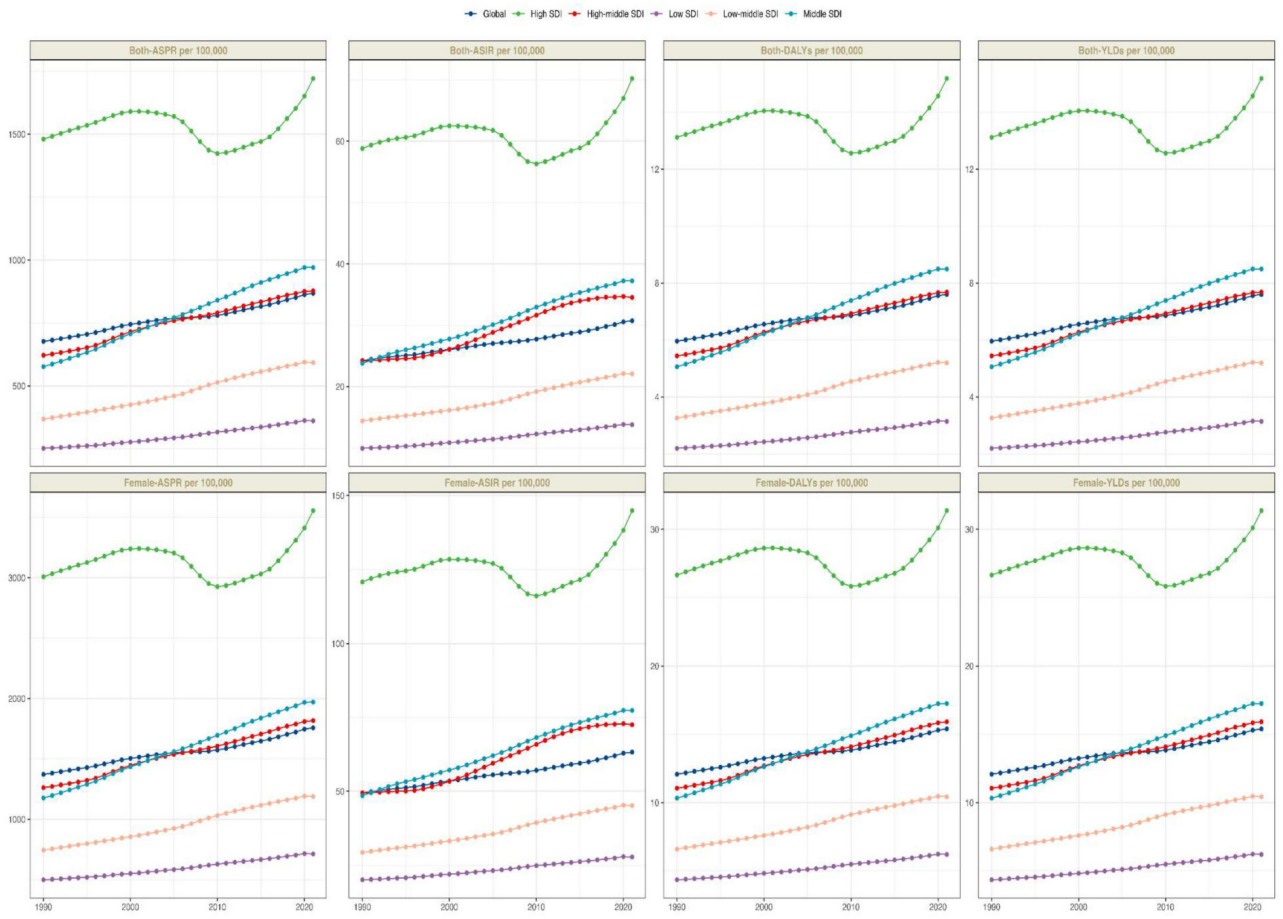
Trends in PCOS from 1990 to 2021 across various SDI regions.

**Figure 4:**
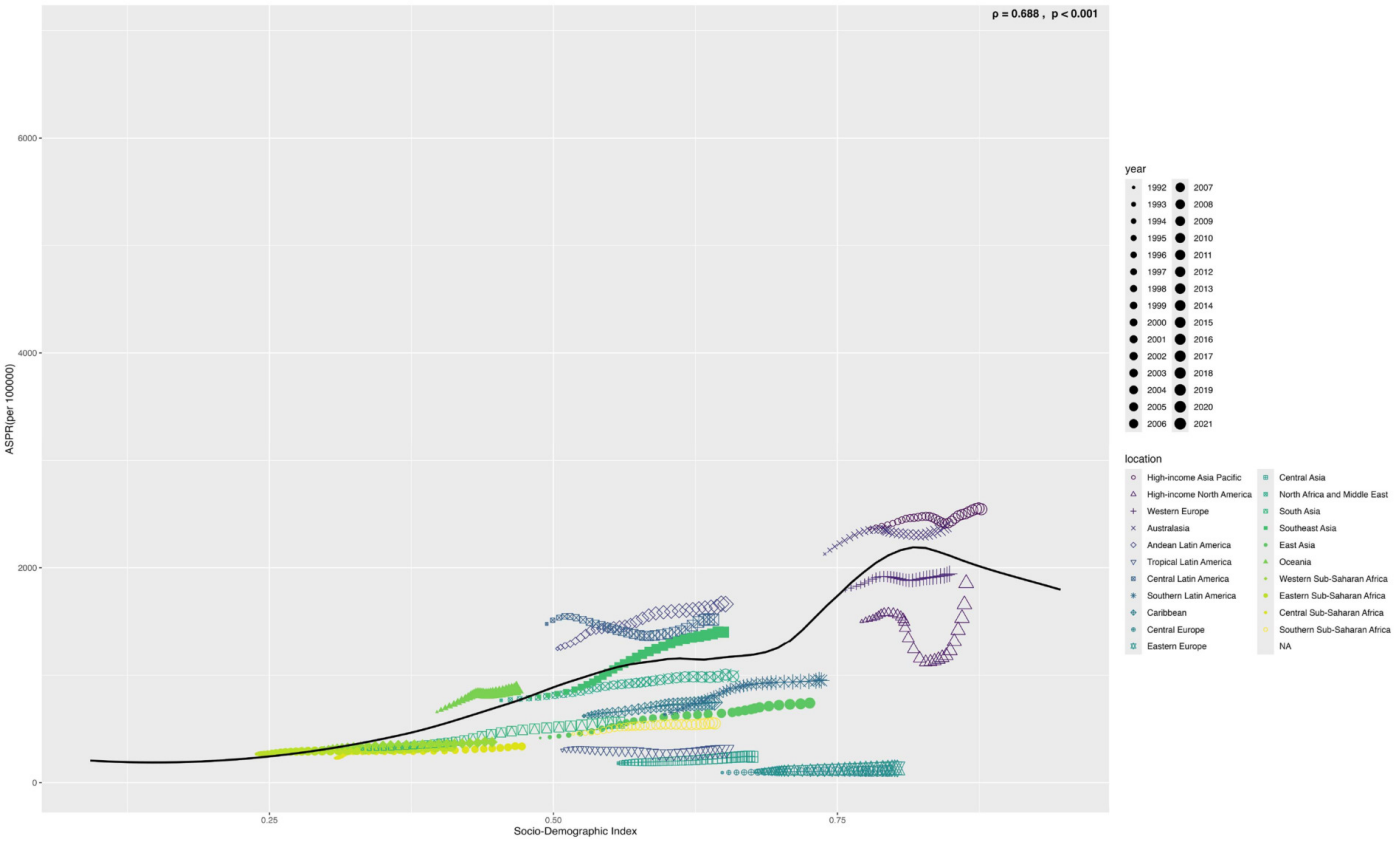
Shows the age-standardized prevalence rate (ASPR) of PCOS in 22 global burden of disease regions (1990–2021) as divided by SDI. (S4 ASIR; S5 ASR_DALYs; S6 ASR_YLDs) (The black line represents the expected value based on the sociodemographic index and the four rates of all locations. 32 points were plotted for each Global Burden of Disease region, and the four age-standardized rates observed in the region from 1990 to 2021 were displayed.).

From 1990 to 2021, the age-standardized prevalence rate (ASPR) of PCOS steadily increased in most regions. In 2021, the highest ASPR was observed in the United States and Western Europe [both ∼1,950 per 100,000, 95% (UI): 1,360–2,730], whereas Central Europe had the lowest [∼112 per 100,000, 95% (UI): 77–159]. The most rapid increases occurred in Southeast Asia and East Asia (EAPC: 2.22 and 2.05, respectively), whereas trends in high-income Asia-Pacific and Western Europe remained relatively stable (EAPC ∼0.2), highlighting marked regional disparities (Figures 1, 2). Across 52 regions, both the age-standardized incidence rate (ASIR) and disability-adjusted life years (ASR_DALYs) for PCOS showed upward trends. Western Europe reported the highest ASIR (75.2 per 100,000), and Central Europe reported the lowest (4.3 per 100,000), with the steepest increases in Southeast and South Asia (EAPC ∼1.9). ASR_DALYs were highest in high-income regions such as Western Europe and the United States (∼17.3 per 100,000), but rose most rapidly in Southeast and East Asia (EAPC ∼2.1). According to the Socio-Demographic Index (SDI), the ASPR was below the expected level in most regions but exceeded expectations in high-income Asia-Pacific, Western Europe, the Andean region of Latin America, Central America, and Oceania. In recent years, the disease burden in East Asia and Western Sub-Saharan Africa has surpassed expectations, whereas Southeast Asia has fallen slightly below the expected levels (Figures 3, 4).

### 3.4 Countries level

**Figure 5:**
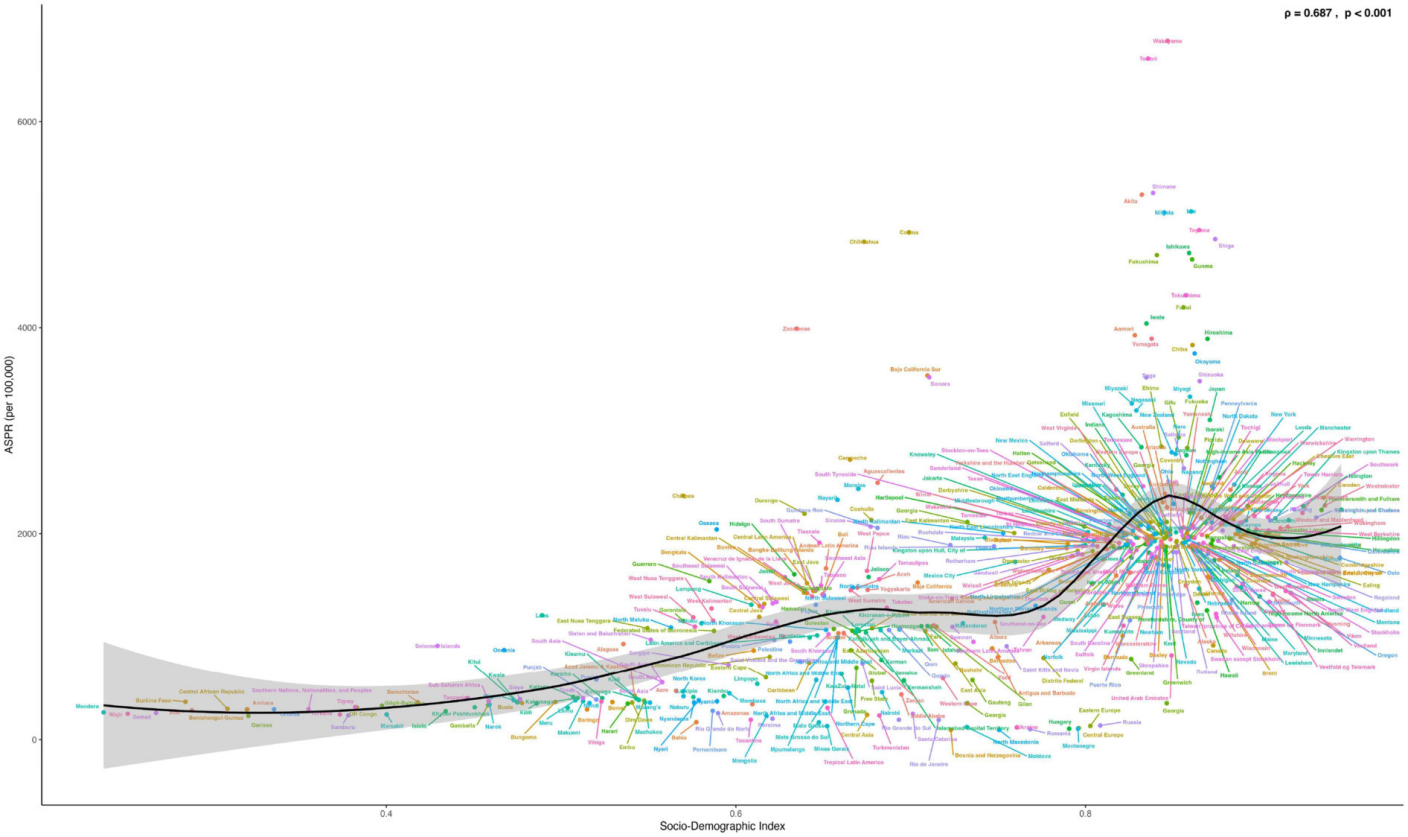
Shows the ASPR of PCOS in 204 countries and regions in 2021 calculated by SDI. (S7 ASIR; S8 ASR_DALYs; S9 ASR_YLDs). The black line represents the expected values of the socio-demographic index and disease incidence rates based on all locations. Each point shows the observed ASPR, ASIR, ASR_DALYs, and ASR_YLDs for each country in 2021. Countries above the black solid line indicate a burden higher than expected (such as Peru and Australia), while those below the line indicate a burden lower than expected (such as Brazil and Canada).

Based on the SDI analysis, as of GBD 2021, the burden of PCOS across various countries exhibited a positive correlation with the level of development. Notably, many developed and certain underdeveloped countries have a disease burden that exceeds expectations. Specifically, in GBD 2021, countries such as India, Western Europe, Mexico, Japan, Australia, and many others experienced a significantly higher burden than expected, whereas countries such as Canada, Central Europe, England, China, Russia, Romania, and others experienced a lower-than-anticipated burden (Figure 5).

### 3.5 Age-SDI patterns

**Figure 6:**
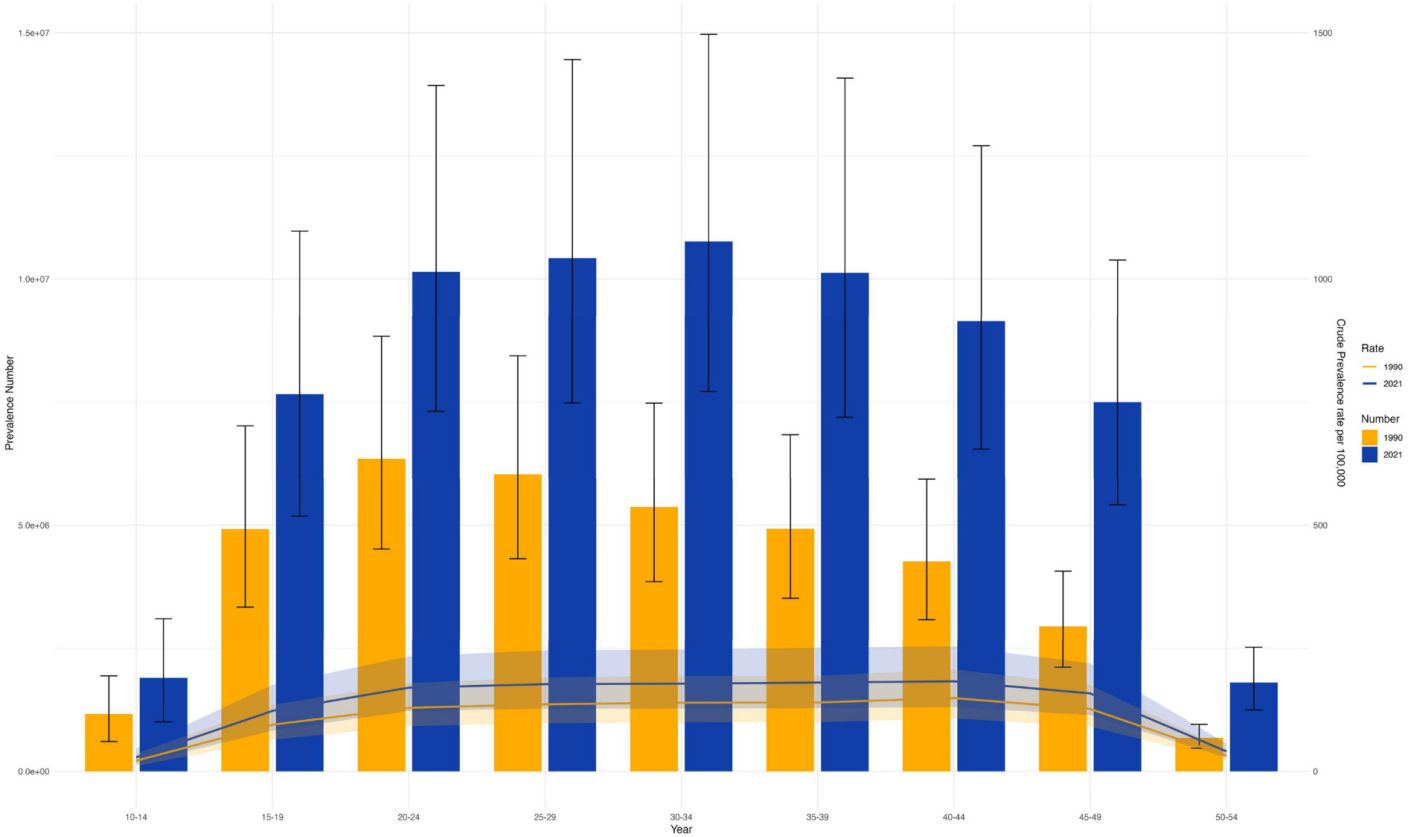
Shows the trends of the number of cases and age-standardized rates (ASRs) of PCOS in different age groups in 1990 and 2021. (S10 ASIR; S11 ASR_DALYs; S12 ASR_YLDs).

The disease burden associated with PCOS was documented for individuals aged 10–54 years, further categorized into nine subgroups. Globally, in 1990 and 2021, the highest incidence was observed among individuals aged 10–14 and 15–19, followed by a notable decline with increasing age. In contrast, peak prevalence occurred in the 20–24 age group in both years (Figure 6 and Supplementary Figures S10–S12). When analyzed by age and SDI, data from 1990 to 2021 showed that in high-SDI regions, case numbers were higher among individuals aged 15–19 than among those aged 10–14, whereas the opposite trend was observed in middle-SDI regions. In 1990, the highest incidence in high-SDI regions was among those aged 25–29, followed by 30–34. By 2021, the 30–34 age group had the highest incidence in high, high-middle, and middle SDI regions, followed by 35–39. Trends of ASR-DALYs and ASR-YLDs were consistent across SDI regions in both 1990 and 2021 (Supplementary Figures S13–S16). Additionally, Supplementary Figure S17 shows that the disease burden for individuals aged 30–34 was comparable across regions in both years. Notably, while ASIR remained relatively stable, other burden indicators exhibited a clear upward trend.

### 3.6 Age-stratified burden gradients of PCOS and comorbid disorders under high BMI exposure: global epidemiological divergence

**Figure 7:**
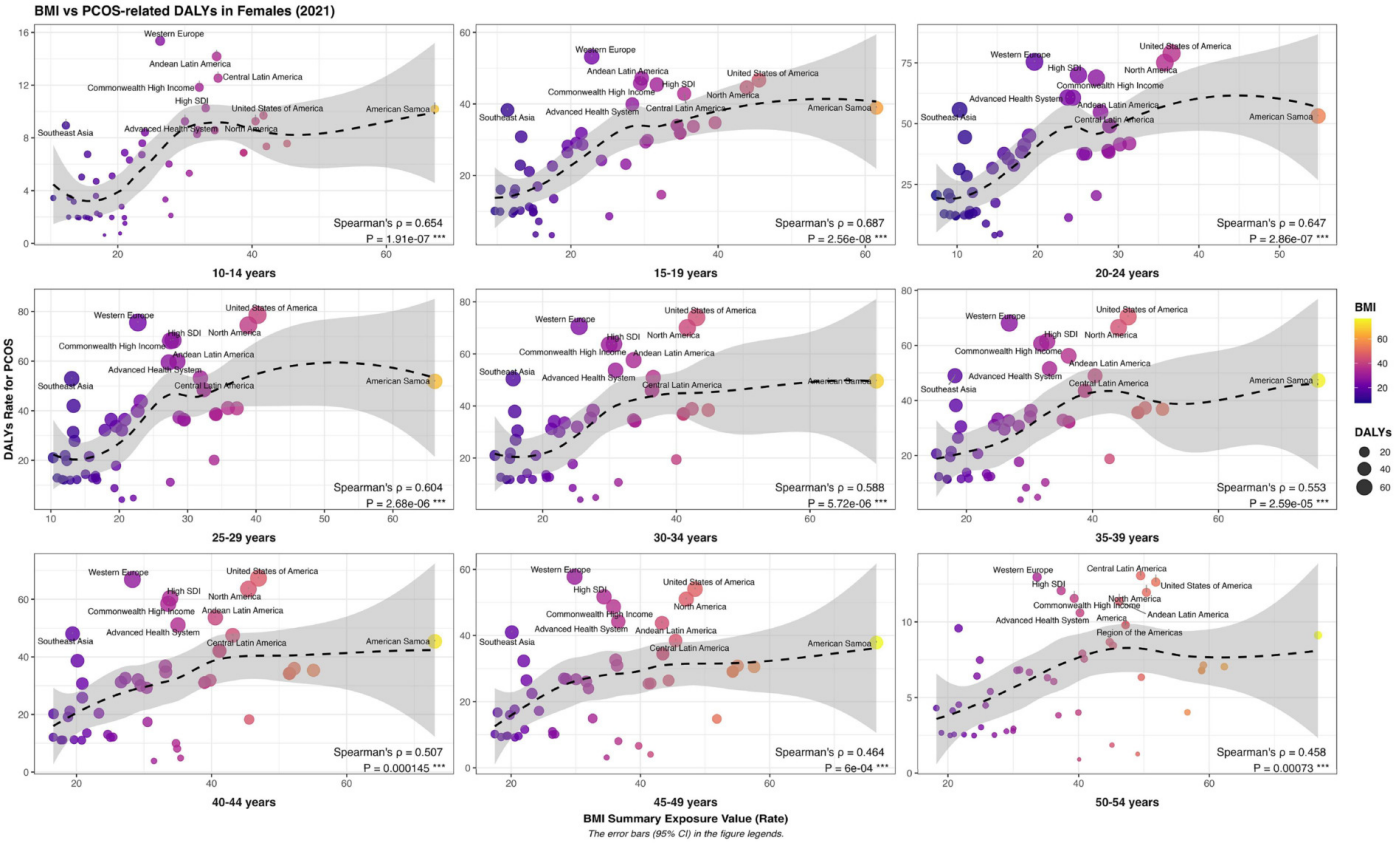
Geographic and Age-stratified variations in the disease burden of polycystic ovary syndrome measured by Age-standardized disability-adjusted life years (DALYs) across female populations under different levels of high body mass Index (BMI) risk exposure.

The disease burden of PCOS showed a typical “inverted U-shaped” distribution trend in different age groups; that is, it first increased and then decreased with age. Specifically, the disease burden of PCOS peaked in the pre-peak period of the reproductive age and then gradually decreased, suggesting that the arrival of the reproductive age is a critical period for the disease. Simultaneously, the risk exposure of a high Body Mass Index (BMI) and the disease burden of PCOS also showed an age correlation. When the exposure level of high BMI was below P_40_, the driving effect on the disease burden of PCOS was most significant, whereas when the exposure level of high BMI was above P_40_, its effect on the disease burden of PCOS gradually weakened. This indicates that high BMI risk exposure does not linearly increase the disease burden of PCOS, suggesting that there may be a threshold effect or interference from other metabolic comorbidity factors. Further regional analysis revealed that in Western European countries, the characteristics of “high PCOS disease burden - low BMI risk exposure” (exposure value <P_40_) were generally presented in all age groups, suggesting that other exposure factors besides BMI risk exposure factors in this region might have played a more important role. Although the disease burden of PCOS in American countries is comparable to that in Western European countries, the risk exposure to high BMI is generally higher, reflecting that high BMI risk exposure in this region may be an important factor driving the aggravation of the disease burden of PCOS. On the whole, developed countries usually show a pattern of “high BMI risk expose-high PCOS disease burden”, suggesting a closer correlation between high BMI risk exposure factors and PCOS disease burden, while developing countries show a “double low” feature of “low BMI risk expose-low PCOS disease burden”. It is indicated that there are other risk factors for the occurrence and development of PCOS (Figure 7).

We analyzed PCOS-related neoplastic diseases, including endometrial and ovarian cancer. In women ≤45 years, the disease burden of endometrial cancer increased with higher BMI risk exposure, indicating a greater sensitivity to BMI in this age group. In women ≥45 years, the disease burden increased when BMI exposure was below P_40_ but decreased above P_40_, suggesting that a high BMI has a smaller impact in this age group. Geographically, the disease burden of endometrial cancer in perimenopausal and menopausal women is mainly concentrated in developed countries, consistent with the trends in PCOS disease burden. Among adolescents and women of reproductive age, the PCOS burden is relatively high in developed regions and shows a gradual increase in some developing countries (Supplementary Figure S18).

We analyzed the burden of PCOS-related psychological disorders in women, including depression and anxiety. In adolescents and reproductive-age women, the burden of depression showed a “rise-then-fall” trend with increasing BMI risk exposure, peaking at BMI P_40–60_, indicating a greater sensitivity to BMI changes in younger women. Among perimenopausal women, the burden fluctuated in a “decrease–increase–decrease” pattern, while in menopausal women, it continued to decline, with overall milder changes, suggesting that BMI's effect of BMI on mental health in this age group is more complex. Geographically, the depression burden in adolescents and reproductive-age women highly overlapped with PCOS in high SDI countries, whereas the overlap was weaker in perimenopausal and menopausal women and showed significant regional differences, reflecting the combined influence of age and cultural background on BMI, mental health, and PCOS (Supplementary Figure S20).

On this basis, we further analyzed anxiety disorders, another common mental disease under high BMI risk exposure. The study found that, except for menopausal women, the disease burden of anxiety disorder in women of all ages showed a trend of “first increasing and then decreasing” with the increase in high BMI risk exposure. The peak of disease burden was mostly concentrated in the high BMI risk exposure value P_30–50_. In contrast, the overall burden of anxiety disorders in menopausal females was relatively low, and the changing trend of risk exposure with a high BMI was not obvious. From the perspective of regional distribution characteristics, the burden of anxiety disorders among adolescents and women of childbearing age is highly consistent with the regional distribution of PCOS, which is mainly concentrated in areas with a high SDI. Among perimenopausal and menopausal female groups, the burden of anxiety disorders also showed a significant upward trend in some developing countries, demonstrating more complex regional heterogeneity (Supplementary Figure S21).

After a comprehensive analysis of the mental health burden, we analyzed the association between metabolic diseases and the disease burden of PCOS. After examining the effect of high BMI risk exposure on the burden of cardiovascular disease and diabetes, we found that the health loss due to cardiovascular disease was significantly higher in postmenopausal women than in premenopausal women. At the regional level, the high-incidence areas of cardiovascular diseases are mainly concentrated in developing countries with middle and low SDI levels, which is significantly different from the overall level and regional distribution of the disease burden caused by PCOS under high BMI risk exposure. Although these two diseases are closely related to high BMI risk exposure, the regional concentration and population sensitivity of the disease burden are different, indicating that the impact of high BMI risk exposure factors on different metabolic systems may be modulated by multiple factors, such as genetic background, endocrine metabolism before and after menopause, diet structure, and lifestyle. Similarly, the disease burden of diabetes under high BMI risk exposure was significantly different from that of PCOS in terms of age distribution trends and geographical distribution patterns. This indicates that although exposure to high BMI risk generation is a common risk factor for metabolic diseases and PCOS, its driving pathways for different types of metabolic diseases may vary significantly and cannot be simply analogized (Supplementary Figure S22).

### 3.7 PCOS disease burden forecast

**Figure 8:**
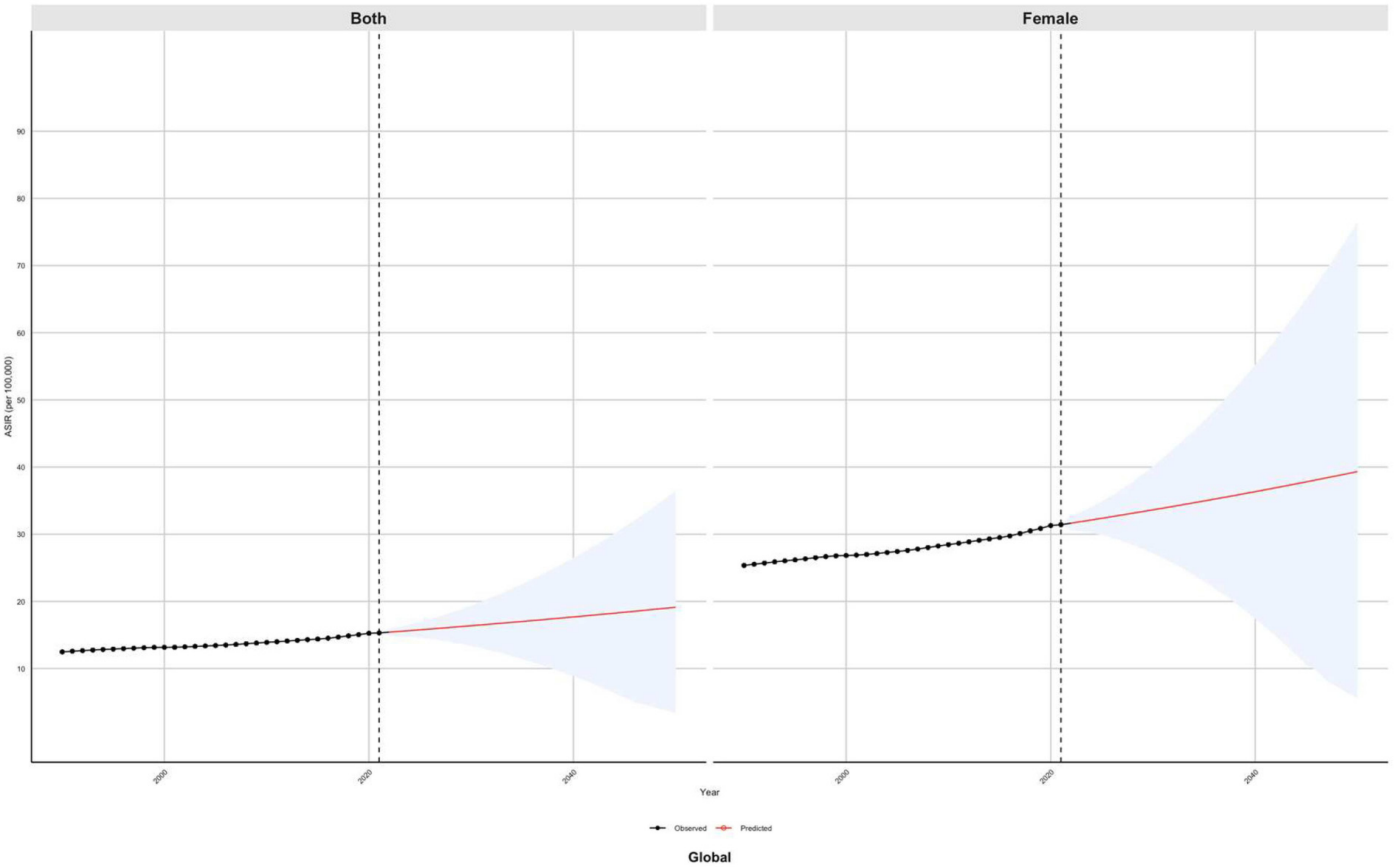
Predicted global trends of the age-standardized incidence rate (ASIR) of PCOS from 2021 to 2050 using the Bayesian age–period–cohort (BAPC) model.

Globally, the ASIR of PCOS is projected to rise from 32 per 100,000 in 2021 to 40 per 100,000 over the next three decades. Additionally, the ASPR is anticipated to increase from 900 per 100,000 to 2,300 per 100,000 population. Furthermore, the ASR-DALYs are expected to escalate from 15 per 100,000 in 2021 to 20 per 100,000. In Southeast Asia, the ASIR is projected to decline from 55 per 100,000 in 2021 to 48 per 100,000. Conversely, the ASPR is anticipated to rise from 2,700 per 100,000 to 2,800 per 100,000 in the same period. Additionally, the ASR-DALYs are expected to increase from 24 per 100,000 to 30 per 100,000. In Southern Africa, the ASIR is projected to increase from 70 per100,000 in 2021 to 150 per 100,000. Similarly, the ASPR is expected to rise from 2,700 per 100,000 to 5,200 per 100,000 in the same period. Additionally, the ASR-DALYs are anticipated to escalate from 23 per 100,000 to 47 per 100,000 individuals. In the Andean Region of Southern Latin America, the ASIR is projected to rise from 45 per 100,000 in 2021 to 55 per 100,000. Concurrently, the ASPR is expected to increase from 3,200 per 100,000 to 3,800 per 100,000 individuals. Furthermore, the age-standardized years of life lost due to premature mortality and DALYs are anticipated to increase from 27 per 100,000 to 33 per 100,000. In regions such as Central Asia, North Africa, Northern Latin America, Central Oceania, Western Oceania, Eastern Europe, and Western Europe, the burden of PCOS is projected to remain relatively stable compared to 2021 levels until 2050. The results obtained from the ARIMA model support the trends identified in the BAPC model, as illustrated in Figures 8 and Supplement Figures S23–S27).

## 4 Discussion

In 2021, the number of new cases of polycystic ovary syndrome (PCOS) worldwide reached 2.3 million, an increase of 48.2% compared with 1990, significantly exceeding the growth rate of the female population of childbearing age during the same period (26.7%), and the cumulative number of patients exceeded 69 million. From 1990 to 2021, the age-standardized prevalence rate and the disability-adjusted annualized rate of life continued to rise at an average annual rate of 0.54% and 0.73% respectively, marking that PCOS has evolved from a single reproductive endocrine disorder to a major public health challenge affecting the entire life cycle of women (26). These trends may be influenced by environmental and behavioral factors. For example, studies have shown that increased bisphenol A exposure may trigger hyperandrogenemia by activating the ovarian P450c17*α* enzyme, thereby increasing the risk of PCOS (27). Global consumption of high-glycemic-index processed foods may also be associated with an increased risk of PCOS (28). Additionally, circadian rhythm disruption, long-term night shift work, accelerated urbanization, air pollution, sedentary behavior, and chronic stress may exacerbate hyperandrogenemia and ovulatory dysfunction by affecting the endocrine and metabolic pathways (29). Overall, these environmental and lifestyle factors may interfere with the environment–endocrine–metabolism axis, creating a potentially high-risk exposure scenario. However, it should be noted that these mechanisms are hypothetical and were not directly analyzed in the GBD 2021 dataset used in this study (30–32).

This study found that middle SDI regions exhibited a unique disease burden pattern: the number of PCOS cases increased most rapidly, while the age-standardized incidence rate (ASIR) rose relatively slowly; however, the ratio of disability-adjusted life years to incidence was significantly higher than the global average. Low-SDI regions had the lowest ASIR, and high-SDI regions showed stable ASIR but persistently high ASR_DALYs. These findings confirm the coexistence of a high complication burden and low diagnosis rate in middle SDI regions (33), suggest that low SDI regions may have underdiagnosis due to insufficient primary care (ultrasound coverage in sub-Saharan Africa ∼18%) (34), and indicate that high SDI regions face insufficient individualized management despite a stable ASIR (35). The regional differentiation of the PCOS disease burden reflects a stage imbalance between medical resource accessibility and the expansion of environmental exposure risk. Middle SDI regions face a dual burden of increasing environmental toxins and lagging medical coverage, leading to abnormal increases in complication burden; low SDI regions experience amplified health loss per case due to gaps in primary care; and high SDI regions are limited by bottlenecks in refined management. Global health governance should therefore shift from homogeneous interventions to an SDI-gradient adaptive strategy: developing regions need resilient environmental risk warning systems and primary care screening, whereas developed regions should implement digital-enabled full-cycle health management. The disease burden intensity ratio should be included in the WHO health equity monitoring system, and resources should be reallocated via policy levers to achieve global equity in women's reproductive and metabolic health.

The manifestation of substantial intracontinental disparities in the PCOS burden underscores the limitations of nationally aggregated health estimates. For instance, in the GBD 2021, the ASIR of PCOS in Southeast Asia was reported to be 53.6 per 100,000 individuals, whereas in Central Asia, this figure was markedly lower at 9.1 per 100,000 individuals. Western Europe is higher than Eastern Europe, North Africa is higher than South Africa, southern Latin America (Andes region) is higher than the north, and Central Oceania is higher than the west. This difference may result from the imbalance in medical access caused by socioeconomic differences and the interference of environmental factors (36–38). Additionally, the rate of lifestyle changes may be a significant factor in this context. Accelerated urbanization and increased consumption of fast food in Southeast Asia could exacerbate insulin resistance through epigenetic mechanisms, such as methylation of the IRS1 gene. Conversely, the traditional dietary pattern characterized by high fiber and low processed fat intake observed in Central Asia may provide a degree of protection against metabolic disorders (39). Notably, variations in the application of diagnostic criteria may further amplify regional disparities. For instance, Western Europe predominantly employs the Rotterdam criteria, whereas 42% of institutions in Eastern Europe continue to use the 1990 NIH criteria. This divergence may result in inconsistencies in phenotype definitions (40, 41). Although this study accounted for several confounding factors by utilizing multi-source data, the inherent limitations of cross-sectional studies, such as ecological fallacy, may still impact causal inference. Future research should explore the molecular mechanisms that contribute to geographic differences in greater detail. This can be achieved through prospective multicenter cohort studies that incorporate environmental exposure, genomics, and metabolomics technologies.

Population-level data indicate that the burden of polycystic ovary syndrome (PCOS) has a nonlinear relationship with the Social Development Index (SDI). Although global SDI growth is accompanied by an increasing burden, some high SDI countries (e.g., Japan and Australia) and low- and middle-income countries (e.g., India and Mexico) have actual burdens significantly above the predictions, whereas Canada, Central Europe, and other regions are below the expected levels. This suggests that the prevalence of PCOS is influenced not only by the availability of medical resources but also by the combined effects of environmental endocrine disruptor exposure, lifestyle transitions, and health policy effectiveness (42, 43). Accordingly, SDI-gradient intervention strategies are recommended: high SDI regions should control daily endocrine disruptors and optimize the management of metabolic complications, while low- and middle-SDI regions should promote low-cost screening tools and strengthen primary care training. Simultaneously, the WHO is establishing a PCOS Global Alliance for Prevention and Control to advance resource-adaptive standardized approaches. In the future, a global dynamic monitoring network should track the spatiotemporal evolution of the disease using heat maps, and resources should be allocated based on the disease burden intensity ratio to achieve equitable governance of women's reproductive and metabolic health.

Significant age-stratified heterogeneity in the global burden of polycystic ovary syndrome (PCOS) has been demonstrated. The highest incidence occurs during adolescence (ages 10–19), while the greatest number of cases is concentrated in the 20–24 age group. Furthermore, in regions with a high SDI, such as Japan and Australia, the peak burden of the disease is postponed until the 30–34 age group. This differential pattern is complementary to the existing evidence chain: the physiological high androgen status in adolescents may lead to overdiagnosis, while the delayed diagnosis caused by hidden symptoms may aggravate metabolic disorders (44), and the aging trend in high SDI areas may be associated with the risk of delayed birth and exposure to environmental endocrine disruptors (45). Our study also confirmed the need to implement differentiated prevention and control strategies at the public health level and promote school-based screening strategies for adolescents (46–48). We suggest that for women of childbearing age in high SDI areas, resources from gynecology, endocrinology, and workplace health management should be integrated to build an interdisciplinary complication prevention and control network. The SDI for low- and middle-regions is a priority universal low-cost diagnostic tool and stepwise management solution. In the future, a global heat map of PCOS diseases needs to be constructed to dynamically identify regional age burden peaks and allocate resources in a targeted manner, ultimately achieving cross-age reduction of preventable burden.

This study found that from 1990 to 2021, the global age-standardized prevalence rate (ASPR) of PCOS increased significantly, showing a “development paradox” wherein low- and middle-SDI regions grew faster than developed regions. The high incidence in adolescent females (15–19 years) coexists with a high disease burden in women of reproductive age (30–35 years), likely due to the combined effects of improved diagnostic capacity and the obesity epidemic in developing countries (49, 50), while stable burdens in developed countries suggest that treatment systems are approaching saturation (51, 52). High BMI risk exposure exhibits a life-cycle effect on PCOS and related comorbidities: young women (<40 years) are most affected at low BMI thresholds (<P_40_), with the impact declining with age, consistent with age-dependent decreases in estrogen receptor *α* expression in adipose tissue (53). Endometrial cancer risk in reproductive-age women shows a linear effect, plateauing postmenopause (54, 55), while ovarian cancer displays an inverted U-shaped pattern (BMI P_40–60_ peak), indicating a critical metabolic stress threshold in the perimenopausal ovaries (56, 57). Mental comorbidities (depression/anxiety) overlap geographically with PCOS (especially in high SDI countries among young and middle-aged women), supporting hypothalamic-pituitary-adrenal axis dysfunction as a shared pathway (58). Mental comorbidities (depression/anxiety) overlap geographically with PCOS (especially in high SDI countries among young and middle-aged women), supporting hypothalamic-pituitary-adrenal axis dysfunction as a shared pathway (59, 60). Therefore, BMI risk management should be strengthened in adolescent females to reduce PCOS and psychiatric comorbidity risks; reproductive-age women should be included in endometrial cancer risk-stratified monitoring; and developing countries should simplify PCOS diagnosis and treatment pathways while integrating obesity management. Future research should focus on the molecular mechanisms underlying the effects of BMI thresholds.

Finally, we integrated data on global subregional heterogeneity with interdisciplinary predictive models (BAPC/ARIMA) for the first time. The results showed that the age-standardized incidence rate (ASIR) in Southeast Asia decreased (55→48/100,000), but the age-standardized prevalence rate increased (2,700→2,800/100,000). In South Africa, the ASIR (70→150/100 000) and disability-adjusted life years (ASR_DALYs 23→47/100 000) increased dramatically. In the Andes region of Latin America, the ASIR showed a moderate increase (45→55 per 100,000). This finding forms a triple mutual evidence with the leading research: the Southeast Asia paradox confirms that the mechanism of obesity prevention and control policies in the social transition period can reduce the incidence of obesity in the short term, but behavioral risk factors such as sedentary lifestyle promote the chronicity process (61). In contrast, the trajectories observed in South Africa appear to reflect the dynamics described by the environment–metabolism imbalance cycle (62). Notably, the “moderate upward” trend of ASIR in southern Latin America (the Andes region) may suggest protective mutations in the indigenous genome, alleviating the metabolic interference of environmental endocrine disruptors (63). This model provides a scientific basis for countries to formulate the strategy of “early warning + precise prevention and control” and provides a new idea for targeted population genetic characteristics of precise prevention, marking the governance of PCOS in the era of customization.

## 5 Conclusion

Based on a systematic assessment of the global disease burden of PCOS, this study revealed significant heterogeneity in the disease burden related to high BMI risk exposure in women at the life cycle evolution and regional levels. The disease burden of PCOS gradually decreased with age, showing the most significant response in patients with a high BMI and low exposure intervals. In developed countries, it shows a differentiation pattern of “high burden-high BMI and low exposure” (e.g., Western Europe) and “high burden-high BMI and high exposure” (e.g., the United States). The response of cancer burden related to PCOS was disease-specific, showing that endometrial cancer increased with an increase in exposure dose in young women and tended to be flat in middle-aged and elderly women. Ovarian cancer showed an inverted U-shaped curve, with a peak disease burden in perimenopausal women. The disease burden of mental disorders (depression/anxiety) and PCOS showed a significant spatial synergistic pattern in young women and countries and regions with high SDI, and most were concentrated in the middle BMI risk exposure interval. Metabolic diseases (such as cardiovascular disease and diabetes) in postmenopausal women show a trend of shifting the disease burden to developing countries, suggesting that the driving mechanisms of metabolic diseases are evolutionarily differentiated from those of PCOS. These findings highlight the importance of constructing hierarchical intervention strategies based on life cycle stage and regional exposure characteristics to effectively block the risk of the reproductive-psycho-metabolic cascade.

## 6 Limitations and innovations of the study

The cross-regional comparability of high BMI exposure and insufficient confounding control constitute core challenges. First, the regional heterogeneity of the global BMI measurement standards led to exposure classification bias. Second, there are obvious differences in interfering factors in the studies of PCOS complications in different regions: the widespread use of hypoglycemic drugs in developed countries may mask the true association between obesity and complications, while the widespread problems of malnutrition and infection in poverty-stricken areas will weaken the apparent impact of obesity on health hazards. Third, the GBD does not include data on visceral fat distribution and cannot quantify the independent contribution of central obesity to complications. Finally, the regional gradient of medical accessibility and diagnostic capabilities leads to a systematic underestimation of the burden in areas with a low SDI. In addition, we have developed innovative analysis tools that can simultaneously track global trends, regional environmental changes, and personal health data. These tools can accurately identify high-risk areas. However, this study had some limitations. Incomplete data observed in some countries (for example, not fully reflecting the improvement in the health of the wealthy population in South Africa) may affect the accuracy of our predictions.

## Data Availability

The original contributions presented in the study are included in the article/Supplementary Material, further inquiries can be directed to the corresponding authors.
