# Supplementary material for: The evolution of the global disease burden of polycystic ovary syndrome and the role of regional heterogeneity in high body mass index exposure: a spatiotemporal analysis based on the global burden of disease 2021
Source: Front Reprod Health. 2025 Nov 4;7:1600995. doi: 10.3389/frph.2025.1600995 (PMC12623367; doi:10.3389/frph.2025.1600995)
Supplement: Supplementary file 1 [file Table1.docx]

| *Location* | Incidence | | | | | |
| --- | --- | --- | --- | --- | --- | --- |
|  | 1990 | | 2021 | | | EAPC_CI |
|  | Numbers(95%UI) | Age-standardized rate  (per100000)(95%UI) | Numbers(95%UI) | Age-standardized rate  (per100000)(95%UI). | |  |
| *Global* | *1476225.3(1057983.5-2045276.9)* | *24.2 (17.4-33.5)* | *2301505.6 (1655989.2-3167177.8)* | *30.7 (22.1-42.4)* | | *0.73 (0.71 to 0.76)* |
| *High SDI* | *437301.5 (319330.7-606631.9)* | *58.8 (43-81.4)* | *494212.2 (367071.5-670948)* | *70.2 (52-95.3)* | | *0.1 (-0.08 to 0.28)* |
| *High-middle SDI* | *261030.5 (187118.7-358760.3)* | *24.2 (17.3-33.5)* | *305810 (216061.4-424907.9)* | *34.5 (24.3-48.3)* | | *1.45 (1.36 to 1.54)* |
| *Middle SDI* | *505653.9 (359588-703114.3)* | *23.8 (16.9-33.1)* | *812669 (572963.7-1126099.7)* | *37.2 (26.2-51.8)* | | *1.52 (1.48 to 1.55)* |
| *Low-middle SDI* | *209333.5 (148000.1-293837.3)* | *14.4 (10.4-20)* | *481689 (338285.7-670310.3)* | *22.1 (15.5-30.7)* | | *1.5 (1.44 to 1.55)* |
| *Low SDI* | *61881.6 (43355.6-87726)* | *9.9 (7.2-13.9)* | *205458.3 (144223.9-291379.8)* | *13.8 (9.8-19.3)* | | *1.14 (1.11 to 1.17)* |
| *World-Bank-High Income* | *541762.2 (396058.4-744374.6)* | *61.1 (44.5-84.3)* | *577079 (426601.2-781420.4)* | *71.2 (52.5-96.1)* | | *0.11 (-0.04 to 0.26)* |
| *World-Bank-Upper-Middle Income* | *463803.1 (332049.6-640647.5)* | *20.4 (14.4-28.4)* | *603552.4 (425593.2-838856.1)* | *31.3 (22.1-43.7)* | | *1.41 (1.35 to 1.47)* |
| *World-Bank-Lower-Middle Income* | *422468.2 (299183.9-590008.8)* | *17.1 (12.3-23.8)* | *975836 (690009.2-1352544.3)* | *25.7 (18.2-35.7)* | | *1.48 (1.42 to 1.53)* |
| *World-Bank Low Income* | *47156.8 (33104-66751.5)* | *12 (8.6-16.7)* | *143359.8 (100369.6-202455.9)* | *15.4 (11-21.7)* | | *0.89 (0.87 to 0.91)* |
| *Commonwealth High Income* | *49458.5 (35917.4-66965.6)* | *54.9 (39.9-74.5)* | *68169.1 (48878.6-95023.6)* | *68.9 (49.3-96.3)* | | *0.56 (0.45 to 0.66)* |
| *Commonwealth-Middle-Income* | *198460.5 (141926.1-276702.6)* | *13.8 (10-19.1)* | *512268.7 (365368.6-709122.7)* | *21.3 (15.1-29.5)* | | *1.56 (1.48 to 1.64)* |
| *Commonwealth Low Income* | *25801.3 (17924.7-36698.2)* | *9.5 (6.7-13.4)* | *66869.2 (47321.7-94457.9)* | *13.6 (9.7-19.1)* | | *1.29 (1.22 to 1.35)* |
| *South Asia- WB* | *172469.3 (123555.6-240042.5)* | *12.6 (9.1-17.4)* | *439909.5 (315913.9-605360.4)* | *20.8 (14.8-28.7)* | | *1.86 (1.73 to 1.99)* |
| *Europe&Central Asia - WB* | *240880.2 (172360.6-331955)* | *32.3 (23-44.8)* | *245638.9 (174383.4-339550.2)* | *39 (27.6-54.2)* | | *0.88 (0.79 to 0.98)* |
| *East-Asia& Pacific - WB* | *561276.6 (402130.6-778408.4)* | *25.8 (18.4-35.7)* | *745314.1 (538524.5-1032153.1)* | *42.4 (30.6-59.1)* | | *1.77 (1.72 to 1.83)* |
| *Middle East & North Africa - WB* | *104300.6 (71982.3-148482.6)* | *30.8 (21.5-43.5)* | *191408.6 (134800.9-272006)* | *39.5 (27.9-56.2)* | | *0.9 (0.81 to 0.98)* |
| *Sub-Saharan Africa - WB* | *74115.1 (51917.3-105312.5)* | *11.3 (8.1-15.9)* | *231557.7 (162753.7-327991.2)* | *14.7 (10.5-20.7)* | | *0.76 (0.7 to 0.83)* |
| *Latin America & Caribbean - WB* | *187978.5 (130497.3-267918.2)* | *33.7 (23.4-47.9)* | *242534.7 (170902.2-339080.7)* | *40 (28.2-56.1)* | | *0.32 (0.22 to 0.41)* |
| *Advanced Health System* | *567808.6 (414557.7-781167.8)* | *49.1 (35.8-67.9)* | *608234 (449376.3-823362.3)* | *59.7 (43.9-80.6)* | | *0.42 (0.31 to 0.52)* |
| *Basic Health System* | *640190.4 (455028.5-889468.9)* | *23.1 (16.4-32.2)* | *961899.9 (678292-1340880.5)* | *37.3 (26.2-52.1)* | | *1.6 (1.55 to 1.66)* |
| *Limited Health System* | *251237.2 (177968.6-352944.5)* | *12.9 (9.4-18)* | *669132.2 (477165.3-930045.3)* | *19.7 (14-27.3)* | | *1.52 (1.45 to 1.59)* |
| *Minimal Health System* | *15964.9 (11131.5-22579.7)* | *9.4 (6.7-13.1)* | *60572.4 (42401.7-85645.5)* | *13.3 (9.4-18.7)* | | *1.17 (1.1 to 1.24)* |
| *Asia* | *793636 (568761.9-1097309.8)* | *21 (15.1-29)* | *1294489.7 (927249.6-1790026.9)* | *30.7 (21.9-42.5)* | | *1.36 (1.32 to 1.4)* |
| *Central Asia* | *5347.8 (3686.1-7702.4)* | *6.8 (4.7-9.7)* | *8122.3 (5654.1-11355.5)* | *9.1 (6.3-12.7)* | | *1.05 (0.99 to 1.12)* |
| *Southeast Asia* | *174333.2 (122499-243431.4)* | *29.3 (20.6-40.8)* | *357262.3 (254498.6-495461.4)* | *53.6 (37.9-74.9)* | | *2.24 (2.14 to 2.35)* |
| *East Asia* | *218485.8 (154343.7-301132.9)* | *15.6 (11.1-21.8)* | *268458.5 (190157.1-373877.4)* | *27.3 (19.2-38.4)* | | *1.79 (1.64 to 1.95)* |
| *Africa* | *122302.2 (85897.2-174431.5)* | *15 (10.7-21.2)* | *319908.8 (224226.3-449944.6)* | *17.8 (12.6-25)* | | *0.43 (0.36 to 0.51)* |
| *Central Africa* | *7931.2 (5511.5-11340.2)* | *9.5 (6.8-13.4)* | *29218 (20173.9-41544.2)* | *13 (9.2-18.3)* | | *0.91 (0.77 to 1.04)* |
| *Eastern Africa* | *26106.6 (18300.8-37162.2)* | *11.4 (8.2-15.9)* | *79974.7 (56147.7-113877.5)* | *15.1 (10.7-21.2)* | | *0.97 (0.91 to 1.03)* |
| *Northern Africa* | *48437 (33514.5-69161.3)* | *30.8 (21.5-44)* | *89112.6 (62107.6-127629.8)* | *40.2 (28.3-57.7)* | | *0.84 (0.81 to 0.87)* |
| *Western Africa* | *22102.2 (15436.6-31453.4)* | *10.3 (7.4-14.3)* | *81827.7 (57545.3-116614.9)* | *14.1 (10-19.8)* | | *0.79 (0.65 to 0.94)* |
| *North America* | *134114.4 (94942.4-187145.5)* | *58.1 (40.9-81.3)* | *203410 (150001.7-271131.6)* | *73.2 (54.4-96.6)* | | *-0.55 (-1.03 to -0.06)* |
| *North Africa and Middle East* | *128593.5 (88937.3-183308.6)* | *28.7 (20.1-40.6)* | *242427.1 (171058.1-342784.6)* | *37.3 (26.3-52.8)* | | *0.92 (0.88 to 0.96)* |
| *Southern Sub-Saharan Africa* | *11799.7 (8261-16787.8)* | *17.2 (12.1-24.3)* | *18377.8 (12888.4-26038)* | *21 (14.7-29.8)* | | *0.63 (0.54 to 0.72)* |
| *Eastern Sub-Saharan Africa* | *25902 (18214-36986.2)* | *10.4 (7.5-14.6)* | *76605.1 (53945.1-108540.5)* | *13.1 (9.3-18.4)* | | *0.8 (0.76 to 0.83)* |
| *Western Sub-Saharan Africa* | *24710.3 (17219.7-35159.5)* | *10.3 (7.4-14.4)* | *92826.2 (65226.5-132560.9)* | *14.2 (10.1-20)* | | *0.79 (0.64 to 0.93)* |
| *Central Sub-Saharan Africa* | *6063 (4247.4-8685.3)* | *8.8 (6.3-12.4)* | *23871.9 (16637.7-33976)* | *13 (9.2-18.4)* | | *1.21 (1.06 to 1.36)* |
| *America* | *320708.9 (225689.3-448079.9)* | *40.9 (28.8-57.2)* | *444934.9 (330613.1-592182.4)* | *50.4 (37.3-67.5)* | | *0.01 (-0.23 to 0.25)* |
| *Tropical Latin America* | *22284.6 (15199.8-32004)* | *11.7 (8-16.7)* | *23769.1 (16611.4-32960.1)* | *12.3 (8.4-17.2)* | | *-0.27 (-0.44 to -0.1)* |
| *Central Latin America* | *119114.9 (81948.2-168844.6)* | *53.7 (37-75.9)* | *143013.7 (100629.4-200290.7)* | *57.2 (40-80)* | | *-0.17 (-0.34 to 0)* |
| *Latin America and Caribbean* | *175877 (121576.9-250897.8)* | *34.7 (24.1-49.4)* | *221379.9 (155474.9-309629)* | *40.4 (28.3-56.6)* | | *0.22 (0.11 to 0.34)* |
| *Andean Latin America* | *25250.9 (17294.5-36023.1)* | *50 (34.4-71)* | *42634.6 (29473.4-60429.4)* | *64.7 (44.7-91.9)* | | *0.79 (0.69 to 0.89)* |
| *Southern-Latin America* | *12429.8 (8602.2-17727.2)* | *23.3 (16.1-33.3)* | *21574.4 (15329.6-30970)* | *36.6 (25.9-52.4)* | | *1.42 (1.22 to 1.63)* |
| *United-States-of America* | *127848.5 (90504-178310.7)* | *61.3 (43.2-85.9)* | *194555.5 (142842.7-258038.3)* | *76.8 (57.1-100.9)* | | *-0.64 (-1.15 to -0.12)* |
| *Region-of-the-Americas* | *320708.9 (225689.3-448079.9)* | *40.9 (28.8-57.2)* | *444934.9 (330613.1-592182.4)* | *50.4 (37.3-67.5)* | | *0.01 (-0.23 to 0.25)* |
| *American Samoa* | *22 (15.5-30.8)* | *37.2 (26.2-52.2)* | *31.4 (21.7-45.9)* | *52 (36.2-75.8)* | | *0.96 (0.76 to 1.16)* |
| *Oceania* | *2013.1 (1401.2-2847)* | *23.8 (16.7-33.5)* | *5300.9 (3692.1-7439.8)* | *32.9 (23-46.3)* | | *0.81 (0.66 to 0.96)* |
| *Europe* | *237069.8 (169674.3-326634.9)* | *34.5 (24.5-47.9)* | *239357.7 (169918.3-330720.8)* | *42.9 (30.4-59.5)* | | *0.98 (0.88 to 1.07)* |
| *Eastern Europe* | *8328.3 (5913.3-11639.2)* | *4.2 (3-5.9)* | *7596.1 (5457.5-10592.6)* | *5.3 (3.7-7.5)* | | *0.89 (0.85 to 0.93)* |
| *Western Europe* | *206740.9 (146330.9-286172.1)* | *69.9 (49.2-97.8)* | *206192.2 (145653.5-286689.1)* | *75.2 (53.1-105)* | | *0.14 (0.1 to 0.18)* |
| *Central Europe* | *4244.9 (2905.8-6223.9)* | *3.6 (2.5-5.3)* | *3175.5 (2221.1-4441)* | *4.3 (3-6.1)* | | *0.53 (0.46 to 0.59)* |
| *location* | Prevalence | | | | | |
|  | 1990 | | 2021 | | | EAPC_CI |
|  | Numbers(95%UI) | Age-standardized rate  (per100000)(95%UI) | Numbers(95%UI) | | Age-standardized rate  (per100000)(95%UI). |  |
| *Global* | *36651157.2(26227943.2-50603929.8)* | *676.8 (485.5-932.6)* | *69473252.4 (49531420-95724479.2)* | | *867.7 (618.7-1195.3)* | *0.75 (0.71 to 0.78)* |
| *High SDI* | *13783058.5 (10021809.4-19223038.3)* | *1479.8 (1075.7-2053.4)* | *17573919.8 (12981150.8-23876520.6)* | | *1720.7 (1270.5-2331.7)* | *0.03 (-0.14 to 0.2)* |
| *High-middle SDI* | *7047030 (4981606.3-9727416.6)* | *621.7 (439.3-859.5)* | *11180894.9 (7874794.9-15590881.4)* | | *877.5 (616.7-1221.5)* | *1.16 (1.11 to 1.21)* |
| *Middle SDI* | *10580143.2 (7410905.3-14674450.8)* | *576.7 (404.4-798.9)* | *24613369.9 (17452531.6-34074253.7)* | | *970.2 (687.2-1340.9)* | *1.76 (1.71 to 1.81)* |
| *Low-middle SDI* | *4094311.3 (2868780.9-5765430.7)* | *368.3 (260.4-514.5)* | *12118405.4 (8434181-17027257.3)* | | *592.4 (413-831.8)* | *1.67 (1.62 to 1.71)* |
| *Low SDI* | *1121931 (784807-1611458.4)* | *252.4 (178.4-359.2)* | *3938251.3 (2745129.9-5589751.2)* | | *361.6 (255.3-509.8)* | *1.24 (1.21 to 1.28)* |
| *World-Bank-High Income* | *16748910.2 (12163633.3-22944490.4)* | *1558.1 (1131.3-2128.3)* | *20132268.6 (14827038.9-27314275.5)* | | *1763.5 (1300.4-2396.9)* | *-0.01 (-0.16 to 0.14)* |
| *World-Bank-Upper-Middle Income* | *10574147.8 (7408942.4-14750935.7)* | *485.9 (340.5-676.8)* | *20345626.5 (14332447.6-28213302)* | | *812.6 (570.8-1123.3)* | *1.64 (1.55 to 1.74)* |
| *World-Bank-Lower-Middle Income* | *8440600.8 (5965275.5-11808115.2)* | *434.9 (307.7-606.4)* | *26163393 (18272756.9-36695088.6)* | | *716.5 (501.1-1005.3)* | *1.85 (1.77 to 1.93)* |
| *World-Bank Low Income* | *862591.4 (600137.6-1229503.7)* | *315.3 (221.7-446.3)* | *2783244.8 (1917591.7-3959813.4)* | | *416.5 (287.9-587.3)* | *0.96 (0.94 to 0.99)* |
| *Commonwealth High Income* | *1607542.9 (1166394.2-2225879.2)* | *1385.5 (1002-1920.4)* | *2378784 (1706949.9-3311197.9)* | | *1752.5 (1257.7-2428.2)* | *0.56 (0.46 to 0.67)* |
| *Commonwealth-Middle-Income* | *4046031.8 (2880612.3-5648186.5)* | *351.7 (252.6-489.4)* | *13473435.8 (9409885.7-18889846.4)* | | *595.4 (416.6-833.7)* | *1.92 (1.84 to 1.99)* |
| *Commonwealth Low Income* | *448535.3 (311553.3-649696.5)* | *235.6 (164.5-337.4)* | *1404409.1 (973640.1-1990177.4)* | | *352 (244.3-497.7)* | *1.42 (1.36 to 1.47)* |
| *South Asia- WB* | *3511154.2 (2504013.6-4894814.3)* | *321.5 (230.9-446.3)* | *11848596.9 (8333792.5-16574375.3)* | | *570.5 (401.4-797)* | *2.15 (2.04 to 2.27)* |
| *Europe&Central Asia - WB* | *7614157.3 (5371668.9-10561179.5)* | *890.8 (628-1235.1)* | *8761385.4 (6157123.2-12324344.8)* | | *1024.5 (717.6-1443.8)* | *0.38 (0.35 to 0.41)* |
| *East-Asia& Pacific - WB* | *13980093.9 (10018861.1-19417295.3)* | *691.5 (497.2-961.3)* | *25534892.8 (18404255-35803825.3)* | | *1093.8 (782.4-1533.4)* | *1.61 (1.55 to 1.66)* |
| *Middle East & North Africa - WB* | *1987856.5 (1387094.8-2815222.8)* | *806.9 (563.6-1142.9)* | *5367433.4 (3762038.5-7603740.7)* | | *1032.4 (723.5-1463.6)* | *0.9 (0.82 to 0.99)* |
| *Sub-Saharan Africa - WB* | *1340479.3 (935579-1925004.9)* | *290.6 (204.6-414.4)* | *4539775.9 (3134250.4-6466291.6)* | | *397.7 (278.4-563.1)* | *0.93 (0.86 to 1.01)* |
| *Latin America & Caribbean - WB* | *3721323.6 (2562708.7-5210753.5)* | *827.7 (570.9-1156.3)* | *7008084.4 (4942278.9-9743064.9)* | | *1007.2 (709.2-1399.4)* | *0.34 (0.23 to 0.44)* |
| *Advanced Health System* | *17349361.4 (12584994.4-23781916.8)* | *1271.4 (921.5-1738.6)* | *21154221.8 (15573659.3-28771671.3)* | | *1484.3 (1096.8-2026.7)* | *0.13 (-0.01 to 0.27)* |
| *Basic Health System* | *14066187.7 (9900136.1-19602002)* | *568.1 (400.2-790.6)* | *30516465.6 (21616756.1-42543674.2)* | | *959.8 (677.4-1335.9)* | *1.75 (1.67 to 1.83)* |
| *Limited Health System* | *4925706.2 (3482413.5-6925214.7)* | *328.3 (234.8-459)* | *16662344.4 (11625899.1-23359835.5)* | | *547.7 (382.8-765.4)* | *1.87 (1.79 to 1.94)* |
| *Minimal Health System* | *285218.6 (199008.7-413707.9)* | *244.7 (172-350.8)* | *1091809.4 (756623.3-1554184.5)* | | *347.7 (242.2-492.1)* | *1.11 (1.05 to 1.18)* |
| *Asia* | *18598841.4 (13403782.7-25748422.2)* | *566.7 (409.5-783.9)* | *40591623.1 (28924411.8-56833433.1)* | | *843.1 (599.1-1178)* | *1.45 (1.39 to 1.5)* |
| *Central Asia* | *118817.9 (79820.2-174950)* | *175.7 (118.4-257.8)* | *237958.9 (163368.4-333464.7)* | | *240.5 (164.6-337.3)* | *1.12 (1.06 to 1.18)* |
| *Southeast Asia* | *3682948.8 (2589198.8-5201509.9)* | *773.1 (546.2-1094.3)* | *10520027.7 (7378813.9-14809823.5)* | | *1404.1 (984.5-1974.7)* | *2.22 (2.11 to 2.32)* |
| *East Asia* | *5607180.9 (3957250.2-7863221.6)* | *408.1 (290-572.2)* | *10490358.5 (7423407.5-14808757.1)* | | *740.8 (519.6-1039.3)* | *2.05 (1.88 to 2.22)* |
| *Africa* | *2299016.3 (1591793.5-3250396.7)* | *398 (275.2-561.4)* | *6872240 (4758221.4-9660622.2)* | | *508.6 (354-715.6)* | *0.74 (0.67 to 0.81)* |
| *Central Africa* | *145214.1 (99833.7-212042.4)* | *248 (172-357.8)* | *546587 (373630.6-777685.4)* | | *339.5 (233.4-482.5)* | *0.9 (0.77 to 1.02)* |
| *Eastern Africa* | *462221.8 (321386.7-665530.8)* | *299 (210.3-428)* | *1580095.5 (1088569-2252159.8)* | | *410.8 (287.2-584.8)* | *1.08 (1.01 to 1.14)* |
| *Northern Africa* | *963191.4 (662484.9-1371449.6)* | *817.1 (562.9-1162.1)* | *2345085.7 (1633867.5-3341591.9)* | | *1076.4 (750.2-1533.8)* | *0.88 (0.85 to 0.91)* |
| *Western Africa* | *390480.1 (272779.4-565587.5)* | *247.6 (174.5-354.7)* | *1535927.4 (1069592.2-2198735.1)* | | *374.4 (264.1-532.4)* | *1.09 (0.92 to 1.27)* |
| *North America* | *4469727 (3157989.7-6274823.9)* | *1483.7 (1046.5-2088.3)* | *6362255.1 (4743009.3-8324162.7)* | | *1855.8 (1382.4-2426.5)* | *-0.52 (-1.01 to -0.04)* |
| *North Africa and Middle East* | *2463301.1 (1707181.3-3501813.4)* | *754.3 (523-1071.5)* | *6673431.5 (4672056.3-9434543.5)* | | *990.2 (693.3-1399.1)* | *1 (0.96 to 1.05)* |
| *Southern Sub-Saharan Africa* | *232808.6 (160575-334631.4)* | *449.2 (310.3-643.1)* | *480389.6 (328945.2-679589.3)* | | *551.2 (377.3-779.2)* | *0.68 (0.58 to 0.78)* |
| *Eastern Sub-Saharan Africa* | *448071 (310256.9-646236.2)* | *266.9 (188.2-383.1)* | *1438942.4 (1001296.8-2063339.5)* | | *342.7 (242-486.2)* | *0.83 (0.8 to 0.87)* |
| *Western Sub-Saharan Africa* | *438598.3 (307139.7-633877.8)* | *252.3 (177.7-360.9)* | *1741945.4 (1209889.2-2493010.7)* | | *378 (265.9-537)* | *1.06 (0.89 to 1.23)* |
| *Central Sub-Saharan Africa* | *110521.9 (76168.2-160819.7)* | *226.9 (157.8-329.5)* | *444120.8 (305191.6-640542)* | | *337.7 (233.3-485.4)* | *1.23 (1.1 to 1.37)* |
| *America* | *8155097.4 (5773666.2-11350176.3)* | *1100.4 (779.7-1529.1)* | *13334863.9 (9771560.1-18081294.3)* | | *1286.3 (942.5-1744.3)* | *-0.2 (-0.46 to 0.06)* |
| *Tropical Latin America* | *448844.2 (304409-647659.2)* | *283.9 (192.7-407.2)* | *746471.7 (514677-1057462.8)* | | *308.2 (211.9-438.9)* | *-0.16 (-0.32 to 0)* |
| *Central Latin America* | *2295522 (1578370.6-3199568.8)* | *1382.5 (952.5-1915.8)* | *4072957.2 (2852240.6-5662452.4)* | | *1518.7 (1063-2110.8)* | *-0.1 (-0.26 to 0.05)* |
| *Latin America and Caribbean* | *3433164.3 (2347793.1-4820492.3)* | *855.4 (587.2-1200.7)* | *6353275.2 (4457452.4-8841849.3)* | | *1012.5 (710.1-1408.9)* | *0.22 (0.1 to 0.35)* |
| *Andean Latin America* | *466365.8 (322494-653112.7)* | *1230.4 (850.9-1717.8)* | *1172864.5 (808337.4-1649165.9)* | | *1662.5 (1148.2-2338.2)* | *1.02 (0.94 to 1.1)* |
| *Southern-Latin America* | *296321.5 (204840.7-431020.1)* | *600.5 (416.5-874.7)* | *667604.8 (469021.5-956761.4)* | | *950.5 (666.4-1360.4)* | *1.45 (1.23 to 1.66)* |
| *United-States-of America* | *4254919.1 (3008393.8-5969707)* | *1568 (1107.3-2207)* | *6051074.9 (4526937.1-7901919.8)* | | *1958.7 (1463.5-2556.9)* | *-0.6 (-1.12 to -0.09)* |
| *Region-of-the-Americas* | *8155097.4 (5773666.2-11350176.3)* | *1100.4 (779.7-1529.1)* | *13334863.9 (9771560.1-18081294.3)* | | *1286.3 (942.5-1744.3)* | *-0.2 (-0.46 to 0.06)* |
| *American Samoa* | *490.6 (341.6-691.2)* | *989 (687-1397)* | *662.3 (463.1-968.6)* | | *1368.2 (952.6-1998.6)* | *0.93 (0.74 to 1.12)* |
| *Oceania* | *40226.7 (27505-56891.6)* | *621.1 (428.1-873.4)* | *124484.3 (86570.2-177516.4)* | | *870.3 (605-1243.3)* | *0.84 (0.67 to 1.02)* |
| *Europe* | *7534911.5 (5318909.7-10451156.5)* | *932.8 (657.5-1292.7)* | *8587063.7 (6032644.8-12086938.9)* | | *1104.7 (774.4-1555.8)* | *0.48 (0.46 to 0.51)* |
| *Eastern Europe* | *237422.1 (160523.3-339244.5)* | *103.8 (70-149.6)* | *265678.7 (185374.4-381763)* | | *132.4 (91.2-190.5)* | *0.96 (0.92 to 1.01)* |
| *Western Europe* | *6815142.5 (4796290.8-9455180.2)* | *1751.6 (1233.8-2431.9)* | *7455929.3 (5232259.4-10460556.8)* | | *1944.3 (1361.7-2726.7)* | *0.21 (0.14 to 0.27)* |
| *Central Europe* | *116152 (76721-173921.5)* | *91.9 (60.8-137.8)* | *119149.2 (82050.4-168514)* | | *111.7 (77-159.2)* | *0.58 (0.52 to 0.64)* |

**Table 1 Changes in the number of incident cases, prevalent cases, age-standardized incidence rate (ASIR), age-standardized prevalence rate (ASPR), and estimated annual percent change (EAPC) of PCOS globally and in GBD regions in 1990 and 2021**

| *Location* | DALYs | | | | | |
| --- | --- | --- | --- | --- | --- | --- |
|  | 1990 | | 2021 | | | EAPC_CI |
|  | Numbers(95%UI) | Age-standardized rate  (per100000)(95%UI) | Numbers(95%UI) | Age-standardized rate  (per100000)(95%UI) | |  |
| *Global* | *323798.6 (144342.1-675926.8)* | *6 (2.7-12.4)* | *607756.9 (272745.2-1268607.2)* | *7.6 (3.4-15.9)* | | *0.73 (0.69 to 0.76)* |
| *High SDI* | *122087.1 (55322.3-254195)* | *13.1 (5.9-27.2)* | *154313.3 (70664.1-314967.1)* | *15.2 (7-31)* | | *0.01 (-0.16 to 0.18)* |
| *High-middle SDI* | *61793.6 (27718.6-128454.5)* | *5.4 (2.4-11.3)* | *97184.5 (43255.1-205403.8)* | *7.7 (3.4-16.1)* | | *1.16 (1.1 to 1.21)* |
| *Middle SDI* | *93350.2 (41377-195580.3)* | *5.1 (2.2-10.7)* | *214898.8 (95592.6-450661.8)* | *8.5 (3.8-17.8)* | | *1.75 (1.69 to 1.81)* |
| *Low-middle SDI* | *36511.8 (15811.4-76977.5)* | *3.3 (1.4-6.8)* | *106509.7 (46621.4-223924.9)* | *5.2 (2.3-10.9)* | | *1.62 (1.59 to 1.66)* |
| *Low SDI* | *9836.5 (4213.8-20814.4)* | *2.2 (0.9-4.7)* | *34425.2 (14799.7-72773.1)* | *3.1 (1.4-6.6)* | | *1.23 (1.2 to 1.25)* |
| *World-Bank-High Income* | *144805.2 (66290.7-301489.4)* | *13.8 (6.3-28.7)* | *173638.6 (79806.4-356835.8)* | *15.6 (7.1-31.9)* | | *-0.02 (-0.17 to 0.13)* |
| *World-Bank-Upper-Middle Income* | *95385.9 (41793.4-200416.2)* | *4.2 (1.9-8.9)* | *182847 (80899.9-382594)* | *7.1 (3.1-14.8)* | | *1.64 (1.54 to 1.74)* |
| *World-Bank-Lower-Middle Income* | *72754.3 (31705.3-151658.8)* | *3.9 (1.7-8)* | *222619.6 (97700.5-466783.1)* | *6.3 (2.8-13.2)* | | *1.8 (1.72 to 1.88)* |
| *World-Bank Low Income* | *8090.6 (3447.9-16957)* | *2.7 (1.2-5.7)* | *25782.9 (11268.1-54535.9)* | *3.6 (1.6-7.6)* | | *0.97 (0.94 to 0.99)* |
| *Commonwealth High Income* | *14290.3 (6398.2-29720.3)* | *12.3 (5.5-25.6)* | *20984.7 (9551.9-43821.4)* | *15.5 (7-32.3)* | | *0.55 (0.45 to 0.66)* |
| *Commonwealth-Middle-Income* | *35888.3 (15693.7-75336.6)* | *3.1 (1.4-6.5)* | *117820.5 (51622.1-246275.3)* | *5.2 (2.3-10.9)* | | *1.87 (1.8 to 1.95)* |
| *Commonwealth Low Income* | *3955.9 (1685.1-8333.6)* | *2.1 (0.9-4.3)* | *12239.4 (5284.6-25711.2)* | *3.1 (1.3-6.5)* | | *1.39 (1.34 to 1.44)* |
| *South Asia- WB* | *31269.1 (13687.6-66187.9)* | *2.8 (1.2-6)* | *103813.6 (45375.4-217015.1)* | *5 (2.2-10.4)* | | *2.1 (1.99 to 2.21)* |
| *Europe&Central Asia - WB* | *67970.2 (30712.8-141096.5)* | *8 (3.6-16.5)* | *77466.9 (35037.9-161551.3)* | *9.1 (4.1-19)* | | *0.37 (0.34 to 0.4)* |
| *East-Asia& Pacific - WB* | *121819.4 (53563.3-248621.9)* | *6 (2.6-12.2)* | *221741.8 (99008.6-453970.7)* | *9.5 (4.3-19.6)* | | *1.61 (1.56 to 1.67)* |
| *Middle East & North Africa - WB* | *18065.2 (7904.8-38138.7)* | *7.3 (3.2-15.3)* | *47725.9 (21256.2-101198)* | *9.2 (4.1-19.5)* | | *0.87 (0.79 to 0.96)* |
| *Sub-Saharan Africa - WB* | *11697.6 (4975.5-24792.7)* | *2.5 (1.1-5.3)* | *39456.1 (17164.4-83265.7)* | *3.4 (1.5-7.2)* | | *0.92 (0.84 to 1)* |
| *Latin America & Caribbean - WB* | *32765.7 (14429.1-67730.8)* | *7.3 (3.2-15)* | *60944 (26973-126410)* | *8.8 (3.9-18.2)* | | *0.32 (0.22 to 0.43)* |
| *Advanced Health System* | *153790.1 (70341.5-319860.6)* | *11.3 (5.2-23.4)* | *185952.4 (85392.4-382110.3)* | *13.1 (6-26.9)* | | *0.12 (-0.02 to 0.26)* |
| *Basic Health System* | *123699.3 (54098.2-260032.5)* | *5 (2.2-10.5)* | *266136.4 (118158.6-556135.2)* | *8.4 (3.7-17.6)* | | *1.75 (1.67 to 1.83)* |
| *Limited Health System* | *43614.4 (18973.8-91859)* | *2.9 (1.3-6.1)* | *145769.4 (63565.2-306376)* | *4.8 (2.1-10)* | | *1.83 (1.76 to 1.9)* |
| *Minimal Health System* | *2475.3 (1058.1-5180.5)* | *2.1 (0.9-4.4)* | *9473.2 (4080.6-20118.6)* | *3 (1.3-6.3)* | | *1.11 (1.03 to 1.18)* |
| *Asia* | *163096.6 (71792-338188.2)* | *4.9 (2.2-10.3)* | *354057 (156107-731797.9)* | *7.4 (3.2-15.2)* | | *1.44 (1.39 to 1.5)* |
| *Central Asia* | *1049.1 (438.1-2236.9)* | *1.5 (0.6-3.3)* | *2079.4 (886.9-4469.5)* | *2.1 (0.9-4.5)* | | *1.11 (1.05 to 1.17)* |
| *Southeast Asia* | *32853.8 (14429.1-66791.8)* | *6.9 (3-14)* | *92605.7 (41149-191024.1)* | *12.4 (5.5-25.5)* | | *2.18 (2.09 to 2.28)* |
| *East Asia* | *48225.9 (20818.6-100143)* | *3.5 (1.5-7.3)* | *89991.4 (39441.8-185701.9)* | *6.4 (2.8-13.2)* | | *2.06 (1.89 to 2.24)* |
| *Africa* | *20445.4 (8874.8-43350.5)* | *3.5 (1.5-7.4)* | *60192.9 (26651.9-127793.2)* | *4.4 (2-9.4)* | | *0.69 (0.62 to 0.76)* |
| *Central Africa* | *1262.2 (537.3-2655.3)* | *2.1 (0.9-4.5)* | *4768.9 (2060.6-9892.1)* | *2.9 (1.3-6.1)* | | *0.89 (0.75 to 1.03)* |
| *Eastern Africa* | *4043.1 (1726.4-8543.1)* | *2.6 (1.1-5.5)* | *13730.2 (6046.1-28860.7)* | *3.5 (1.6-7.5)* | | *1.07 (1.01 to 1.12)* |
| *Northern Africa* | *8789 (3855.5-18483.1)* | *7.4 (3.2-15.6)* | *20848.8 (9244.5-43732)* | *9.6 (4.2-20.1)* | | *0.81 (0.79 to 0.84)* |
| *Western Africa* | *3395.8 (1453-7141.5)* | *2.1 (0.9-4.5)* | *13366.3 (5760.7-28368.4)* | *3.2 (1.4-6.9)* | | *1.09 (0.91 to 1.26)* |
| *North America* | *39977.3 (17689.4-82640.8)* | *13.3 (5.9-27.3)* | *56162.3 (25718.6-113710.7)* | *16.4 (7.6-33.3)* | | *-0.54 (-1.01 to -0.05)* |
| *North Africa and Middle East* | *22386.5 (9812.9-47102.6)* | *6.8 (3-14.3)* | *59116.3 (26476.8-125706.8)* | *8.8 (3.9-18.7)* | | *0.96 (0.9 to 1.01)* |
| *Southern Sub-Saharan Africa* | *2047.4 (881.8-4435.2)* | *3.9 (1.7-8.5)* | *4157.7 (1795.1-8746)* | *4.8 (2.1-10)* | | *0.64 (0.54 to 0.74)* |
| *Eastern Sub-Saharan Africa* | *3903.3 (1645.1-8198.6)* | *2.3 (1-4.9)* | *12497.1 (5380.7-26310.2)* | *3 (1.3-6.2)* | | *0.82 (0.79 to 0.86)* |
| *Western Sub-Saharan Africa* | *3816.2 (1629-8015.4)* | *2.2 (0.9-4.6)* | *15176.8 (6521.5-32235.9)* | *3.3 (1.4-6.9)* | | *1.05 (0.88 to 1.23)* |
| *Central Sub-Saharan Africa* | *958.3 (406.3-1975.2)* | *1.9 (0.8-4.1)* | *3861.9 (1673.7-7930.5)* | *2.9 (1.3-6)* | | *1.23 (1.08 to 1.38)* |
| *America* | *72424.3 (32262-149675.4)* | *9.8 (4.3-20.2)* | *116794.5 (53755.4-243772.3)* | *11.3 (5.2-23.5)* | | *-0.22 (-0.49 to 0.04)* |
| *Tropical Latin America* | *4011.1 (1701.1-8427.3)* | *2.5 (1.1-5.3)* | *6557.7 (2825.1-13831)* | *2.7 (1.2-5.7)* | | *-0.18 (-0.34 to -0.01)* |
| *Central Latin America* | *20163.7 (9003.8-42043)* | *12.1 (5.4-25.3)* | *35314.1 (15577.8-73735.7)* | *13.2 (5.8-27.5)* | | *-0.12 (-0.27 to 0.04)* |
| *Latin America and Caribbean* | *30210.5 (13328.4-62616.6)* | *7.5 (3.3-15.6)* | *55162.5 (24274.2-114672.1)* | *8.8 (3.9-18.3)* | | *0.21 (0.09 to 0.33)* |
| *Andean Latin America* | *4055.9 (1797.3-8748.4)* | *10.7 (4.8-23)* | *10129.2 (4423.9-21176.6)* | *14.4 (6.3-30)* | | *1 (0.92 to 1.08)* |
| *Southern-Latin America* | *2627.8 (1179.4-5421.3)* | *5.3 (2.4-11)* | *5893.5 (2574.8-12247.5)* | *8.4 (3.7-17.4)* | | *1.44 (1.23 to 1.65)* |
| *United-States-of America* | *38051.1 (16838.4-78573.6)* | *14 (6.2-28.8)* | *53388.1 (24521.8-107618.8)* | *17.3 (8-34.9)* | | *-0.62 (-1.13 to -0.1)* |
| *Region-of-the-Americas* | *72424.3 (32262-149675.4)* | *9.8 (4.3-20.2)* | *116794.5 (53755.4-243772.3)* | *11.3 (5.2-23.5)* | | *-0.22 (-0.49 to 0.04)* |
| *American Samoa* | *4.3 (1.9-9)* | *8.7 (3.8-18.2)* | *5.8 (2.5-12.2)* | *12 (5.2-25.3)* | | *0.92 (0.73 to 1.1)* |
| *Oceania* | *352.2 (160.6-738.9)* | *5.4 (2.5-11.3)* | *1083.5 (471.3-2285.6)* | *7.6 (3.3-15.9)* | | *0.84 (0.67 to 1.01)* |
| *Europe* | *67270.7 (30434-139695.2)* | *8.3 (3.8-17.3)* | *75944 (34374.6-158081.6)* | *9.8 (4.4-20.5)* | | *0.47 (0.45 to 0.5)* |
| *Eastern Europe* | *2097.7 (868.6-4466.8)* | *0.9 (0.4-2)* | *2323.7 (970-4890.4)* | *1.2 (0.5-2.5)* | | *0.95 (0.91 to 1)* |
| *Western Europe* | *60788.9 (27584.9-126652.6)* | *15.6 (7.1-32.5)* | *66041.5 (29857.2-136985.1)* | *17.3 (7.8-35.9)* | | *0.21 (0.15 to 0.27)* |
| *Central Europe* | *1014.7 (425.9-2088.9)* | *0.8 (0.3-1.7)* | *1032.7 (442.9-2156.6)* | *1 (0.4-2)* | | *0.58 (0.53 to 0.63)* |
| *location* | YLDs | | | | | |
|  | 1990 | | 2021 | | | EAPC_CI |
|  | Numbers(95%UI) | Age-standardized rate  (per100000)(95%UI) | Numbers(95%UI) | | Age-standardized rate  (per100000)(95%UI) |  |
| *Global* | *323798.589482073 (144342.1-675926.8)* | *6 (2.7-12.4)* | *607756.9 (272745.2-1268607.2)* | | *7.6 (3.4-15.9)* | *0.73 (0.69 to 0.76)* |
| *High SDI* | *122087.069310582 (55322.3-254195)* | *13.1 (5.9-27.2)* | *154313.3 (70664.1-314967.1)* | | *15.2 (7-31)* | *0.01 (-0.16 to 0.18)* |
| *High-middle SDI* | *61793.5829121228 (27718.6-128454.5)* | *5.4 (2.4-11.3)* | *97184.5 (43255.1-205403.8)* | | *7.7 (3.4-16.1)* | *1.16 (1.1 to 1.21)* |
| *Middle SDI* | *93350.1643457733 (41377-195580.3)* | *5.1 (2.2-10.7)* | *214898.8 (95592.6-450661.8)* | | *8.5 (3.8-17.8)* | *1.75 (1.69 to 1.81)* |
| *Low-middle SDI* | *36511.8299999773 (15811.4-76977.5)* | *3.3 (1.4-6.8)* | *106509.7 (46621.4-223924.9)* | | *5.2 (2.3-10.9)* | *1.62 (1.59 to 1.66)* |
| *Low SDI* | *9836.51670213337 (4213.8-20814.4)* | *2.2 (0.9-4.7)* | *34425.2 (14799.7-72773.1)* | | *3.1 (1.4-6.6)* | *1.23 (1.2 to 1.25)* |
| *World-Bank-High Income* | *148344.057971075 (67911.7-308843.6)* | *13.8 (6.3-28.7)* | *176971.3 (81338.8-363690.1)* | | *15.6 (7.1-31.9)* | *-0.02 (-0.17 to 0.13)* |
| *World-Bank-Upper-Middle Income* | *92337.6034404174 (40457.1-194018.7)* | *4.2 (1.9-8.9)* | *176013.8 (77879.2-368272.8)* | | *7.1 (3.1-14.8)* | *1.64 (1.54 to 1.74)* |
| *World-Bank-Lower-Middle Income* | *75357.6971773956 (32839.8-157087.2)* | *3.9 (1.7-8)* | *230128.8 (100994.3-482517.2)* | | *6.3 (2.8-13.2)* | *1.8 (1.72 to 1.88)* |
| *World-Bank Low Income* | *7537.83570331432 (3212.6-15799)* | *2.7 (1.2-5.7)* | *24214.9 (10583.8-51228.4)* | | *3.6 (1.6-7.6)* | *0.95 (0.92 to 0.98)* |
| *Commonwealth High Income* | *14290.2893678241 (6398.2-29720.3)* | *12.3 (5.5-25.6)* | *20984.7 (9551.9-43821.4)* | | *15.5 (7-32.3)* | *0.55 (0.45 to 0.66)* |
| *Commonwealth-Middle-Income* | *35888.3394775411 (15693.7-75336.6)* | *3.1 (1.4-6.5)* | *117820.5 (51622.1-246275.3)* | | *5.2 (2.3-10.9)* | *1.87 (1.8 to 1.95)* |
| *Commonwealth Low Income* | *3955.91199916162 (1685.1-8333.6)* | *2.1 (0.9-4.3)* | *12239.4 (5284.6-25711.2)* | | *3.1 (1.3-6.5)* | *1.39 (1.34 to 1.44)* |
| *South Asia- WB* | *31269.0991459791 (13687.6-66187.9)* | *2.8 (1.2-6)* | *103813.6 (45375.4-217015.1)* | | *5 (2.2-10.4)* | *2.1 (1.99 to 2.21)* |
| *Europe&Central Asia - WB* | *67970.2186712524 (30712.8-141096.5)* | *8 (3.6-16.5)* | *77466.9 (35037.9-161551.3)* | | *9.1 (4.1-19)* | *0.37 (0.34 to 0.4)* |
| *East-Asia& Pacific - WB* | *121819.36675242 (53563.3-248621.9)* | *6 (2.6-12.2)* | *221741.8 (99008.6-453970.7)* | | *9.5 (4.3-19.6)* | *1.61 (1.56 to 1.67)* |
| *Middle East & North Africa - WB* | *18065.1887639013 (7904.8-38138.7)* | *7.3 (3.2-15.3)* | *47725.9 (21256.2-101198)* | | *9.2 (4.1-19.5)* | *0.87 (0.79 to 0.96)* |
| *Sub-Saharan Africa - WB* | *11697.5579656459 (4975.5-24792.7)* | *2.5 (1.1-5.3)* | *39456.1 (17164.4-83265.7)* | | *3.4 (1.5-7.2)* | *0.92 (0.84 to 1)* |
| *Latin America & Caribbean - WB* | *32765.70733679 (14429.1-67730.8)* | *7.3 (3.2-15)* | *60944 (26973-126410)* | | *8.8 (3.9-18.2)* | *0.32 (0.22 to 0.43)* |
| *Advanced Health System* | *153790.104897037 (70341.5-319860.6)* | *11.3 (5.2-23.4)* | *185952.4 (85392.4-382110.3)* | | *13.1 (6-26.9)* | *0.12 (-0.02 to 0.26)* |
| *Basic Health System* | *123699.300319931 (54098.2-260032.5)* | *5 (2.2-10.5)* | *266136.4 (118158.6-556135.2)* | | *8.4 (3.7-17.6)* | *1.75 (1.67 to 1.83)* |
| *Limited Health System* | *43614.4187239326 (18973.8-91859)* | *2.9 (1.3-6.1)* | *145769.4 (63565.2-306376)* | | *4.8 (2.1-10)* | *1.83 (1.76 to 1.9)* |
| *Minimal Health System* | *2475.33932968792 (1058.1-5180.5)* | *2.1 (0.9-4.4)* | *9473.2 (4080.6-20118.6)* | | *3 (1.3-6.3)* | *1.11 (1.03 to 1.18)* |
| *Asia* | *163096.641322195 (71792-338188.2)* | *4.9 (2.2-10.3)* | *354057 (156107-731797.9)* | | *7.4 (3.2-15.2)* | *1.44 (1.39 to 1.5)* |
| *Central Asia* | *1049.10104293378 (438.1-2236.9)* | *1.5 (0.6-3.3)* | *2079.4 (886.9-4469.5)* | | *2.1 (0.9-4.5)* | *1.11 (1.05 to 1.17)* |
| *Southeast Asia* | *32853.7695118544 (14429.1-66791.8)* | *6.9 (3-14)* | *92605.7 (41149-191024.1)* | | *12.4 (5.5-25.5)* | *2.18 (2.09 to 2.28)* |
| *East Asia* | *48225.9138354218 (20818.6-100143)* | *3.5 (1.5-7.3)* | *89991.4 (39441.8-185701.9)* | | *6.4 (2.8-13.2)* | *2.06 (1.89 to 2.24)* |
| *Africa* | *20445.4270504934 (8874.8-43350.5)* | *3.5 (1.5-7.4)* | *60192.9 (26651.9-127793.2)* | | *4.4 (2-9.4)* | *0.69 (0.62 to 0.76)* |
| *Central Africa* | *1262.21358463437 (537.3-2655.3)* | *2.1 (0.9-4.5)* | *4768.9 (2060.6-9892.1)* | | *2.9 (1.3-6.1)* | *0.89 (0.75 to 1.03)* |
| *Eastern Africa* | *4043.08130172246 (1726.4-8543.1)* | *2.6 (1.1-5.5)* | *13730.2 (6046.1-28860.7)* | | *3.5 (1.6-7.5)* | *1.07 (1.01 to 1.12)* |
| *Northern Africa* | *8789.0076299909 (3855.5-18483.1)* | *7.4 (3.2-15.6)* | *20848.8 (9244.5-43732)* | | *9.6 (4.2-20.1)* | *0.81 (0.79 to 0.84)* |
| *Western Africa* | *3395.7592061856 (1453-7141.5)* | *2.1 (0.9-4.5)* | *13366.3 (5760.7-28368.4)* | | *3.2 (1.4-6.9)* | *1.09 (0.91 to 1.26)* |
| *North America* | *39977.2844158563 (17689.4-82640.8)* | *13.3 (5.9-27.3)* | *56162.3 (25718.6-113710.7)* | | *16.4 (7.6-33.3)* | *-0.54 (-1.01 to -0.05)* |
| *North Africa and Middle East* | *22386.4722474427 (9812.9-47102.6)* | *6.8 (3-14.3)* | *59116.3 (26476.8-125706.8)* | | *8.8 (3.9-18.7)* | *0.96 (0.9 to 1.01)* |
| *Southern Sub-Saharan Africa* | *2047.42256138018 (881.8-4435.2)* | *3.9 (1.7-8.5)* | *4157.7 (1795.1-8746)* | | *4.8 (2.1-10)* | *0.64 (0.54 to 0.74)* |
| *Eastern Sub-Saharan Africa* | *3903.29284241345 (1645.1-8198.6)* | *2.3 (1-4.9)* | *12497.1 (5380.7-26310.2)* | | *3 (1.3-6.2)* | *0.82 (0.79 to 0.86)* |
| *Western Sub-Saharan Africa* | *3816.2345113481 (1629-8015.4)* | *2.2 (0.9-4.6)* | *15176.8 (6521.5-32235.9)* | | *3.3 (1.4-6.9)* | *1.05 (0.88 to 1.23)* |
| *Central Sub-Saharan Africa* | *958.262724818009 (406.3-1975.2)* | *1.9 (0.8-4.1)* | *3861.9 (1673.7-7930.5)* | | *2.9 (1.3-6)* | *1.23 (1.08 to 1.38)* |
| *America* | *72424.3224473781 (32262-149675.4)* | *9.8 (4.3-20.2)* | *116794.5 (53755.4-243772.3)* | | *11.3 (5.2-23.5)* | *-0.22 (-0.49 to 0.04)* |
| *Tropical Latin America* | *4011.06973274821 (1701.1-8427.3)* | *2.5 (1.1-5.3)* | *6557.7 (2825.1-13831)* | | *2.7 (1.2-5.7)* | *-0.18 (-0.34 to -0.01)* |
| *Central Latin America* | *20163.6854279241 (9003.8-42043)* | *12.1 (5.4-25.3)* | *35314.1 (15577.8-73735.7)* | | *13.2 (5.8-27.5)* | *-0.12 (-0.27 to 0.04)* |
| *Latin America and Caribbean* | *30210.4768160263 (13328.4-62616.6)* | *7.5 (3.3-15.6)* | *55162.5 (24274.2-114672.1)* | | *8.8 (3.9-18.3)* | *0.21 (0.09 to 0.33)* |
| *Andean Latin America* | *4055.89352141989 (1797.3-8748.4)* | *10.7 (4.8-23)* | *10129.2 (4423.9-21176.6)* | | *14.4 (6.3-30)* | *1 (0.92 to 1.08)* |
| *Southern-Latin America* | *2627.82901292101 (1179.4-5421.3)* | *5.3 (2.4-11)* | *5893.5 (2574.8-12247.5)* | | *8.4 (3.7-17.4)* | *1.44 (1.23 to 1.65)* |
| *United-States-of America* | *38051.1197225066 (16838.4-78573.6)* | *14 (6.2-28.8)* | *53388.1 (24521.8-107618.8)* | | *17.3 (8-34.9)* | *-0.62 (-1.13 to -0.1)* |
| *Region-of-the-Americas* | *72424.3224473781 (32262-149675.4)* | *9.8 (4.3-20.2)* | *116794.5 (53755.4-243772.3)* | | *11.3 (5.2-23.5)* | *-0.22 (-0.49 to 0.04)* |
| *American Samoa* | *4.33704784748954 (1.9-9)* | *8.7 (3.8-18.2)* | *5.8 (2.5-12.2)* | | *12 (5.2-25.3)* | *0.92 (0.73 to 1.1)* |
| *Oceania* | *352.204781576058 (160.6-738.9)* | *5.4 (2.5-11.3)* | *1083.5 (471.3-2285.6)* | | *7.6 (3.3-15.9)* | *0.84 (0.67 to 1.01)* |
| *Europe* | *67270.7098701165 (30434-139695.2)* | *8.3 (3.8-17.3)* | *75944 (34374.6-158081.6)* | | *9.8 (4.4-20.5)* | *0.47 (0.45 to 0.5)* |
| *Eastern Europe* | *2097.6940278542 (868.6-4466.8)* | *0.9 (0.4-2)* | *2323.7 (970-4890.4)* | | *1.2 (0.5-2.5)* | *0.95 (0.91 to 1)* |
| *Western Europe* | *60788.9195069732 (27584.9-126652.6)* | *15.6 (7.1-32.5)* | *66041.5 (29857.2-136985.1)* | | *17.3 (7.8-35.9)* | *0.21 (0.15 to 0.27)* |
| *Central Europe* | *1014.73450311381 (425.9-2088.9)* | *0.8 (0.3-1.7)* | *1032.7 (442.9-2156.6)* | | *1 (0.4-2)* | *0.58 (0.53 to 0.63)* |

**Table 2 Changes in DALYs, YLDs, ASR_DALYs, ASR_DALYs, and EAPC of PCOS in the world and GBD regions in 1990 and 2021**
