# Supplementary figures and images for: The evolution of the global disease burden of polycystic ovary syndrome and the role of regional heterogeneity in high body mass index exposure: a spatiotemporal analysis based on the global burden of disease 2021
Source: Front Reprod Health. 2025 Nov 4;7:1600995. doi: 10.3389/frph.2025.1600995 (PMC12623367; doi:10.3389/frph.2025.1600995)

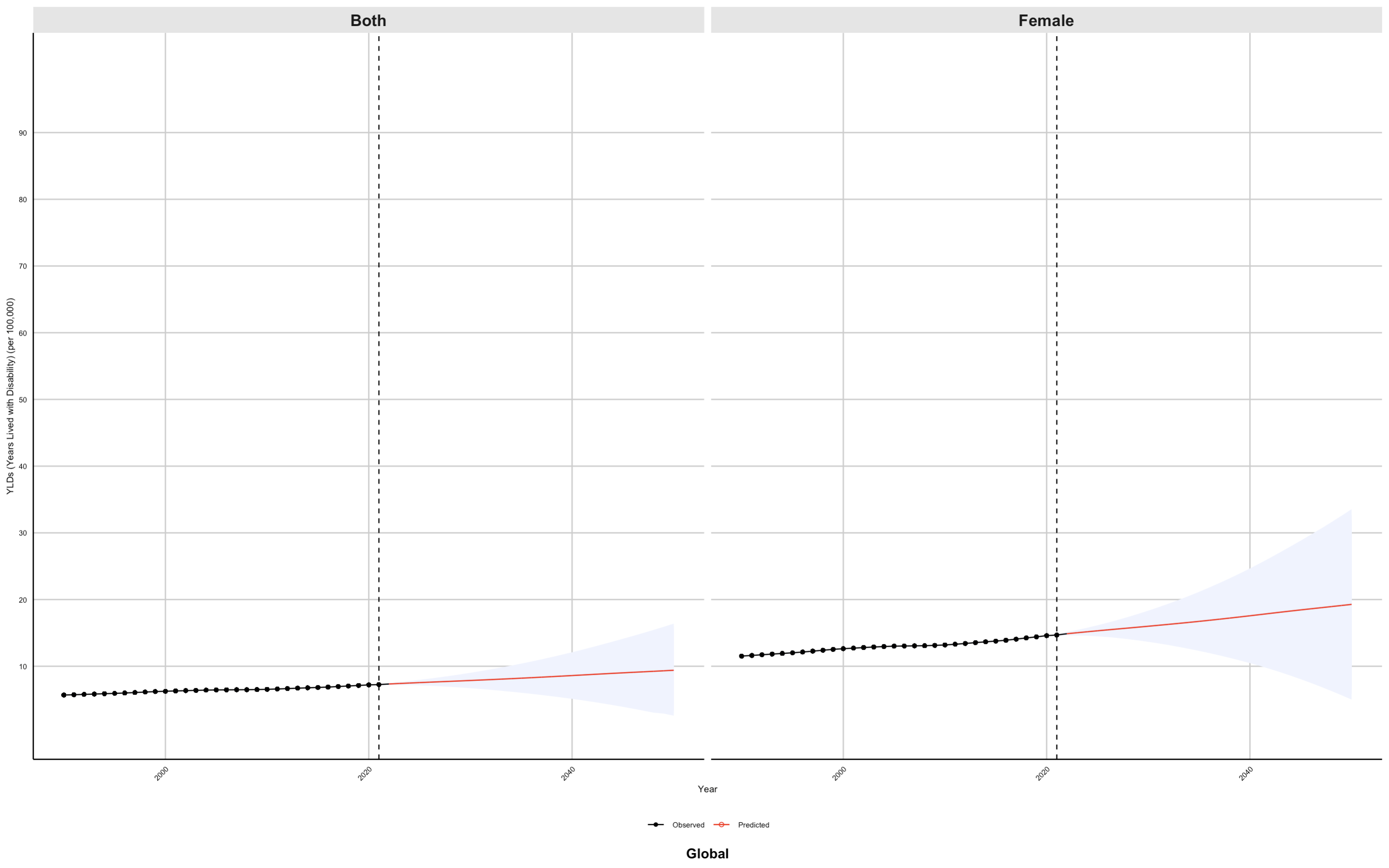

Supplement: Supplementary file 2 [file Supplementaryfile1.zip › Document/Document8-1/S25.png]

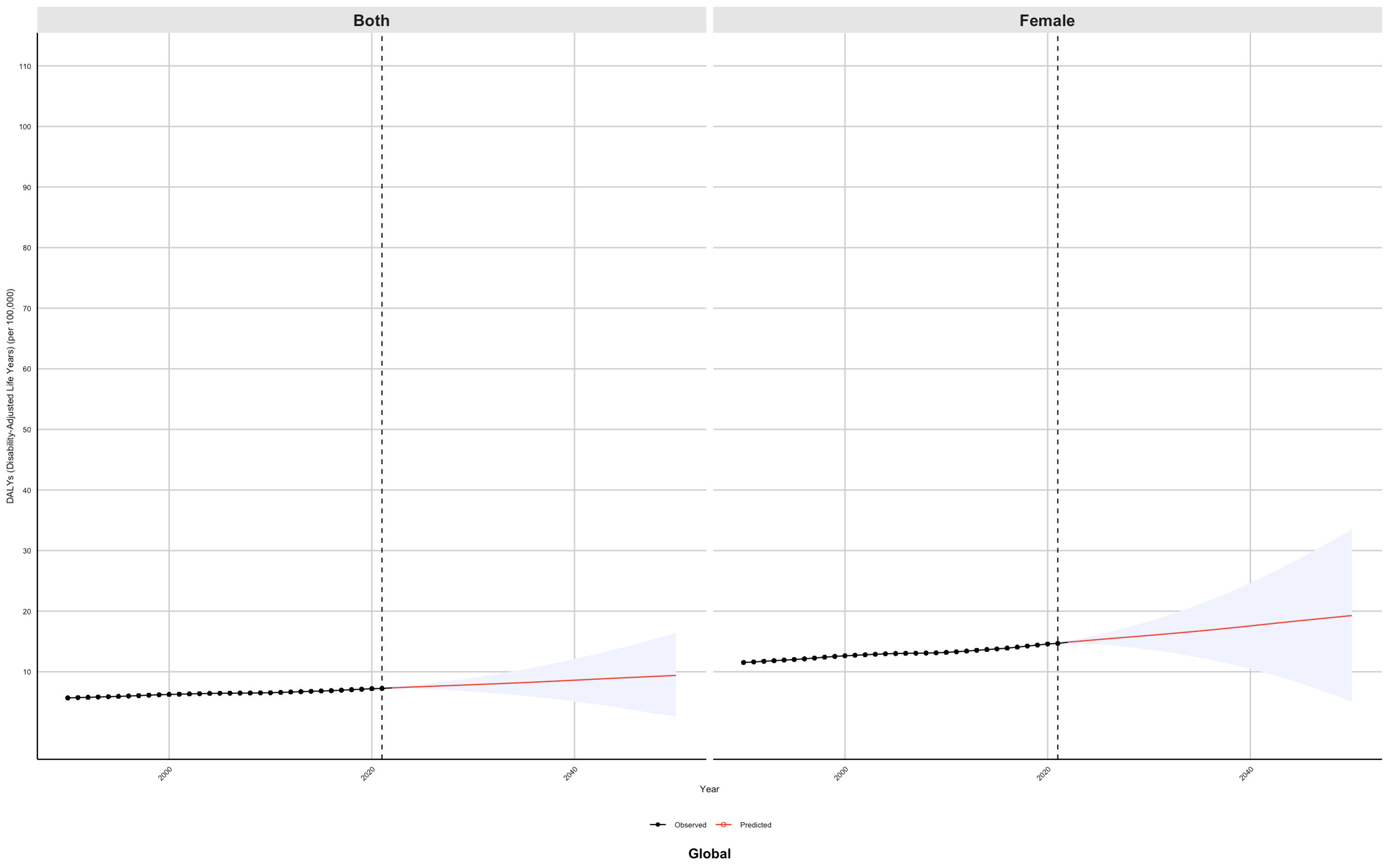

Supplement: Supplementary file 2 [file Supplementaryfile1.zip › Document/Document8-1/S24.png]

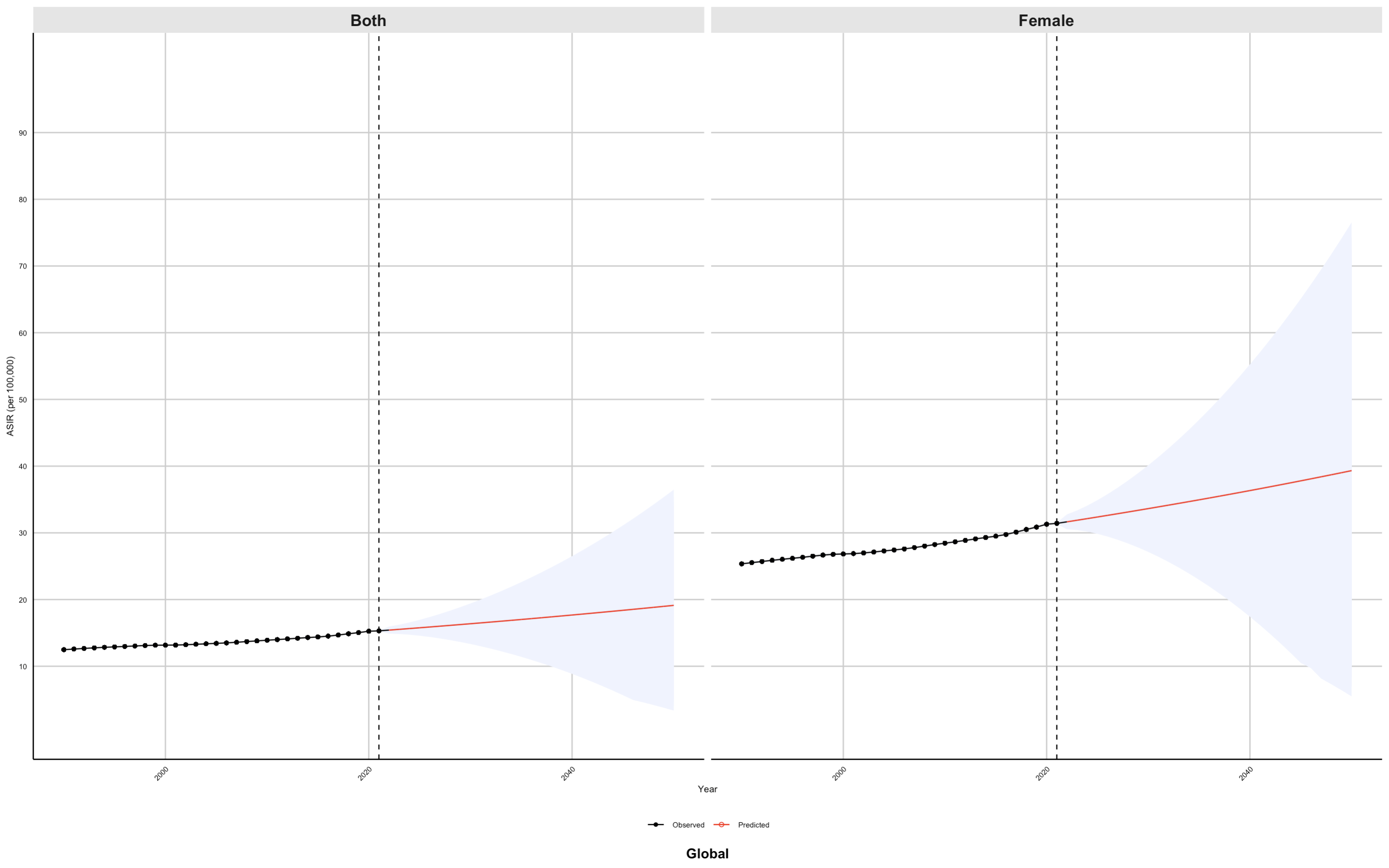

Supplement: Supplementary file 2 [file Supplementaryfile1.zip › Document/Document8-1/S23.png]

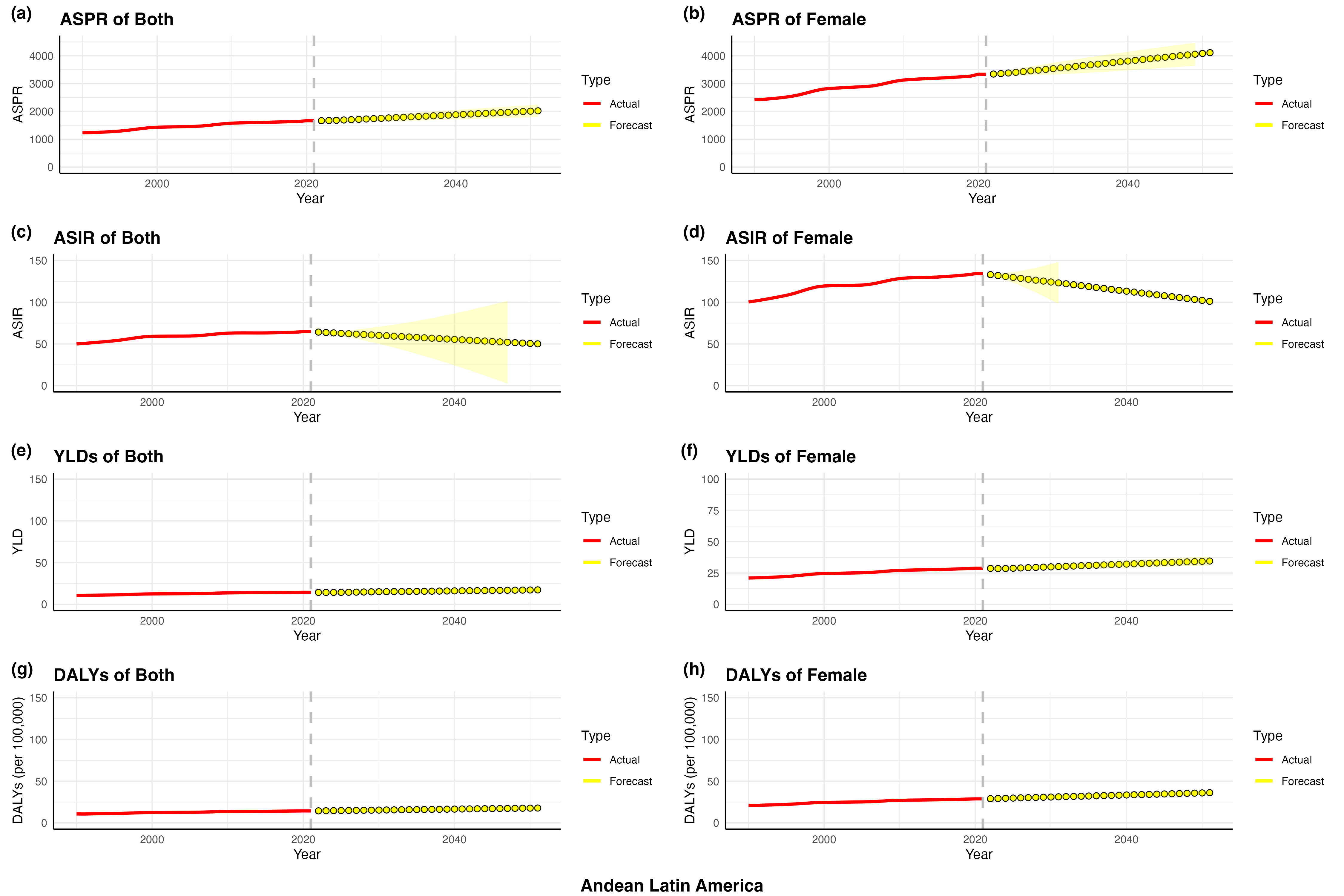

Supplement: Supplementary file 2 [file Supplementaryfile1.zip › Document/Document8-2/S 27/PCOS ARIMA Andean Latin America.png]

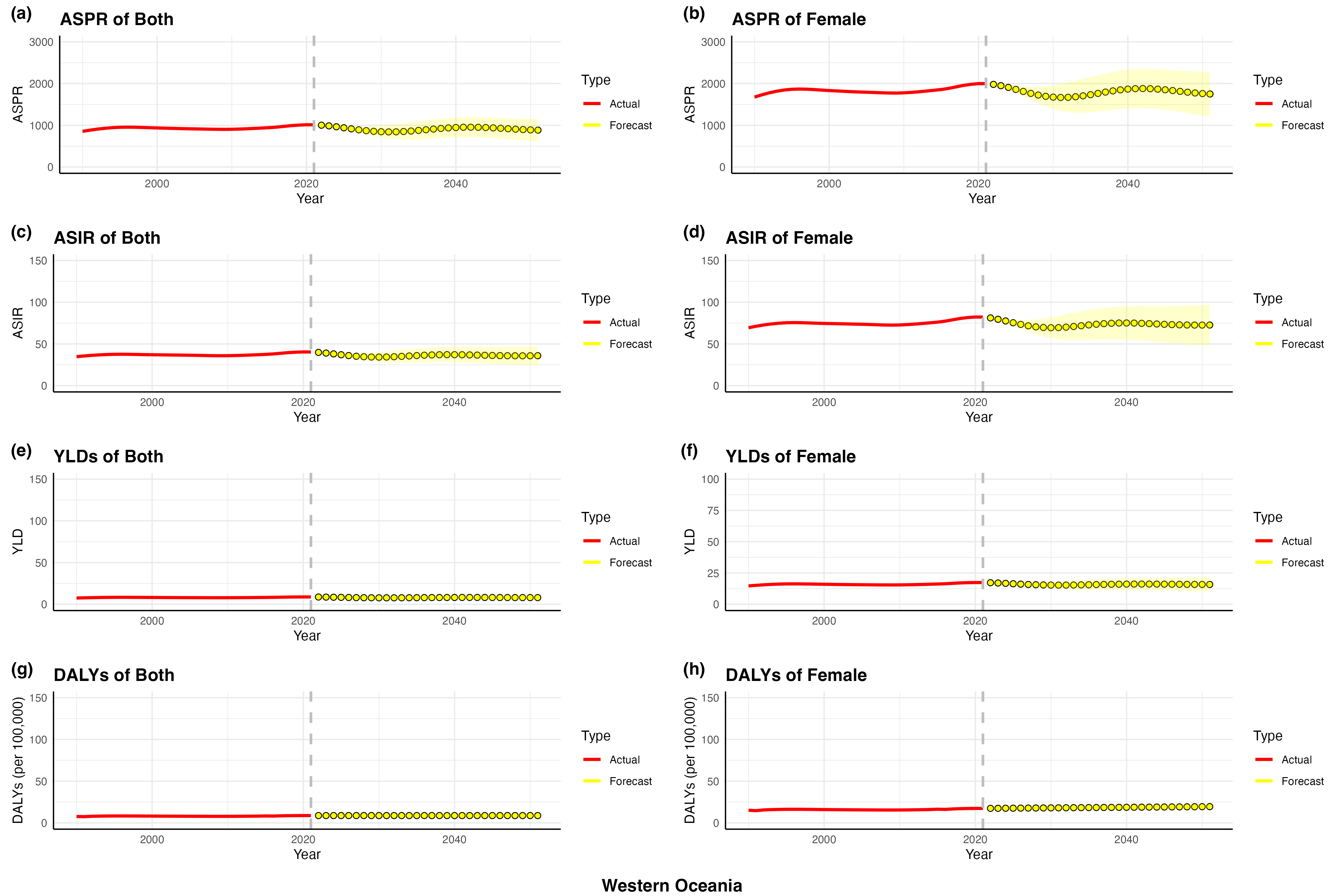

Supplement: Supplementary file 2 [file Supplementaryfile1.zip › Document/Document8-2/S 27/PCOS ARIMA Western Oceania.png]

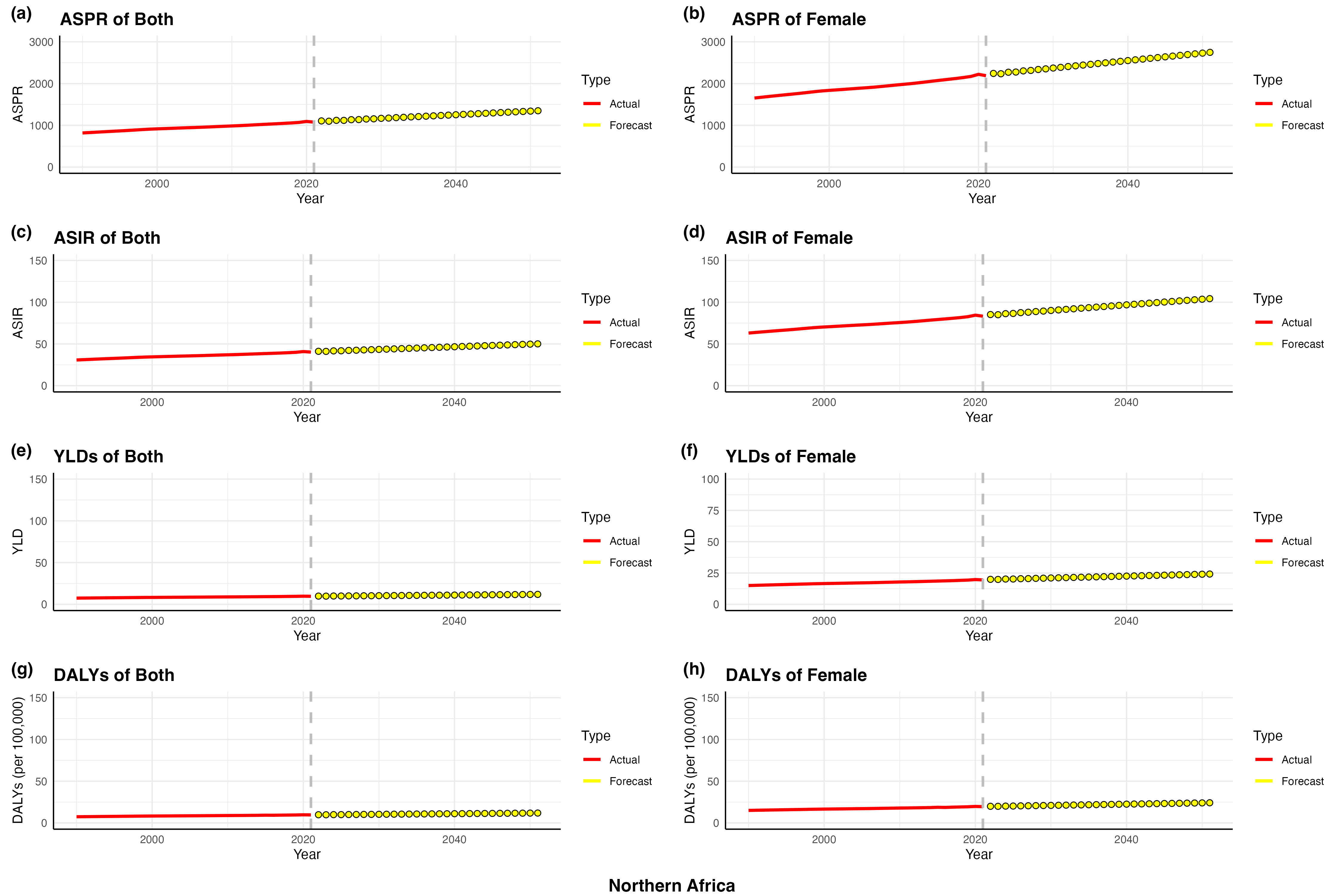

Supplement: Supplementary file 2 [file Supplementaryfile1.zip › Document/Document8-2/S 27/PCOS ARIMA Northern Africa.png]

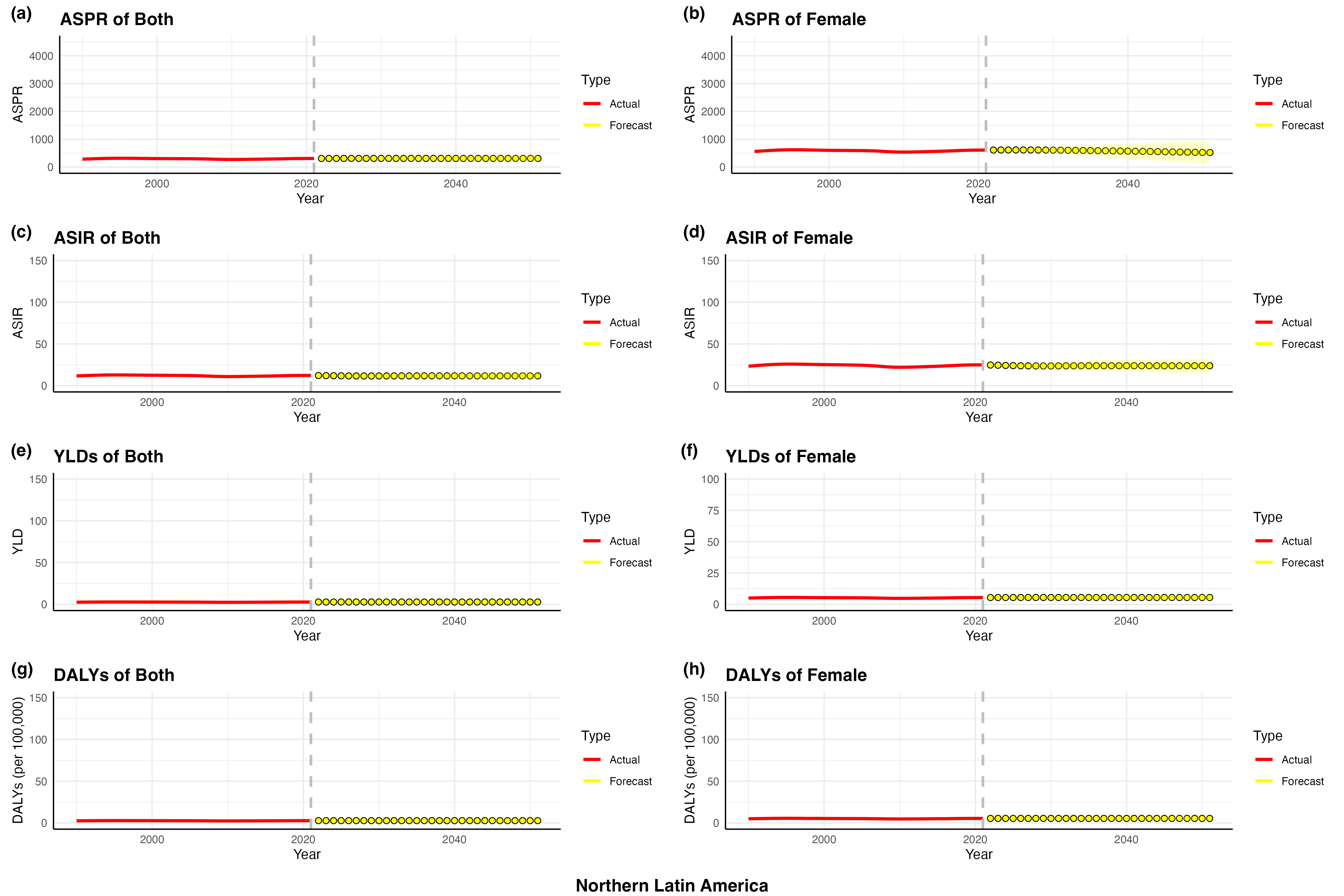

Supplement: Supplementary file 2 [file Supplementaryfile1.zip › Document/Document8-2/S 27/PCOS ARIMA Southeast Asia.png]

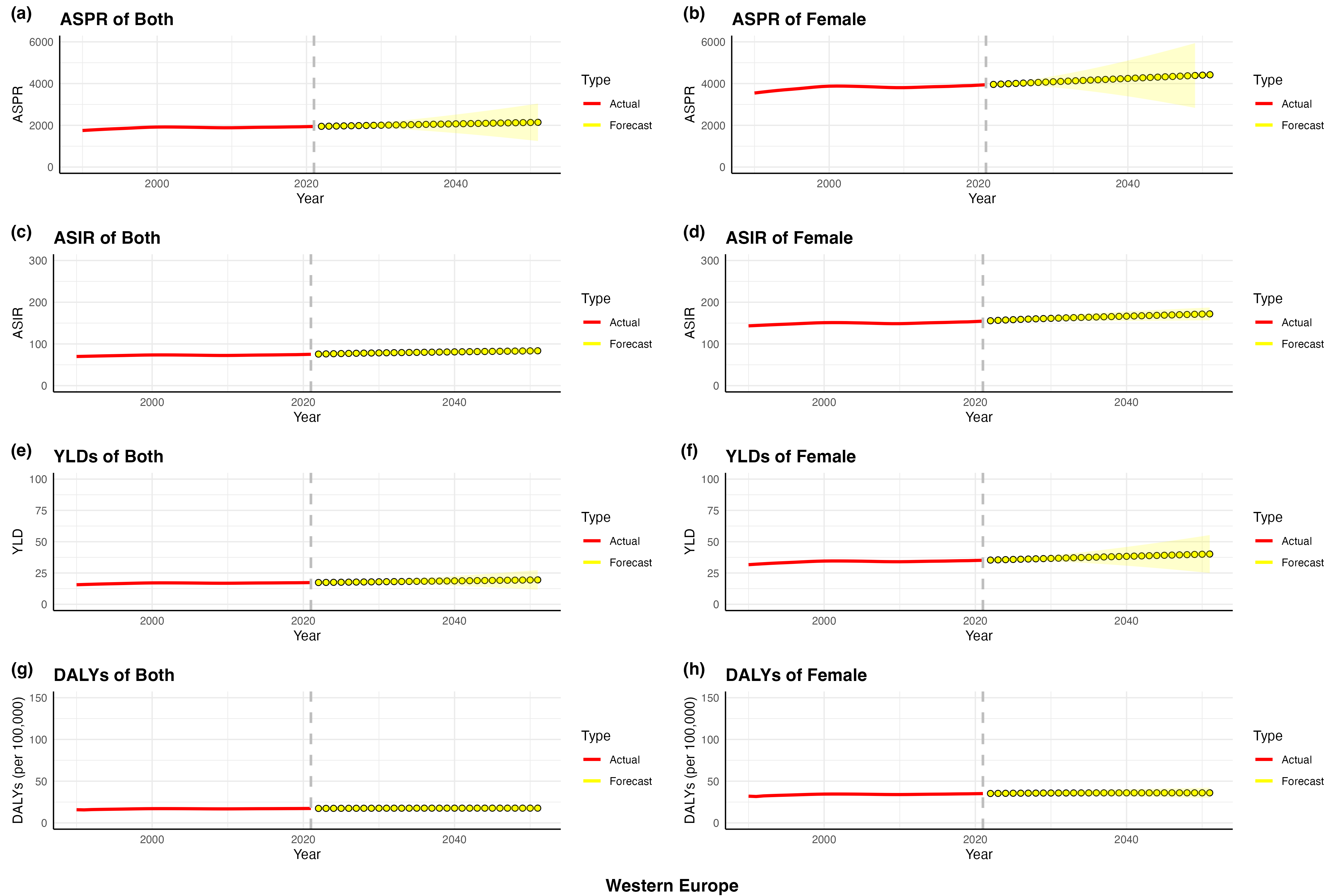

Supplement: Supplementary file 2 [file Supplementaryfile1.zip › Document/Document8-2/S 27/PCOS ARIMA Western Europe.png]

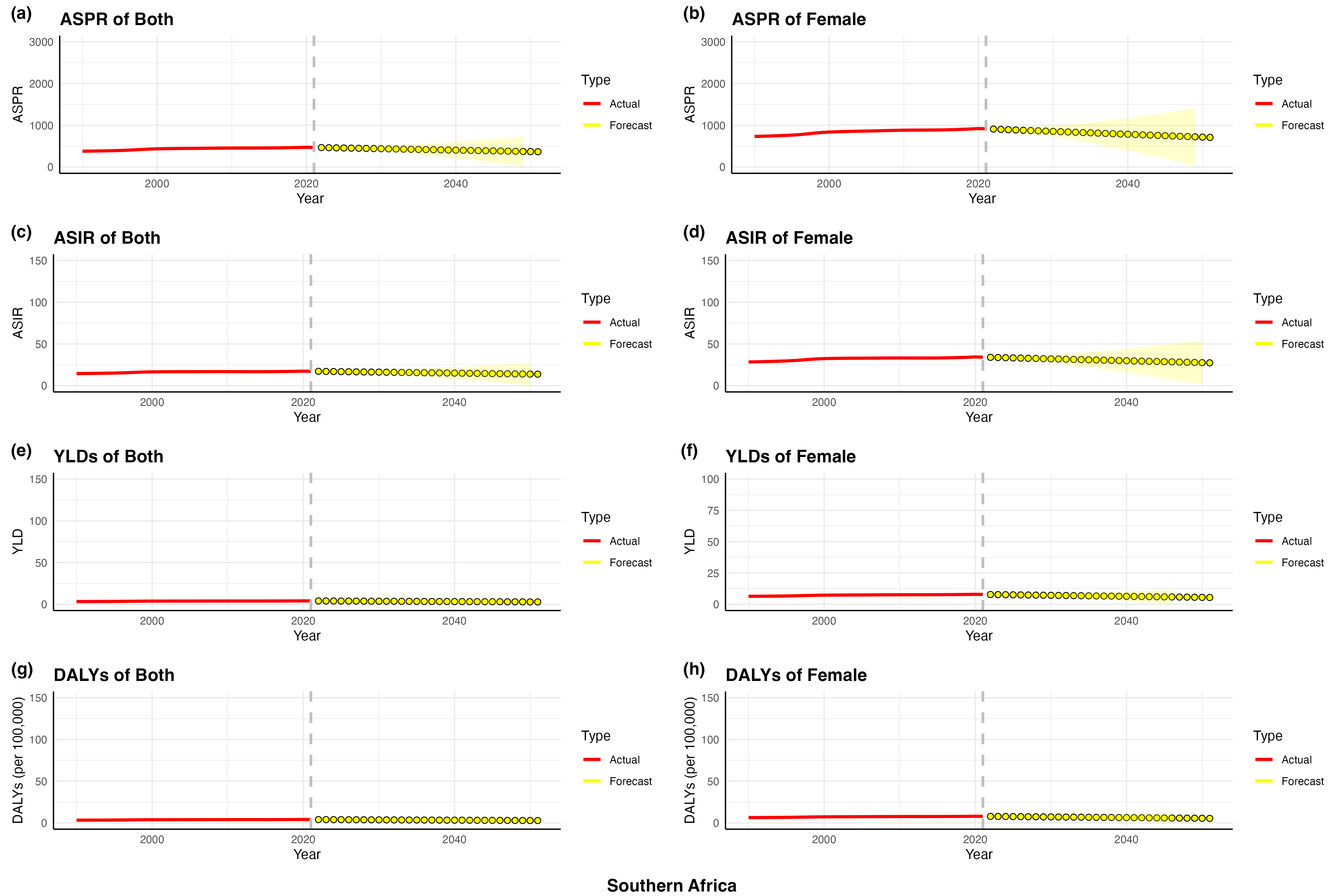

Supplement: Supplementary file 2 [file Supplementaryfile1.zip › Document/Document8-2/S 27/PCOS ARIMA Southern Africa.png]

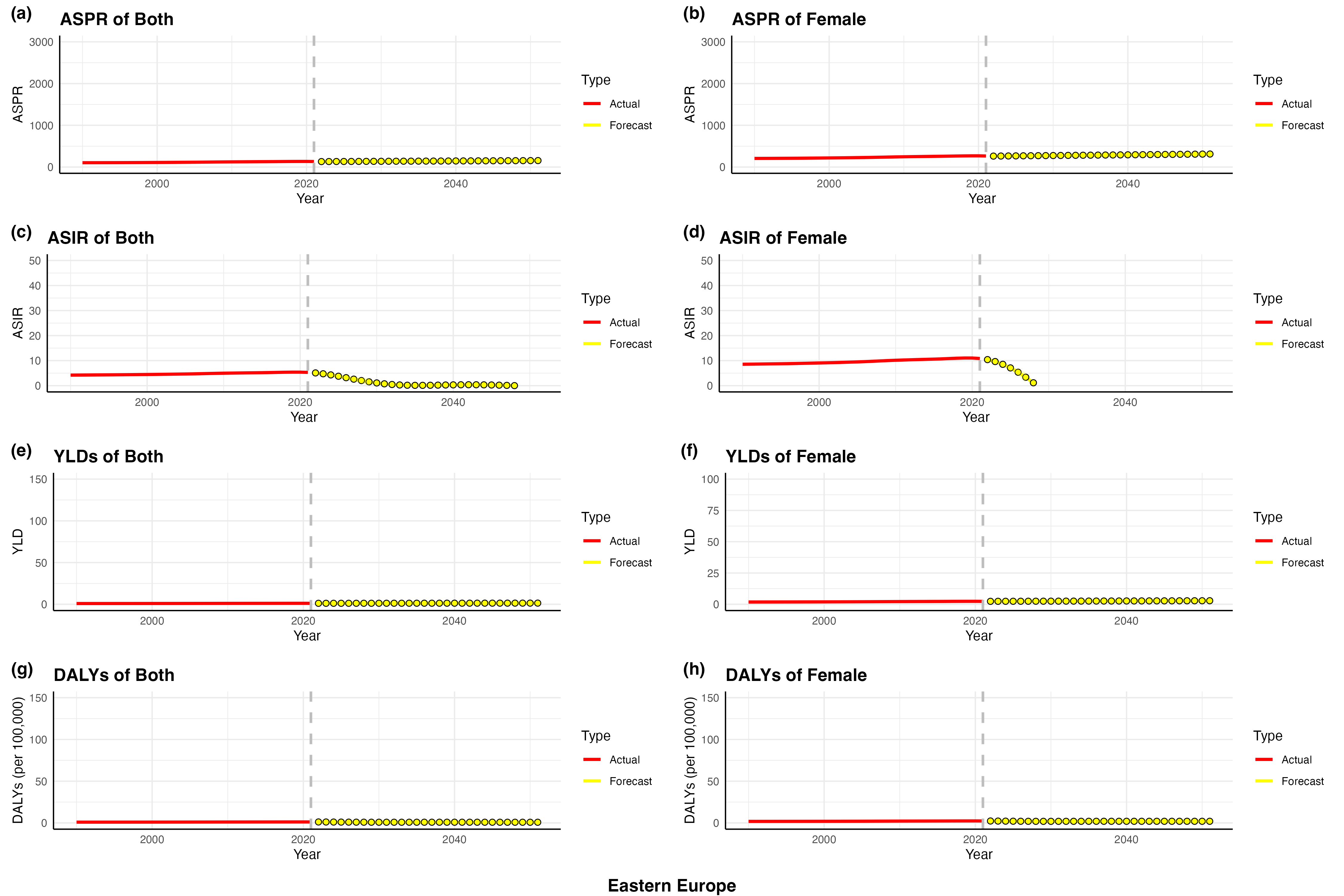

Supplement: Supplementary file 2 [file Supplementaryfile1.zip › Document/Document8-2/S 27/PCOS ARIMA Eastern Europe.png]

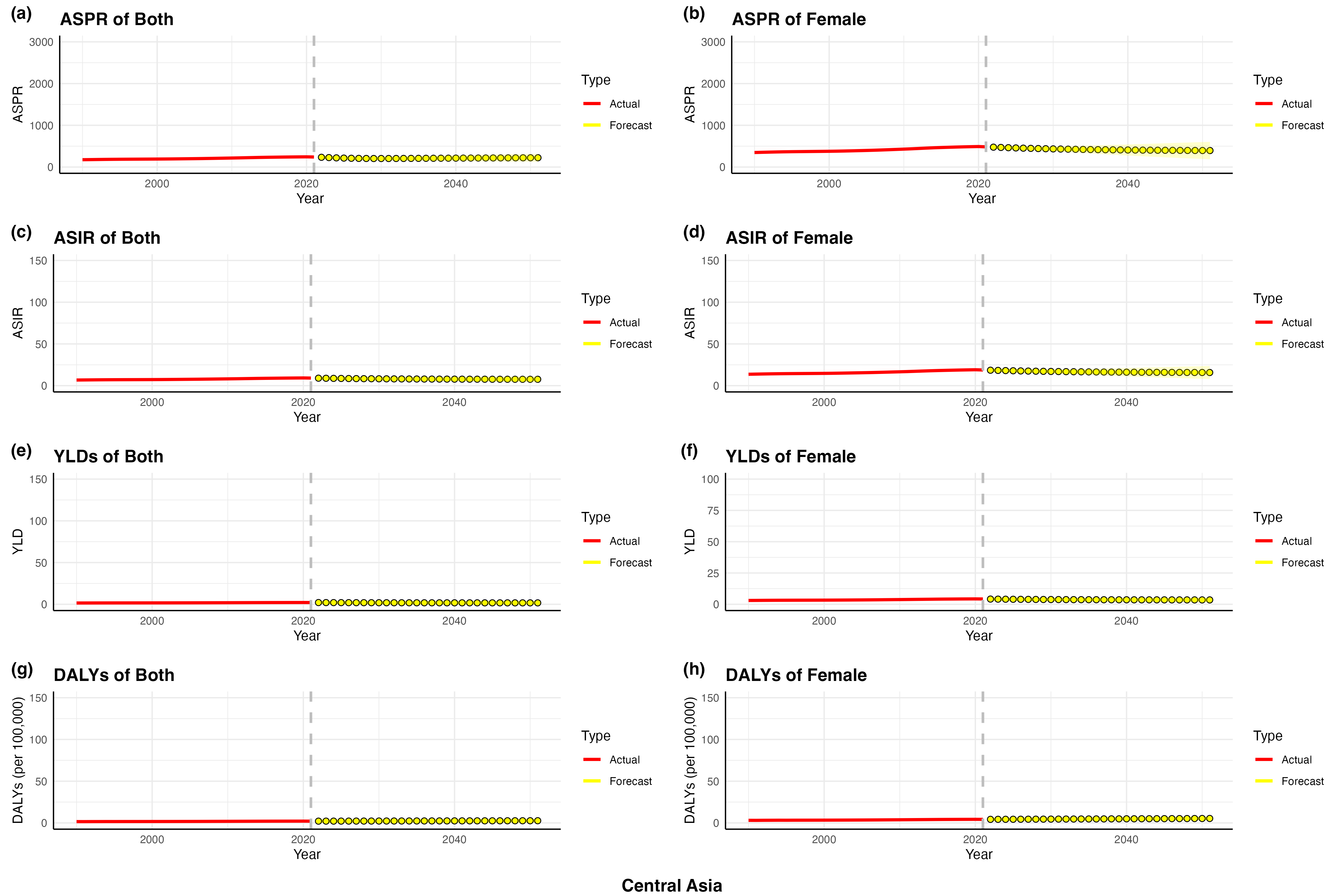

Supplement: Supplementary file 2 [file Supplementaryfile1.zip › Document/Document8-2/S 27/PCOS ARIMA Central Asia.png]

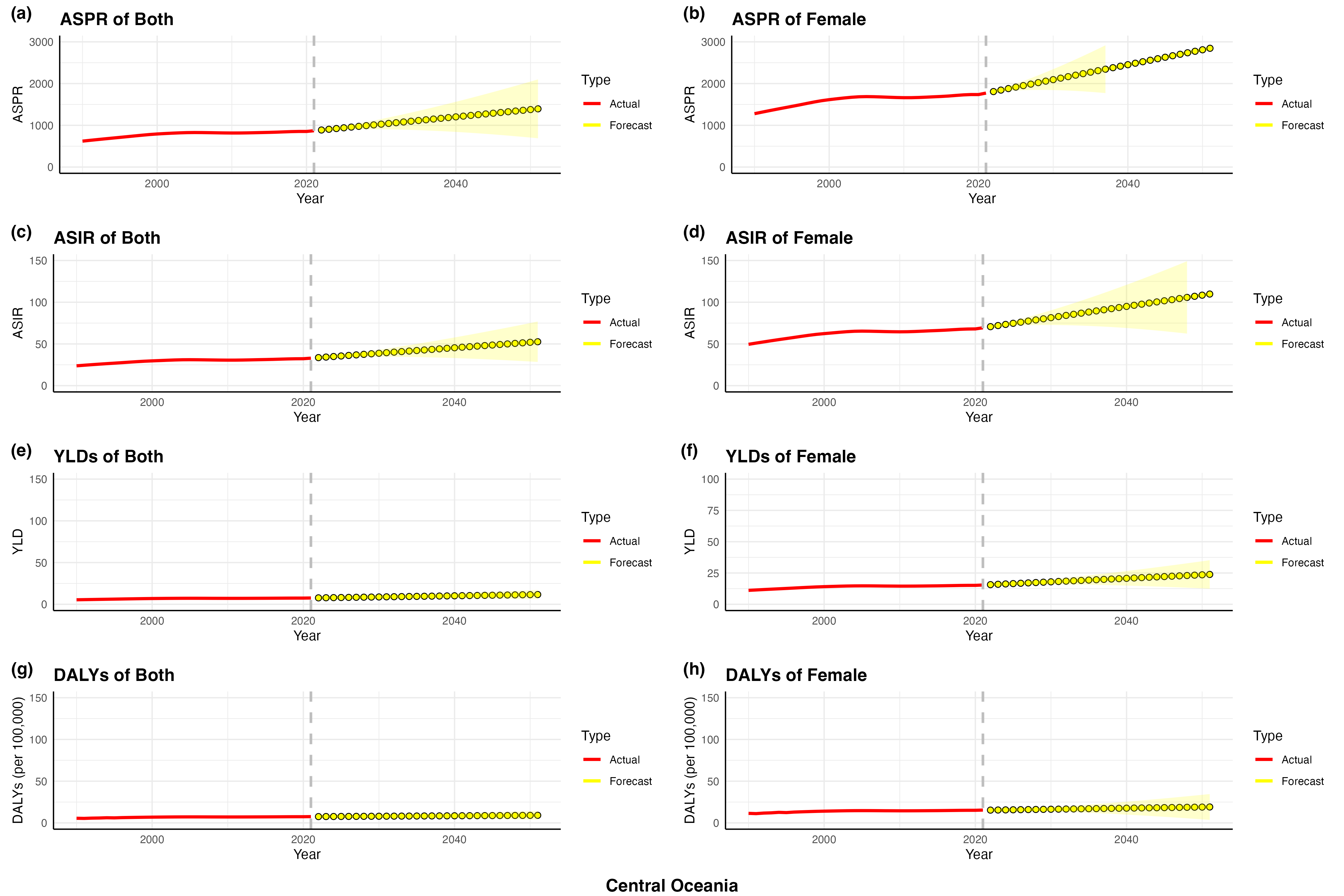

Supplement: Supplementary file 2 [file Supplementaryfile1.zip › Document/Document8-2/S 27/PCOS ARIMA Central Oceania.png]

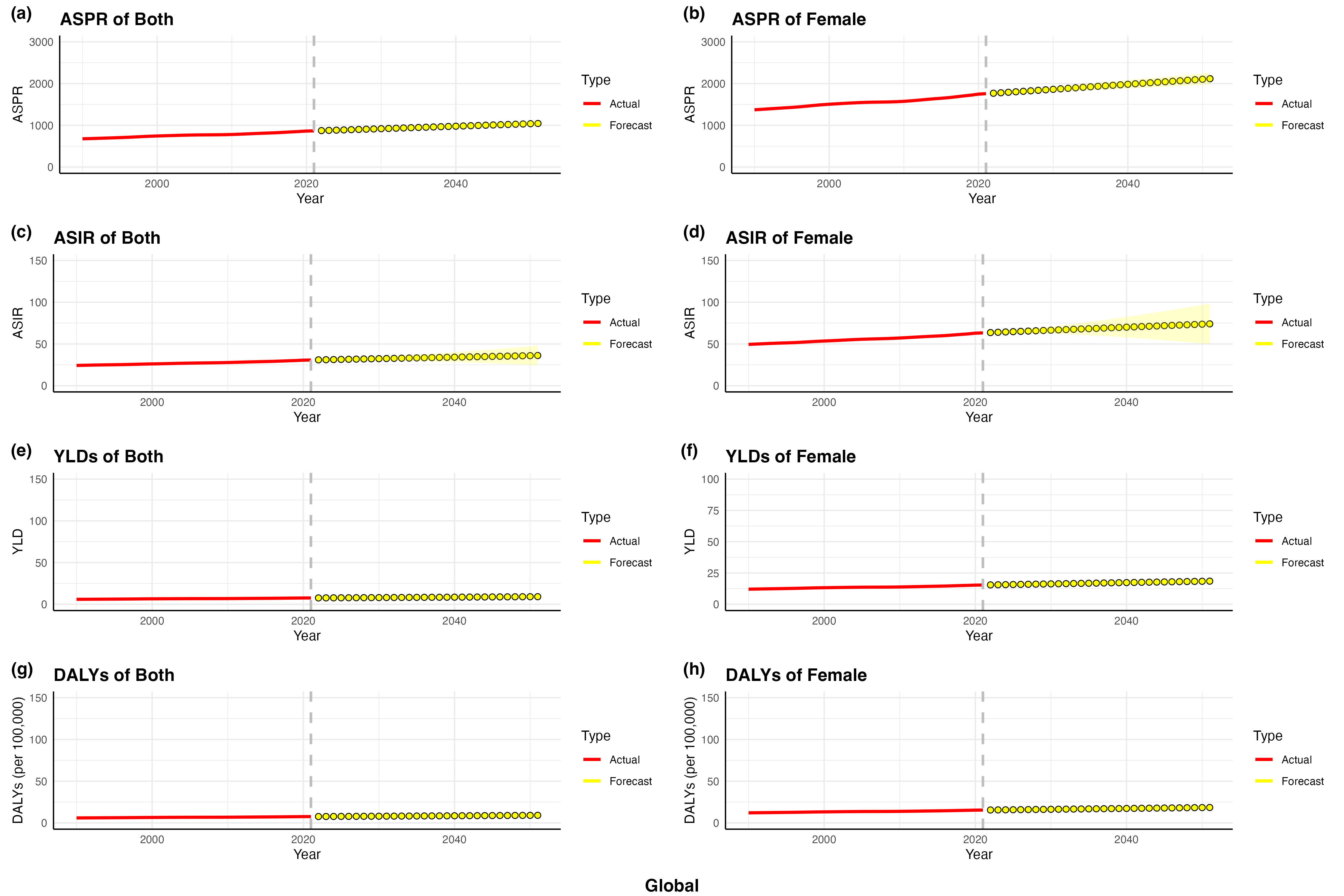

Supplement: Supplementary file 2 [file Supplementaryfile1.zip › Document/Document8-2/S 27/PCOS ARIMA Global.png]

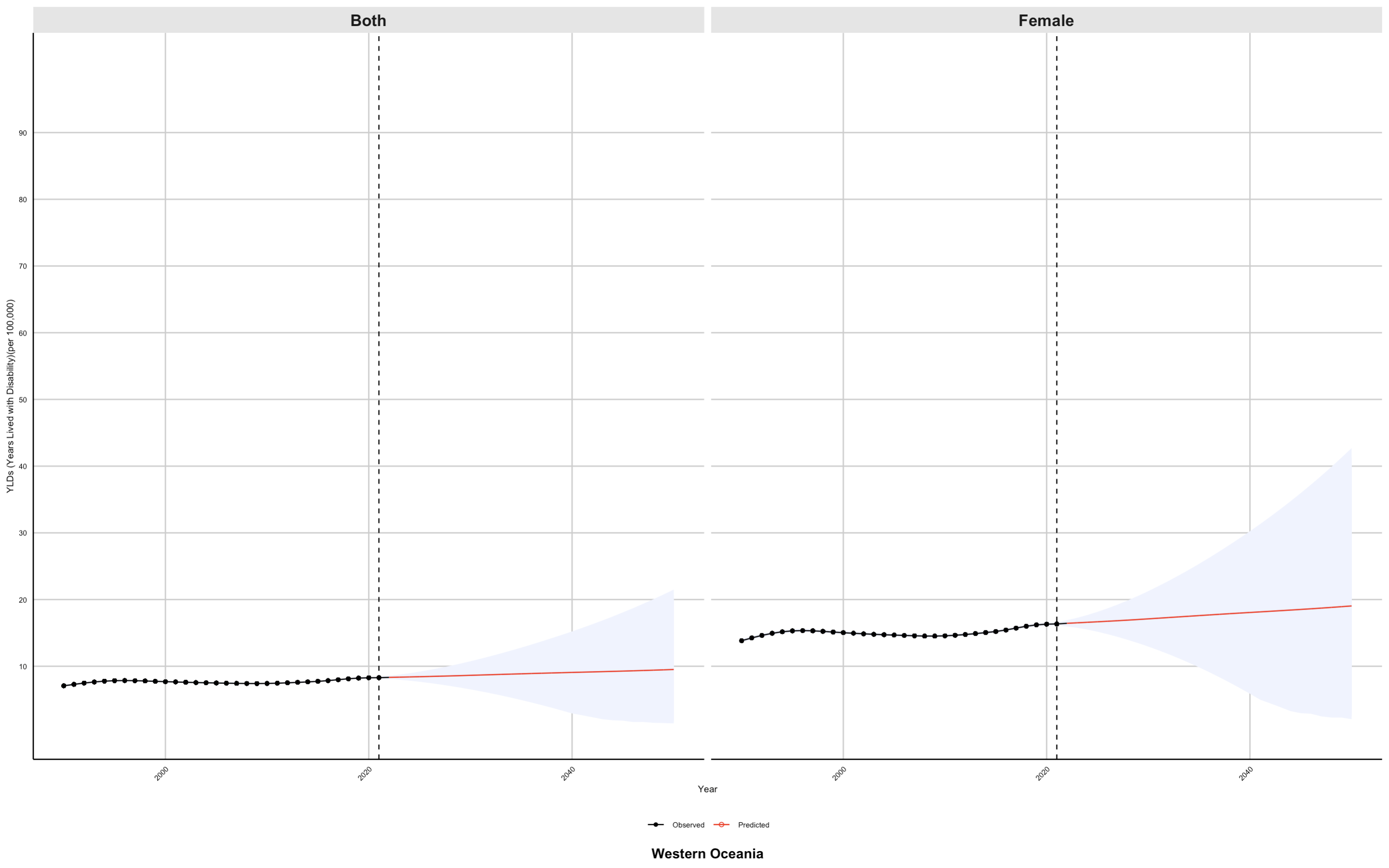

Supplement: Supplementary file 2 [file Supplementaryfile1.zip › Document/Document8-2/S 26/Western OceaniaBAPC YLDs.png]

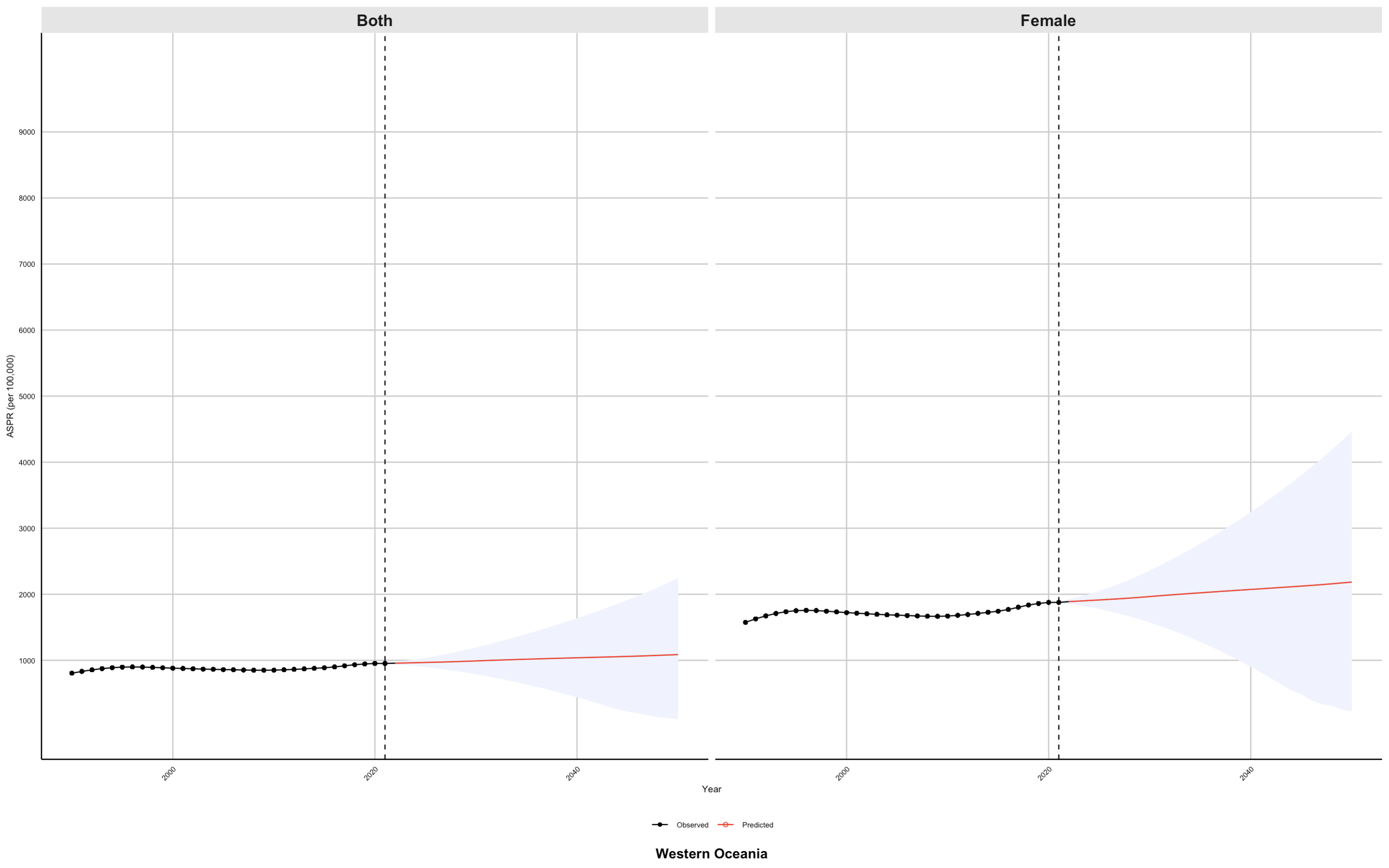

Supplement: Supplementary file 2 [file Supplementaryfile1.zip › Document/Document8-2/S 26/Western OceaniaBAPC ASPR.png]

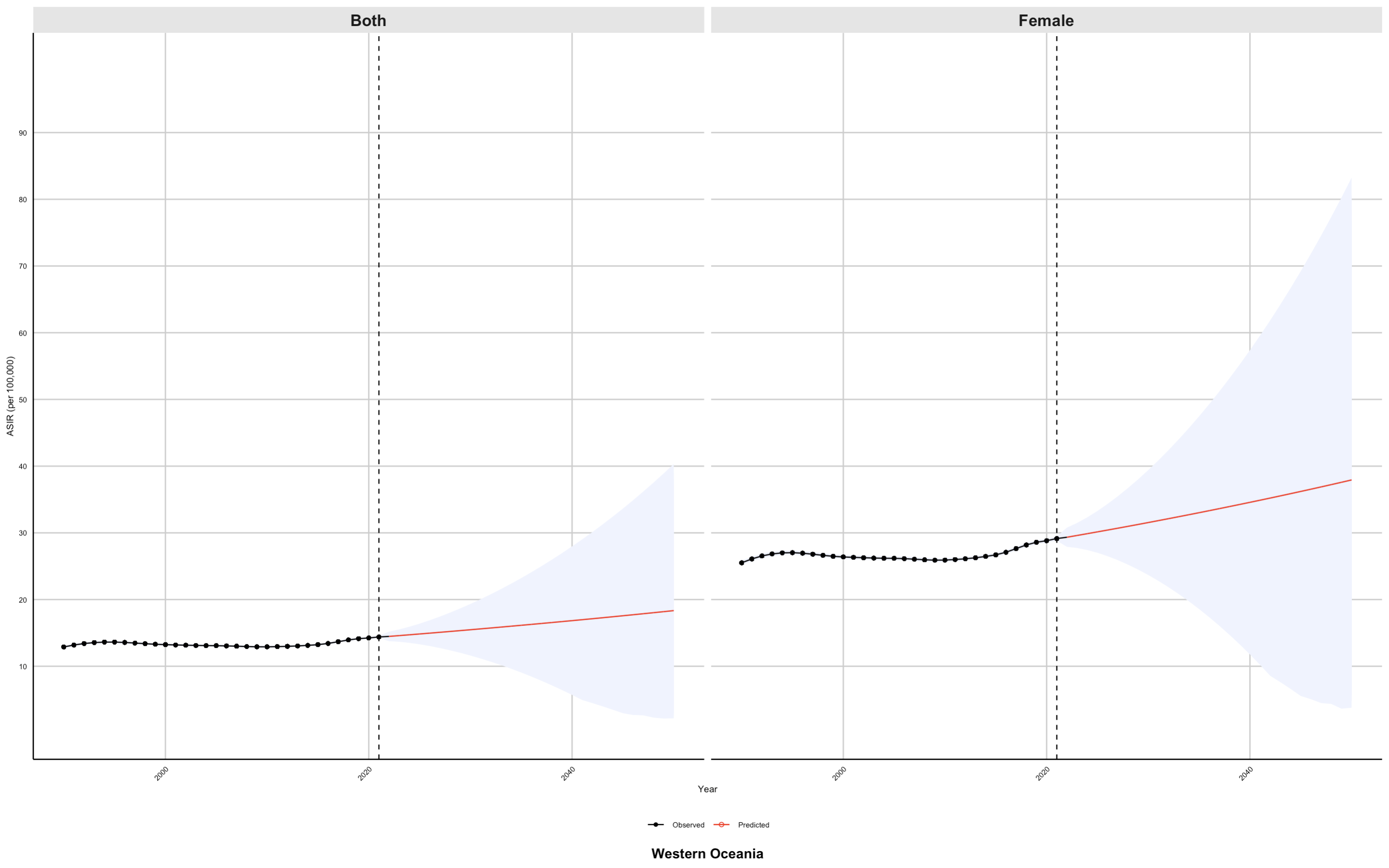

Supplement: Supplementary file 2 [file Supplementaryfile1.zip › Document/Document8-2/S 26/Western OceaniaBAPC ASIR.png]

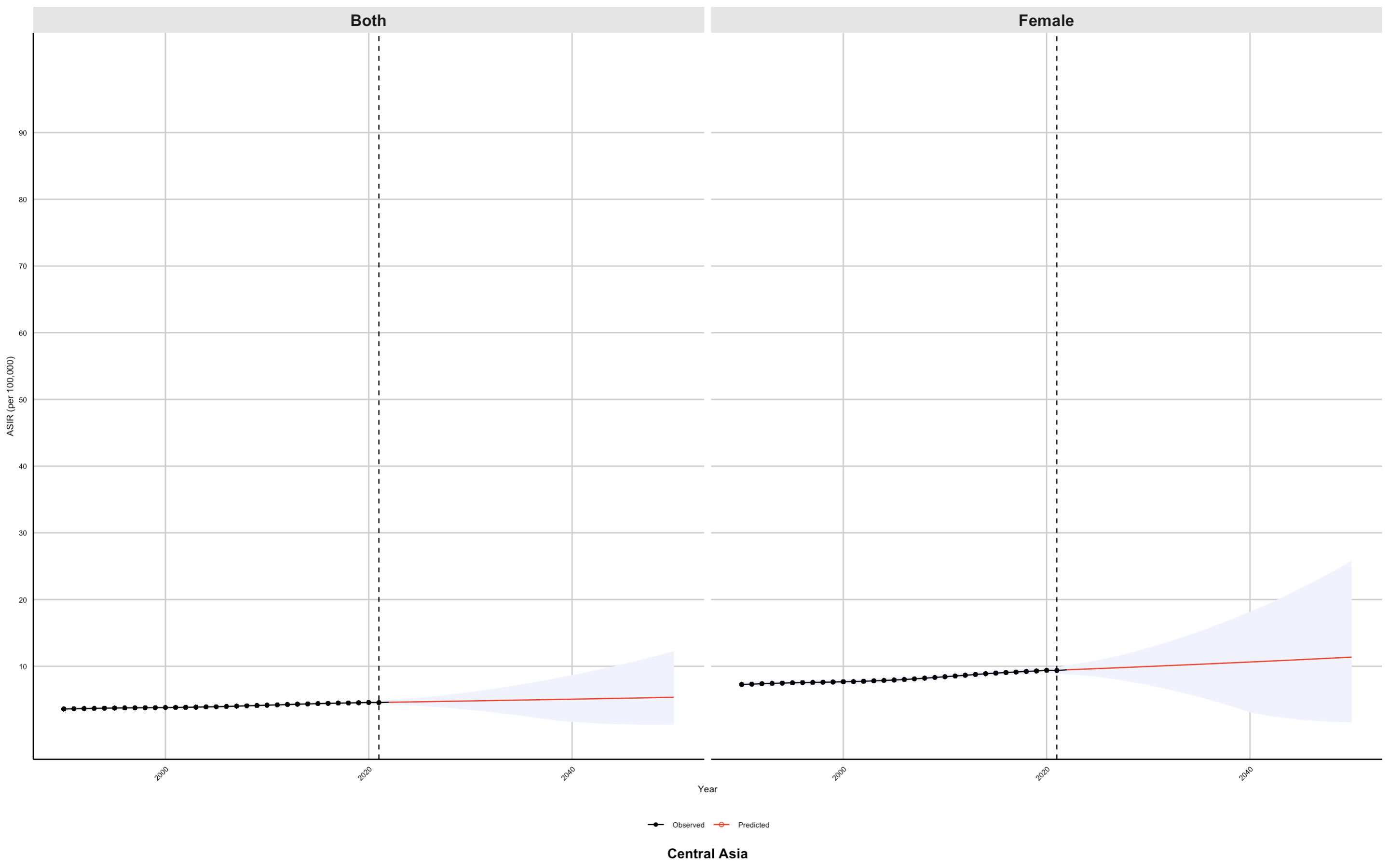

Supplement: Supplementary file 2 [file Supplementaryfile1.zip › Document/Document8-2/S 26/Central AsiaBAPC ASIR.png]

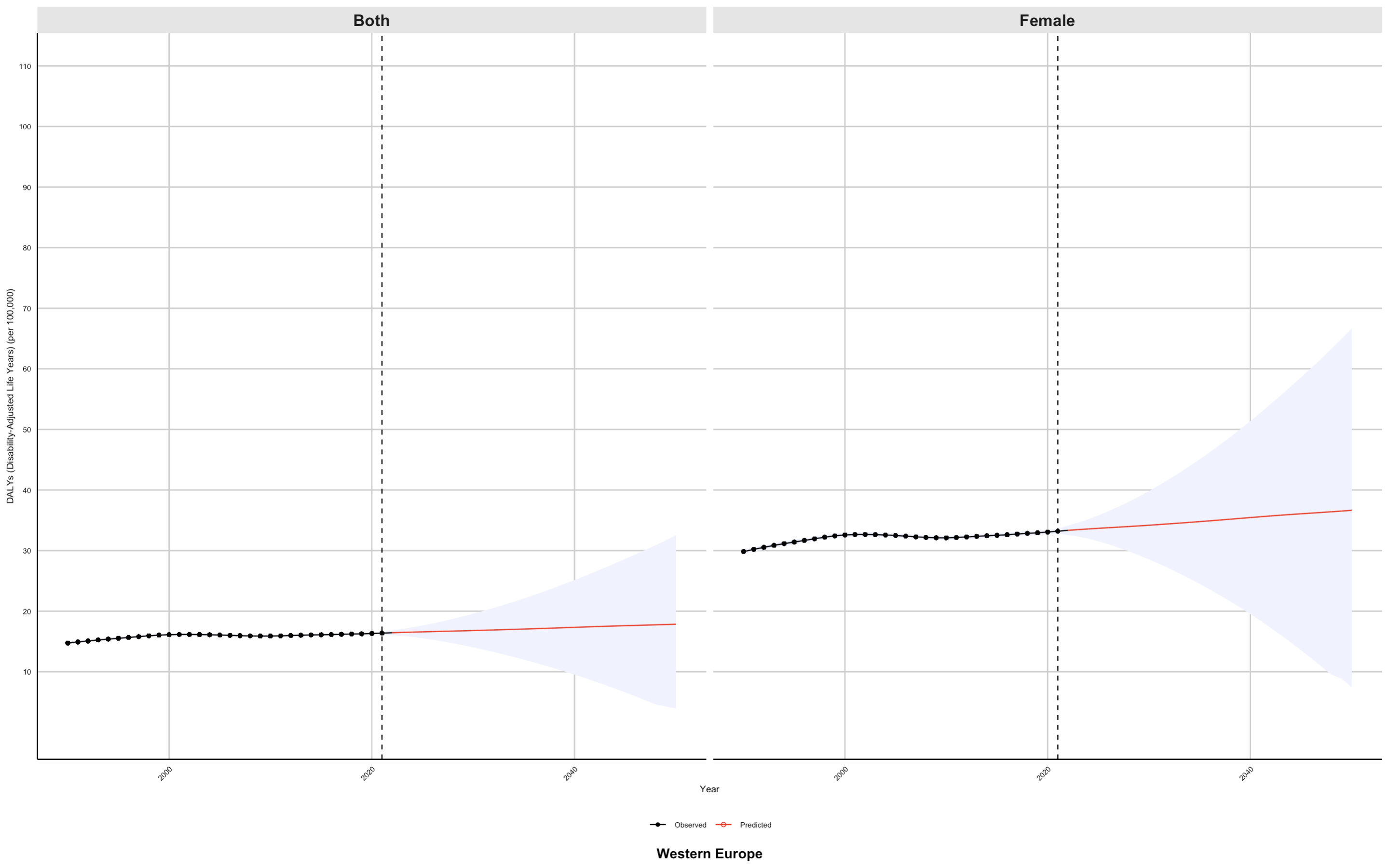

Supplement: Supplementary file 2 [file Supplementaryfile1.zip › Document/Document8-2/S 26/Western EuropeBAPC DALYs.png]

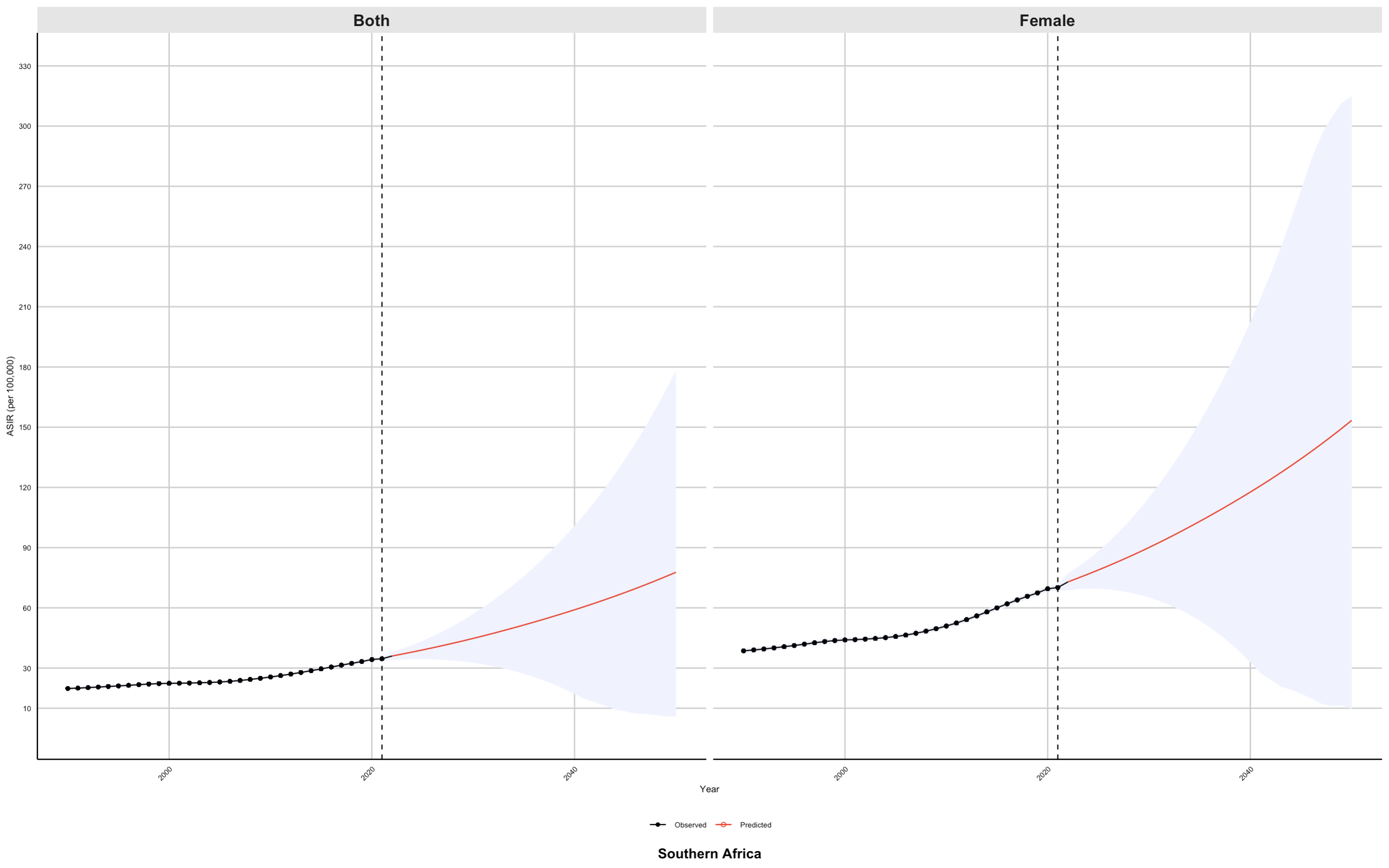

Supplement: Supplementary file 2 [file Supplementaryfile1.zip › Document/Document8-2/S 26/Southern AfricaBAPC ASIR.png]

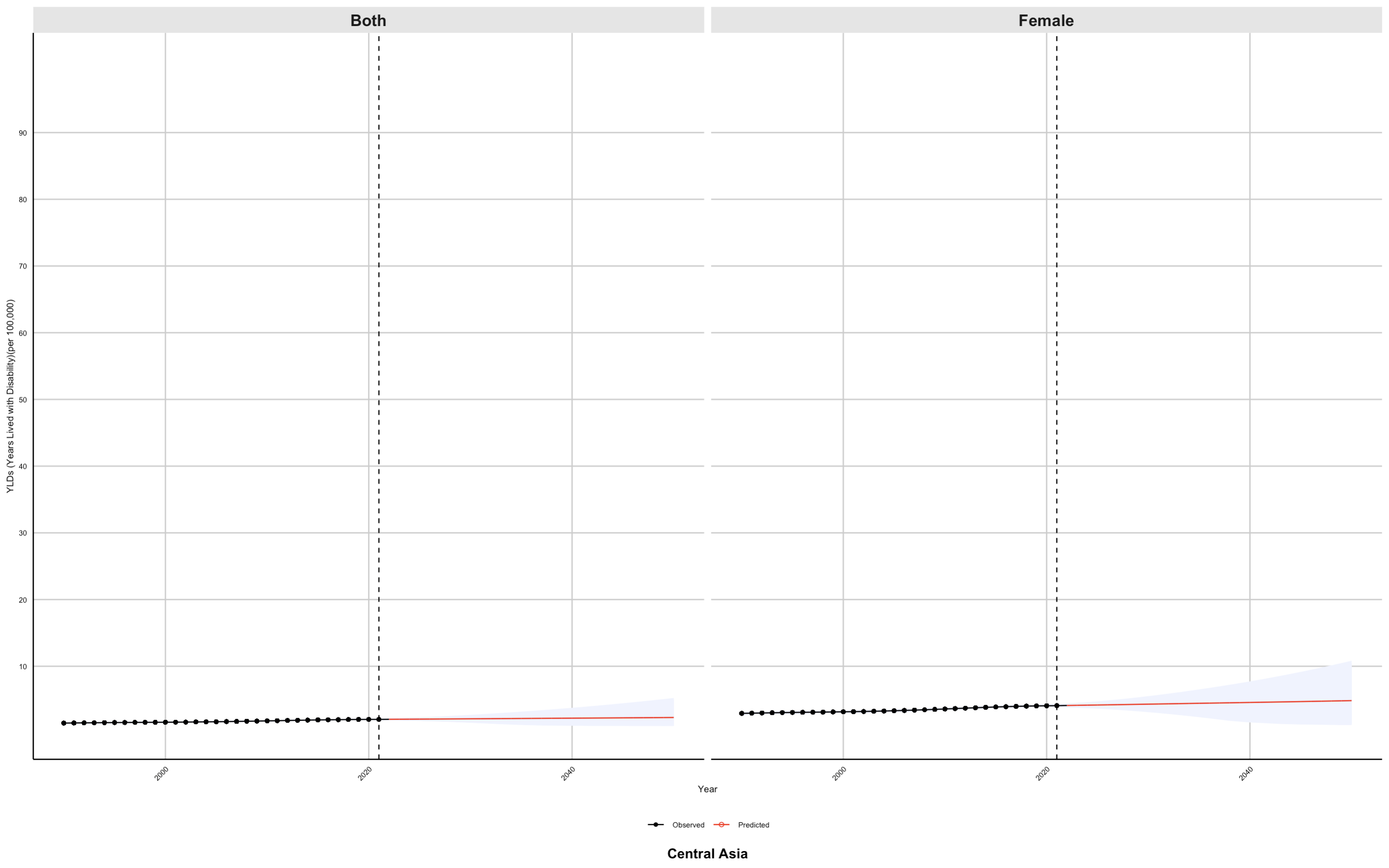

Supplement: Supplementary file 2 [file Supplementaryfile1.zip › Document/Document8-2/S 26/Central AsiaBAPC YLDs.png]

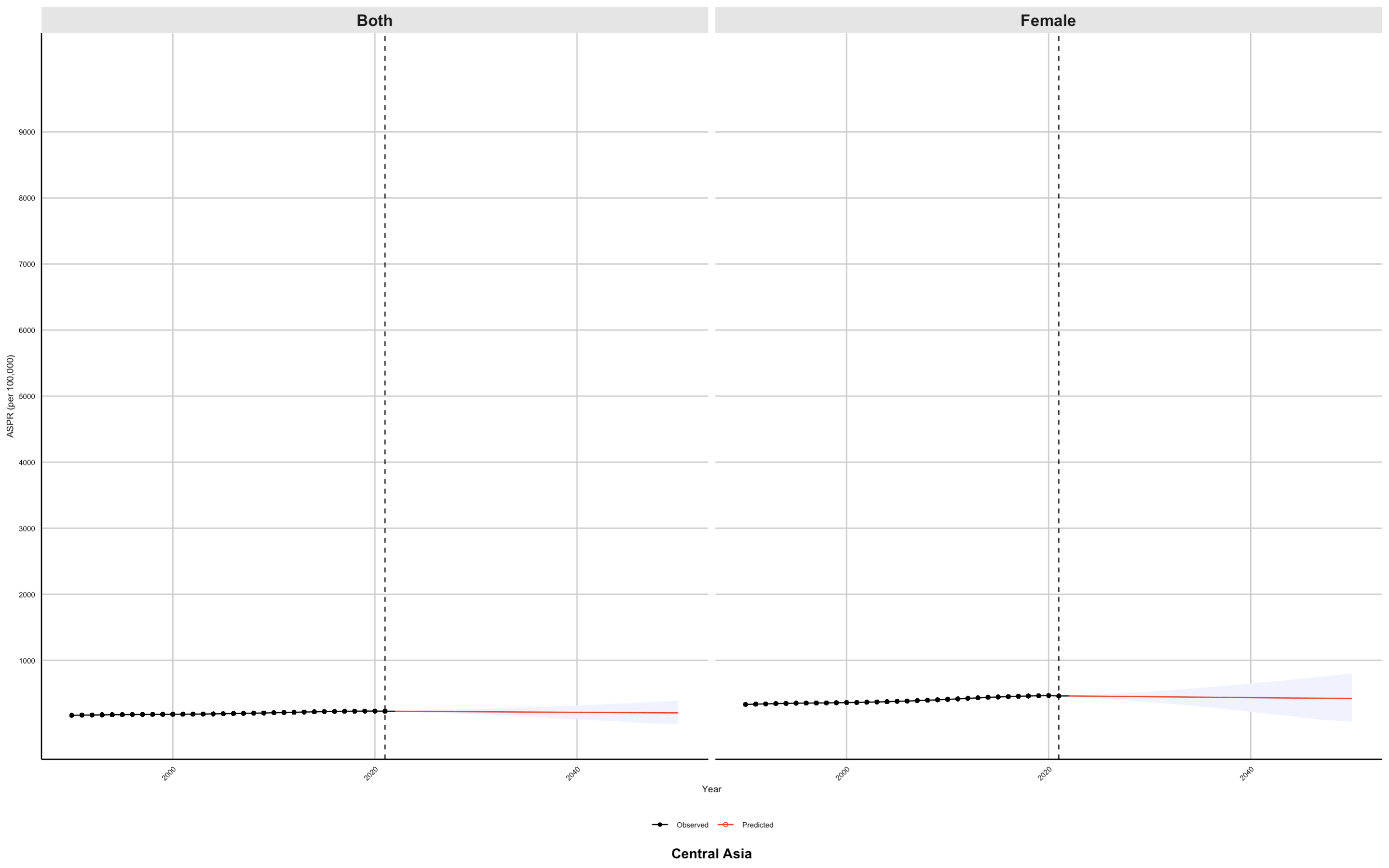

Supplement: Supplementary file 2 [file Supplementaryfile1.zip › Document/Document8-2/S 26/Central AsiaBAPC ASPR.png]

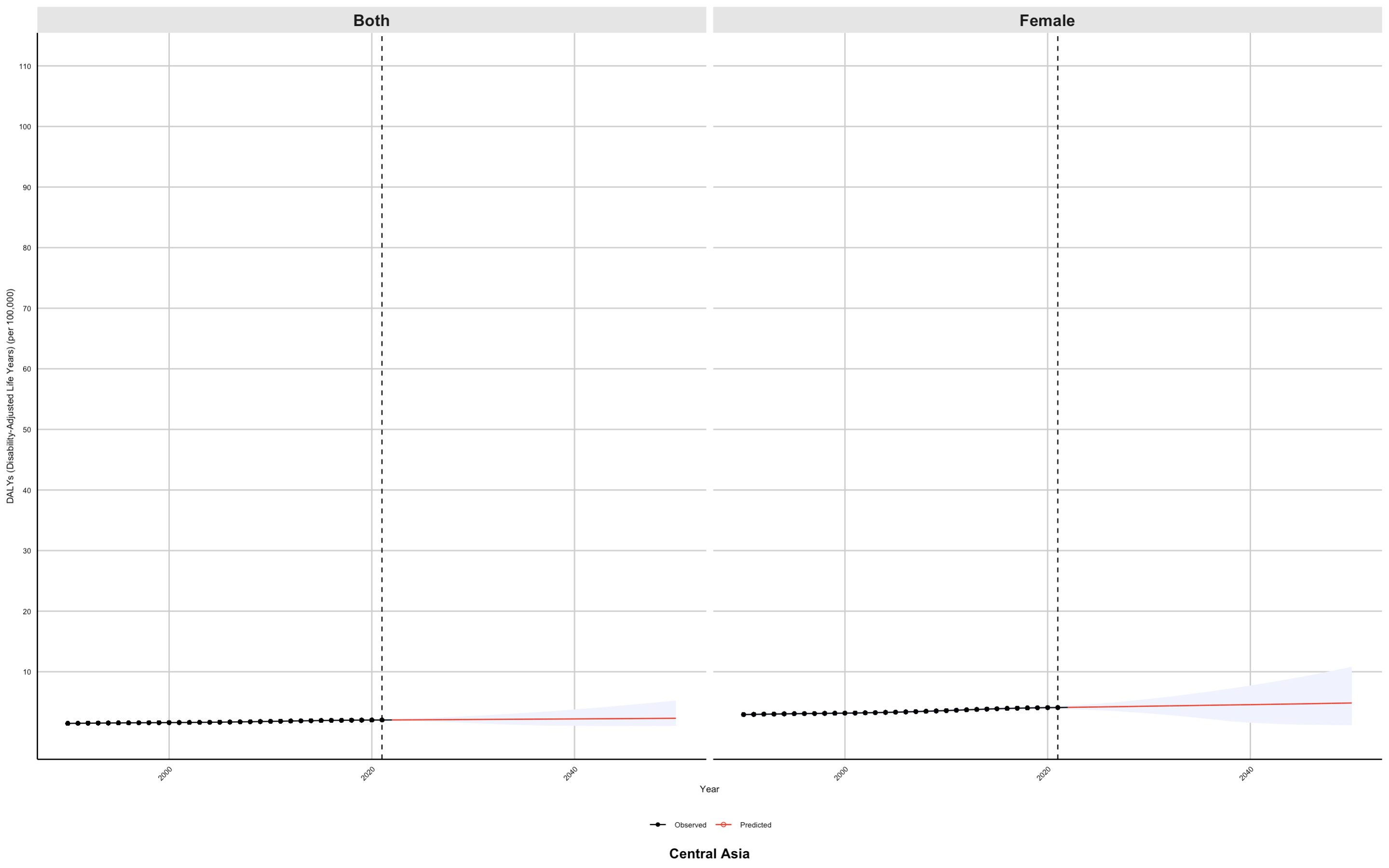

Supplement: Supplementary file 2 [file Supplementaryfile1.zip › Document/Document8-2/S 26/Central AsiaBAPC DALYs.png]

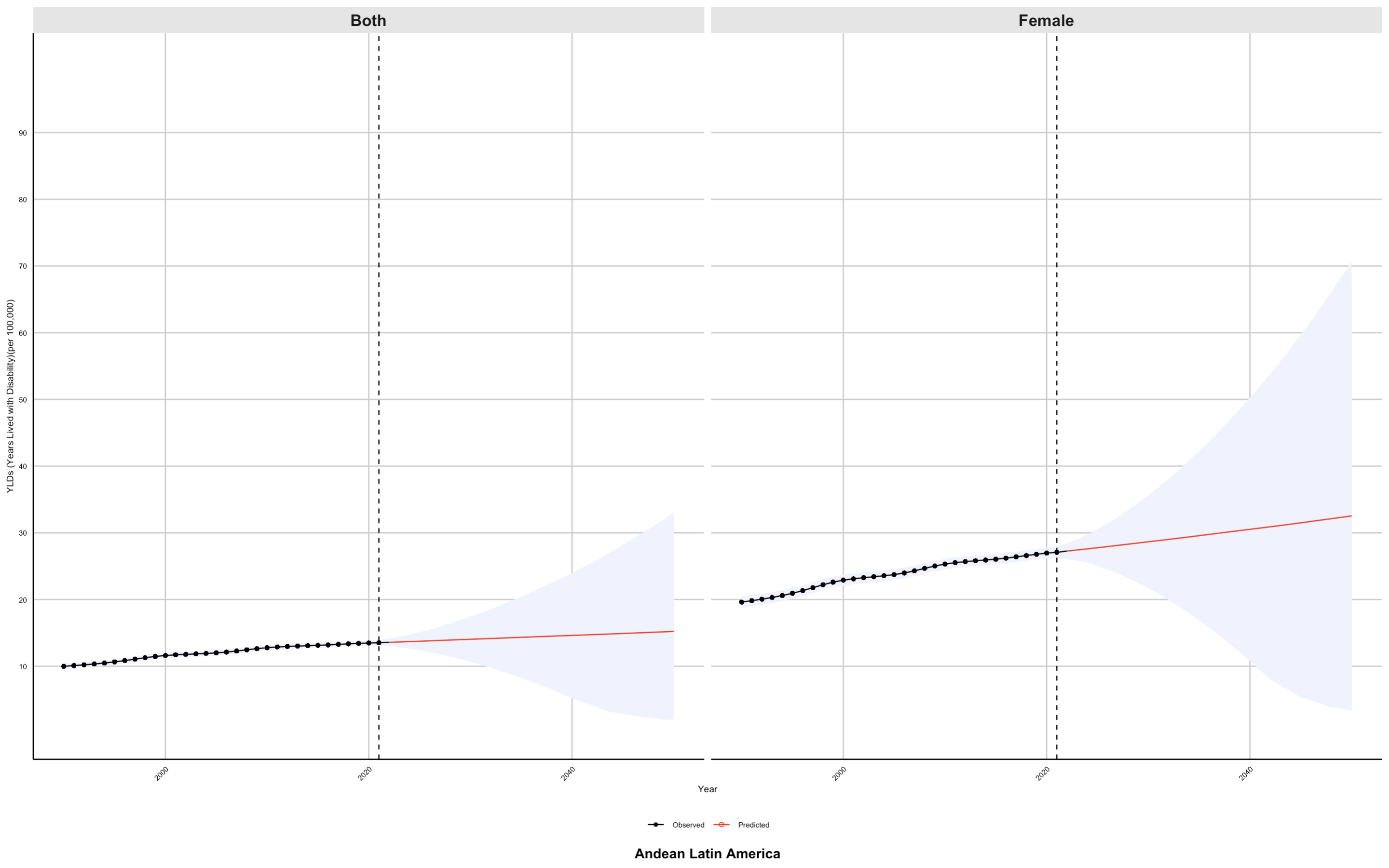

Supplement: Supplementary file 2 [file Supplementaryfile1.zip › Document/Document8-2/S 26/Andean Latin America BAPC YLDS.png]

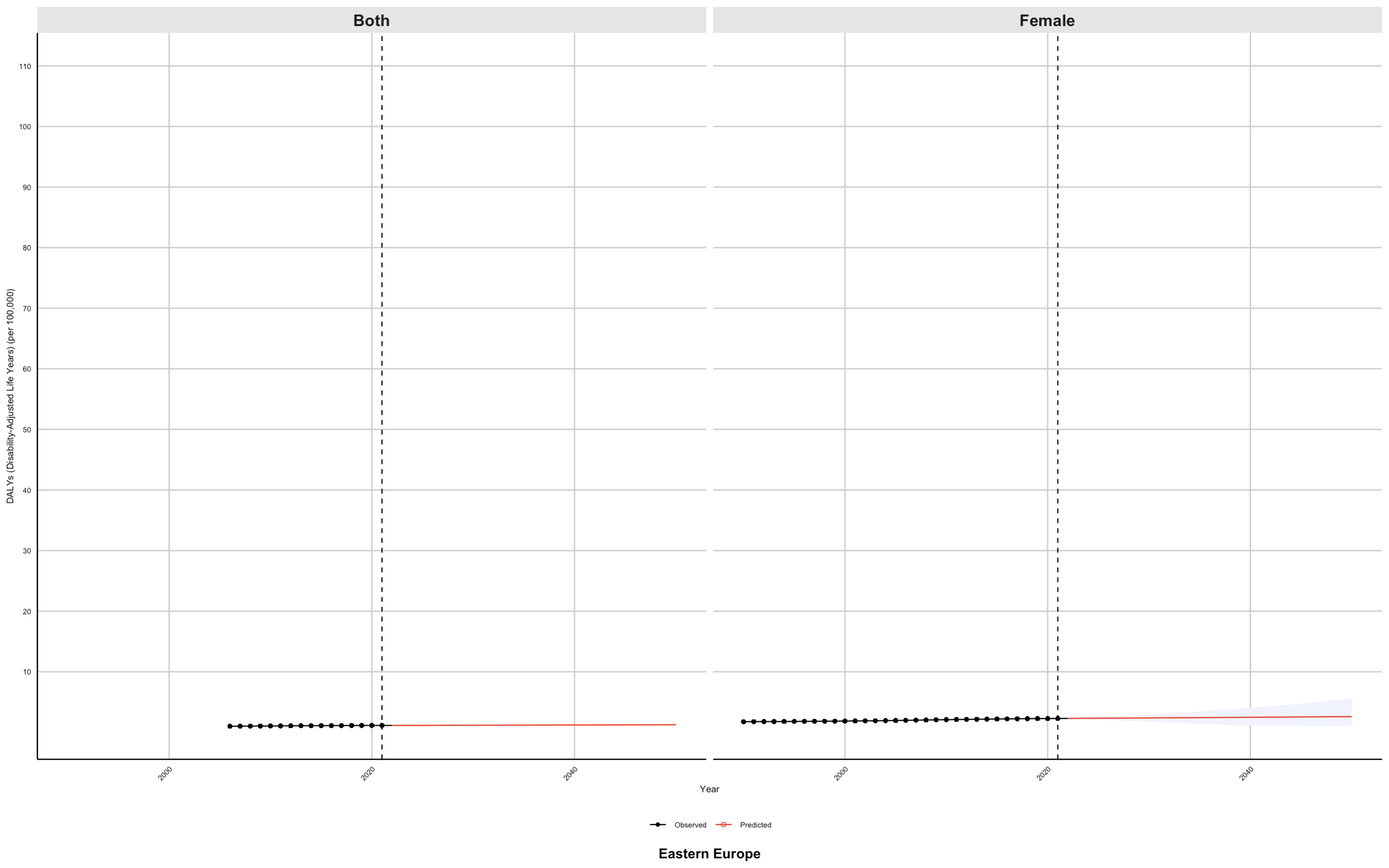

Supplement: Supplementary file 2 [file Supplementaryfile1.zip › Document/Document8-2/S 26/Eastern EuropeBAPC DALys.png]

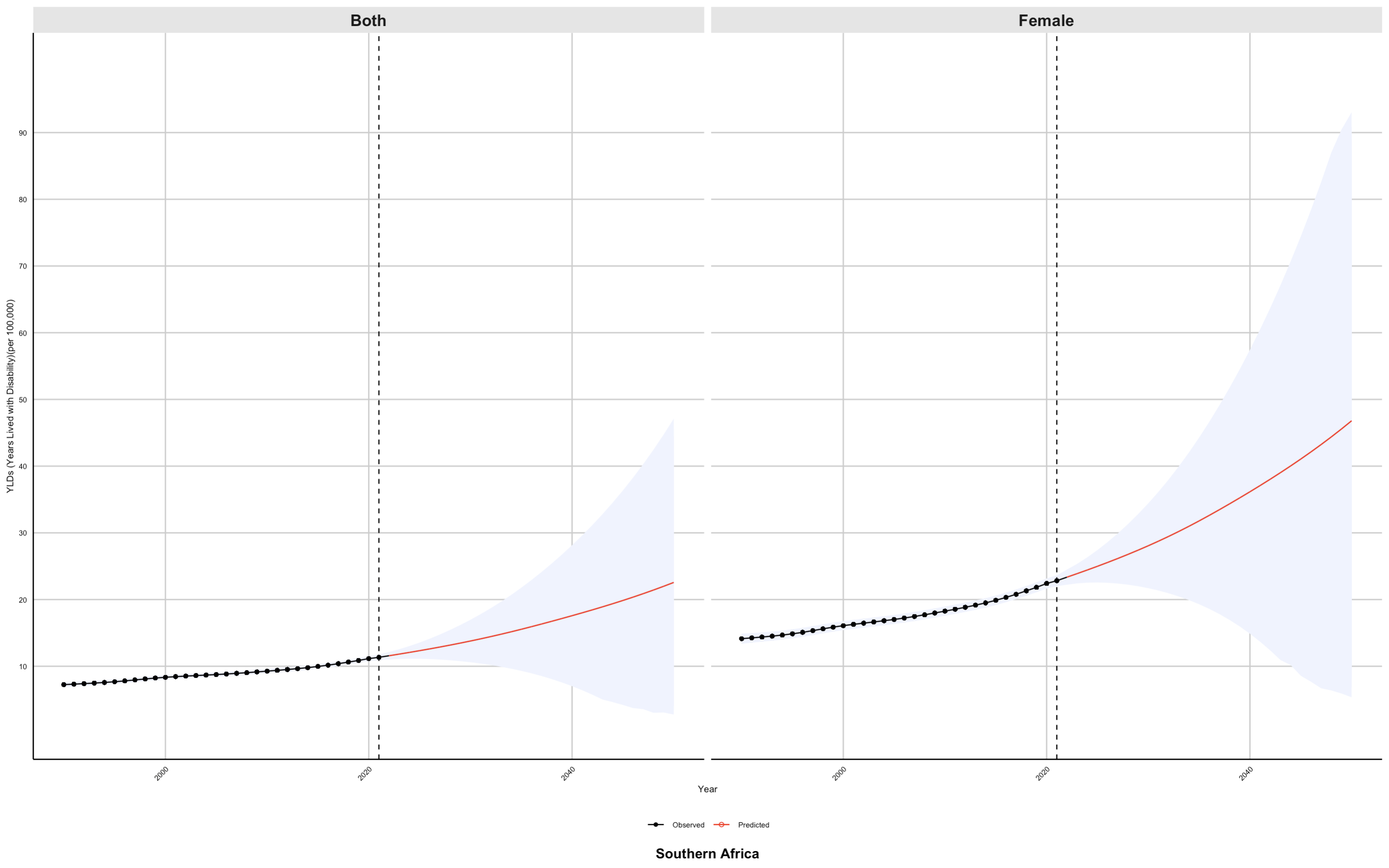

Supplement: Supplementary file 2 [file Supplementaryfile1.zip › Document/Document8-2/S 26/Southern Africa BAPC YLDS.png]

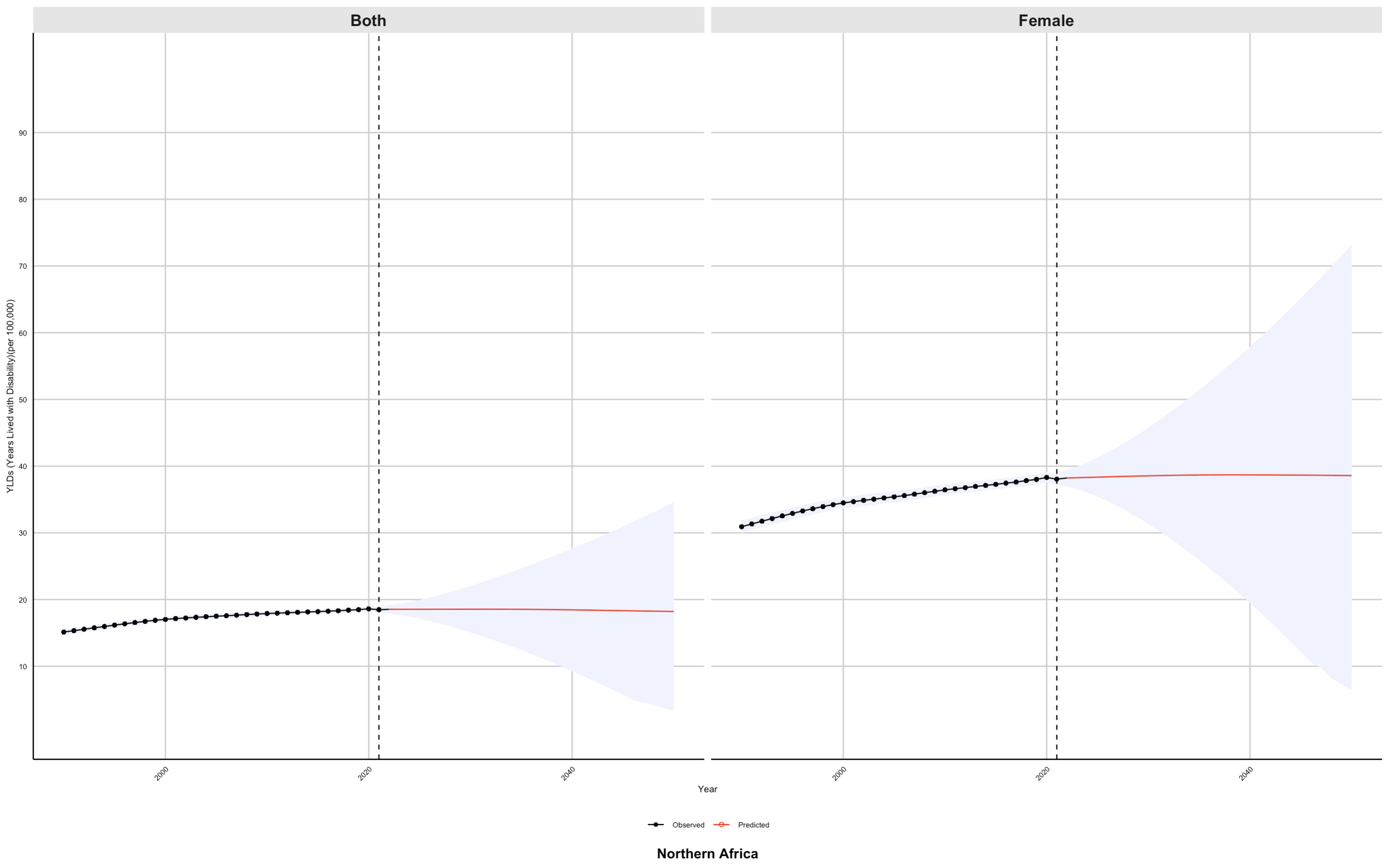

Supplement: Supplementary file 2 [file Supplementaryfile1.zip › Document/Document8-2/S 26/Northern Africa BAPC YLDs.png]

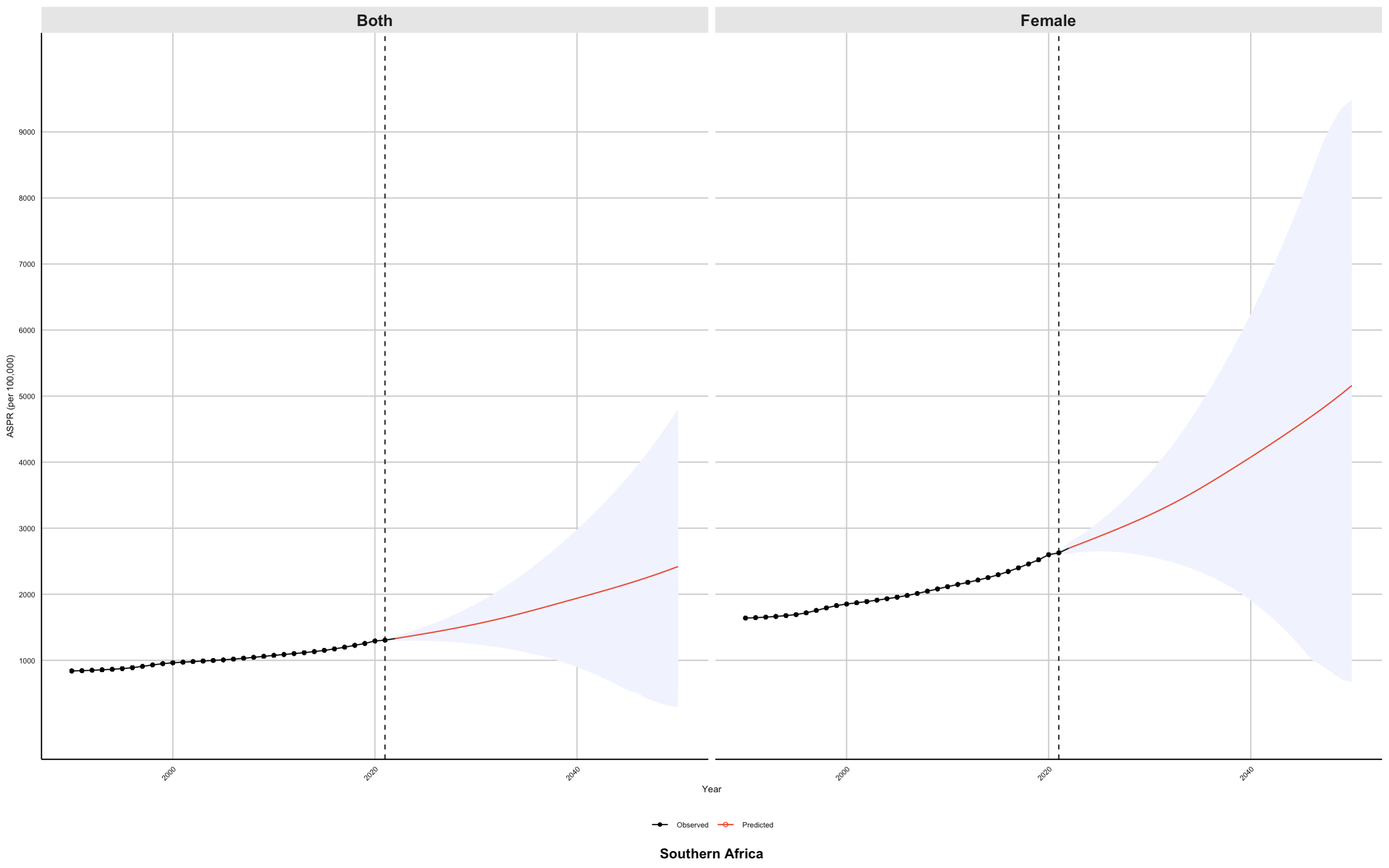

Supplement: Supplementary file 2 [file Supplementaryfile1.zip › Document/Document8-2/S 26/Southern Africa BAPC ASPR.png]

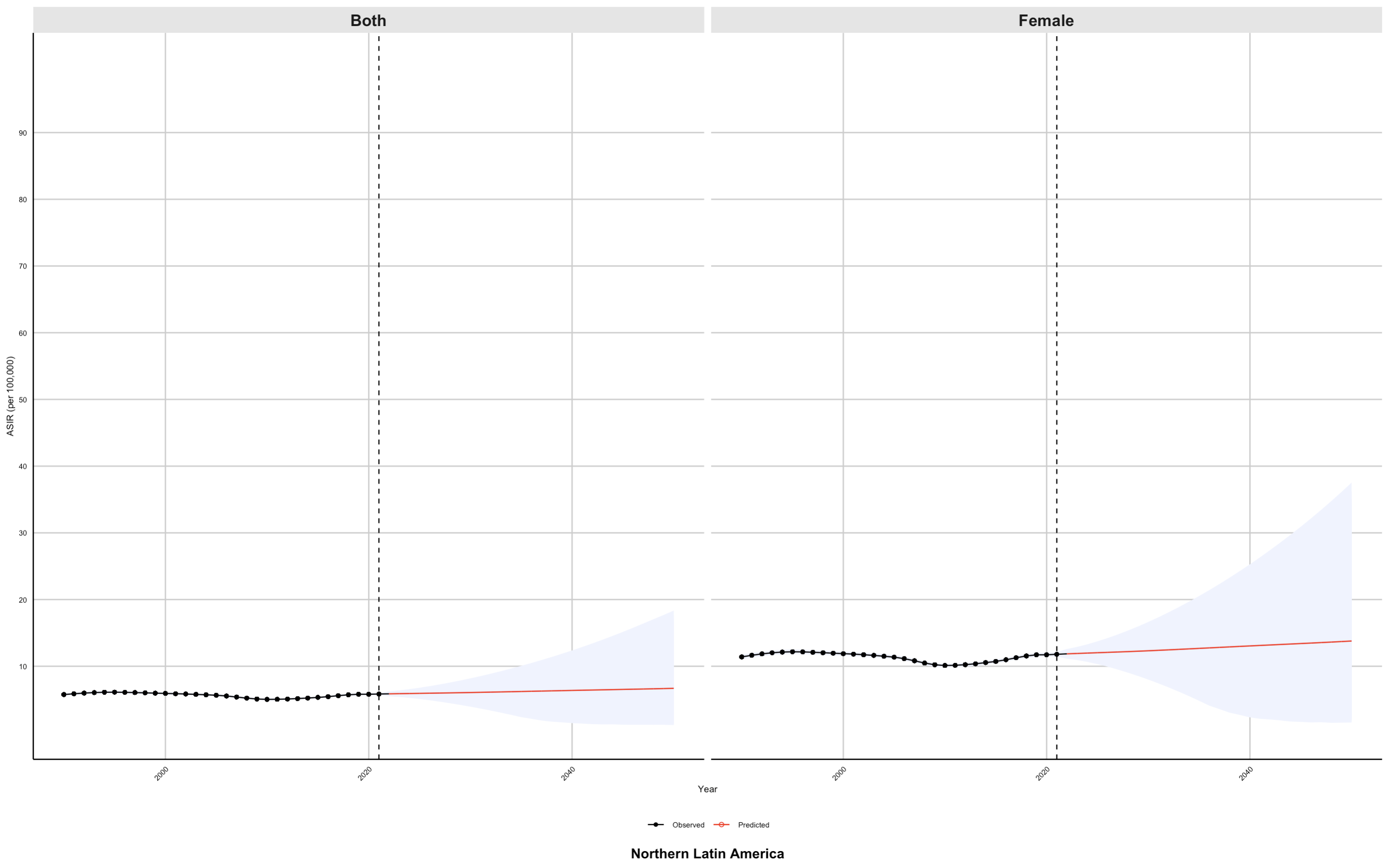

Supplement: Supplementary file 2 [file Supplementaryfile1.zip › Document/Document8-2/S 26/Northern Latin America BAPC ASIR.png]

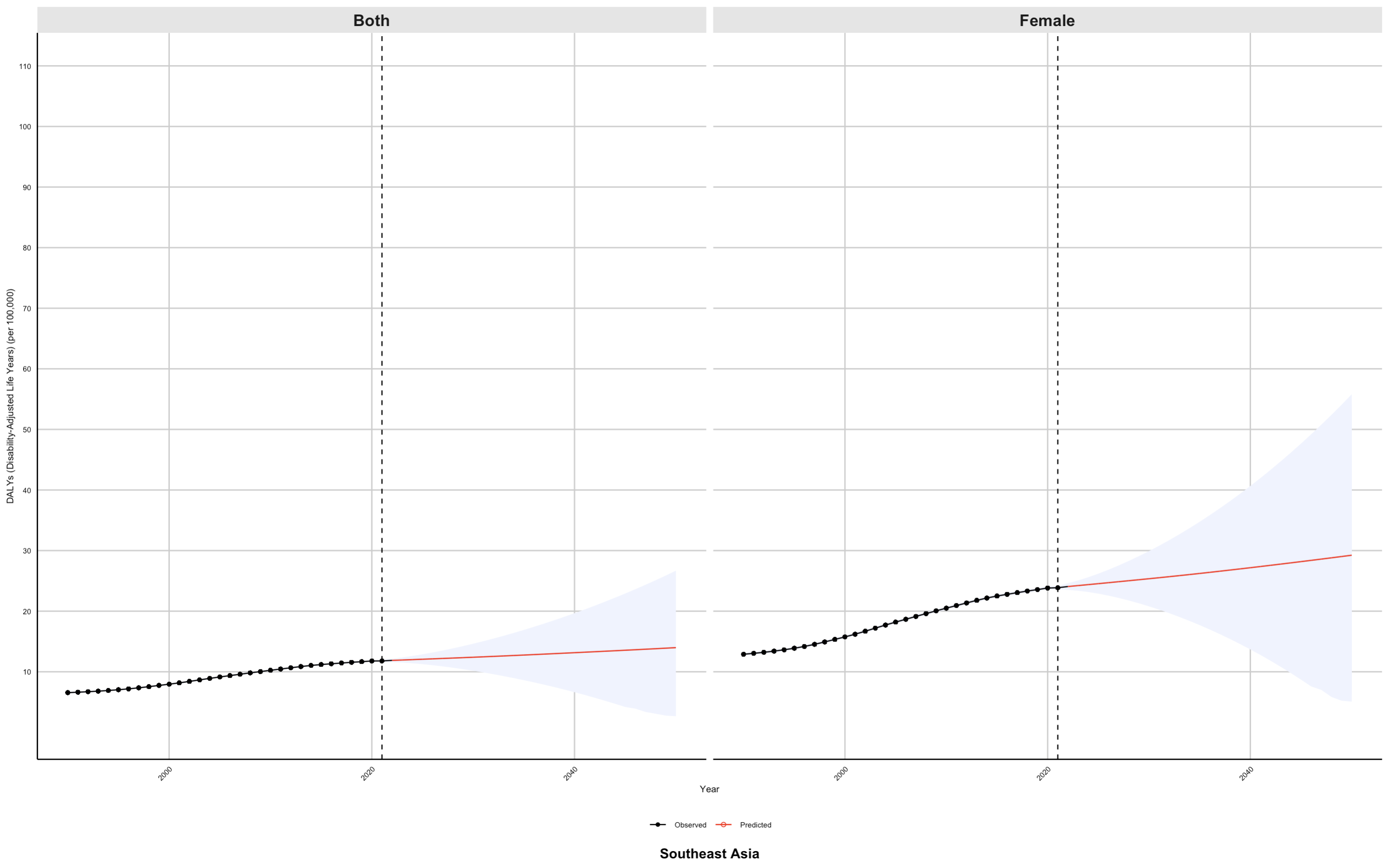

Supplement: Supplementary file 2 [file Supplementaryfile1.zip › Document/Document8-2/S 26/Southeast Asia BAPC DAlys.png]

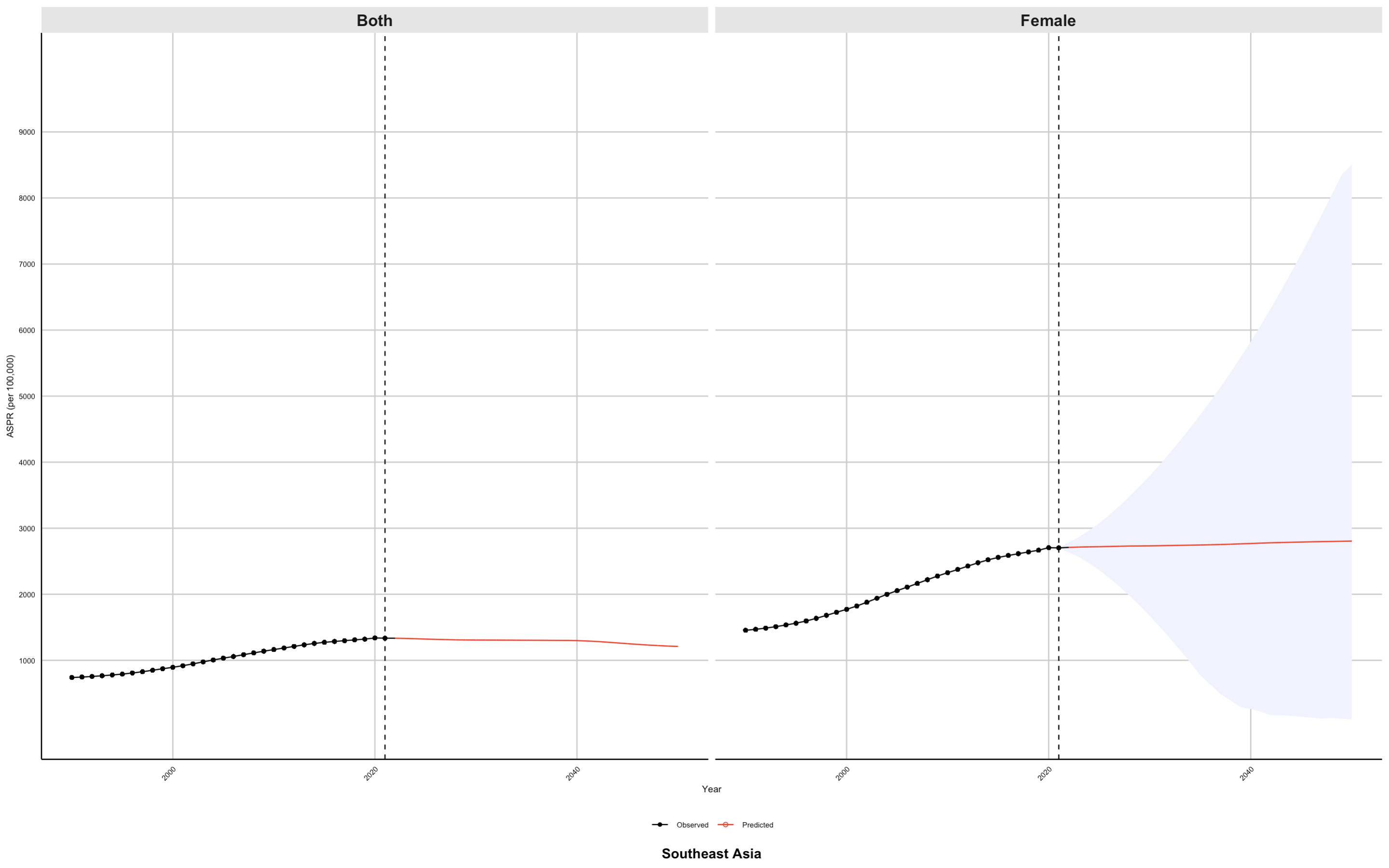

Supplement: Supplementary file 2 [file Supplementaryfile1.zip › Document/Document8-2/S 26/Southeast AsiaBAPC ASPR.png]

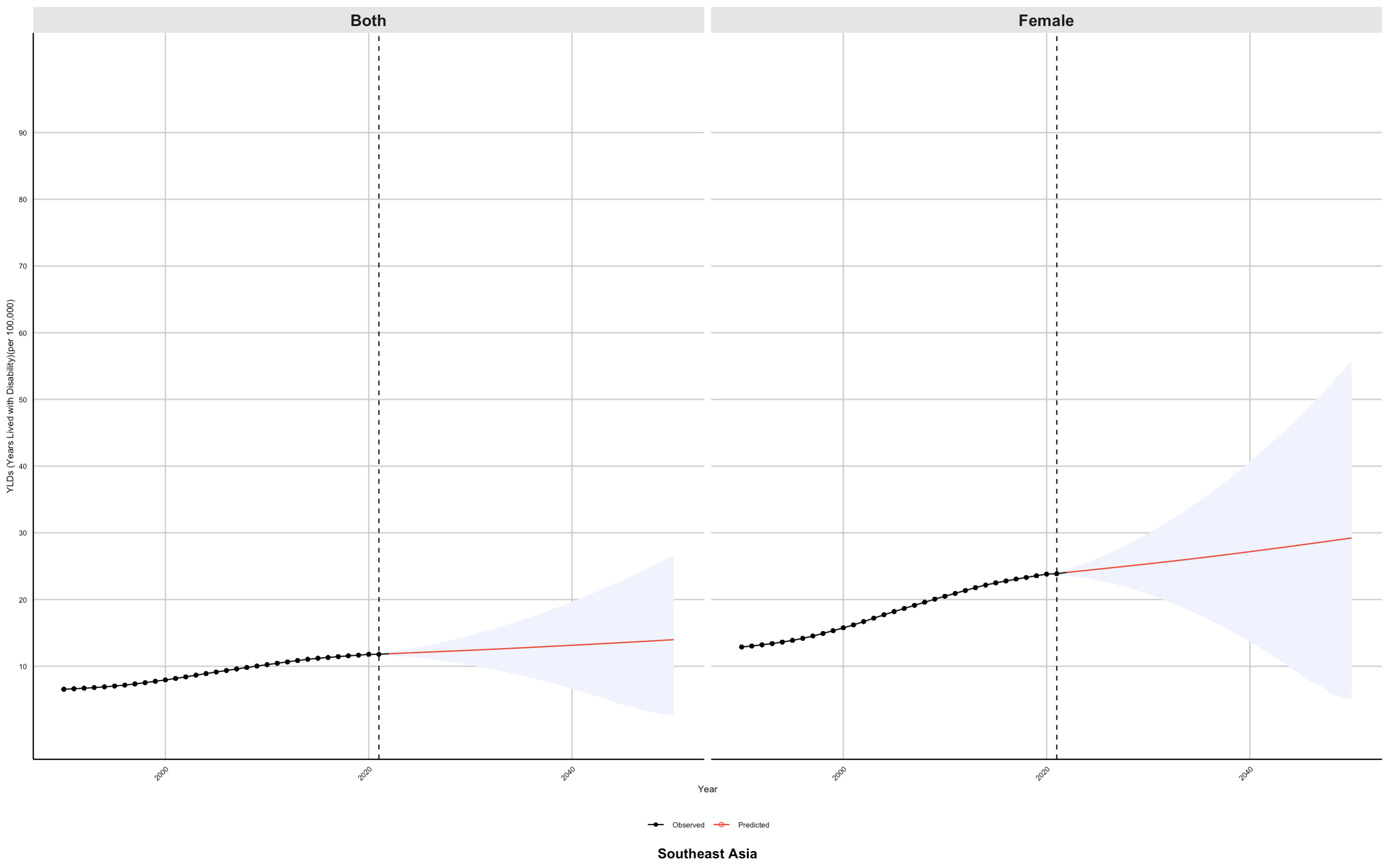

Supplement: Supplementary file 2 [file Supplementaryfile1.zip › Document/Document8-2/S 26/Southeast AsiaBAPC YLDs.png]

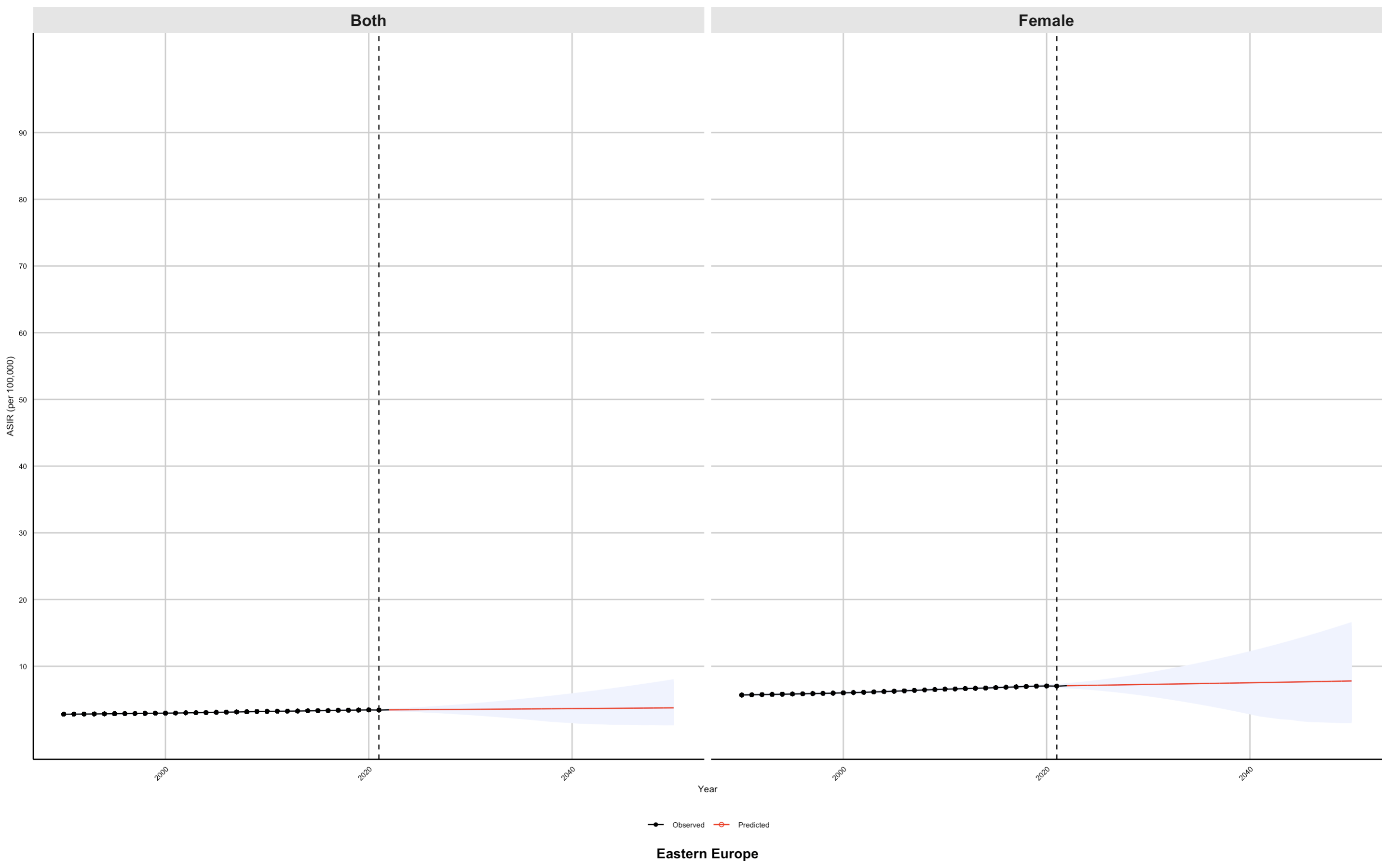

Supplement: Supplementary file 2 [file Supplementaryfile1.zip › Document/Document8-2/S 26/Eastern EuropeBAPC ASIR.png]

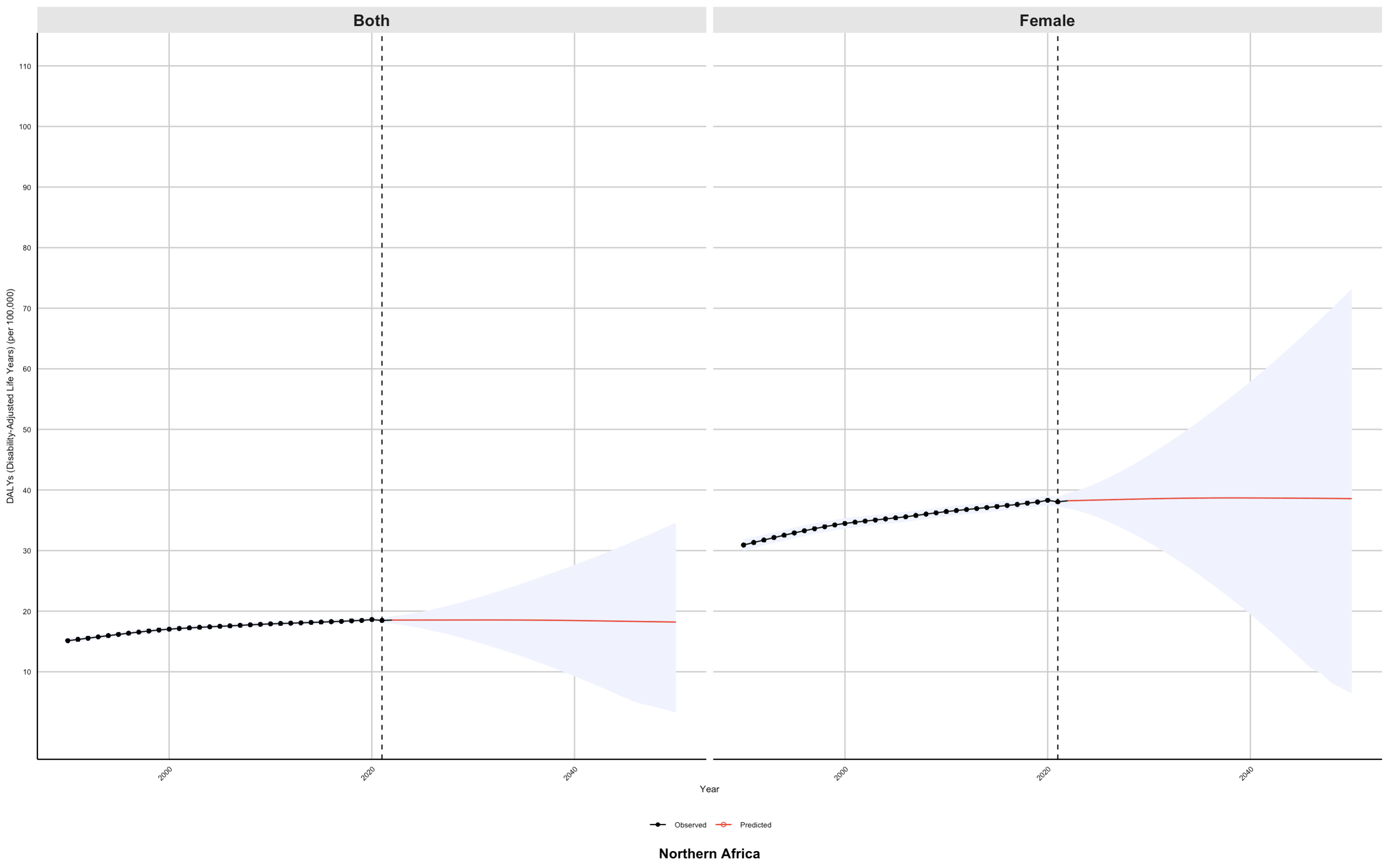

Supplement: Supplementary file 2 [file Supplementaryfile1.zip › Document/Document8-2/S 26/Northern AfricaBAPC DALys.png]

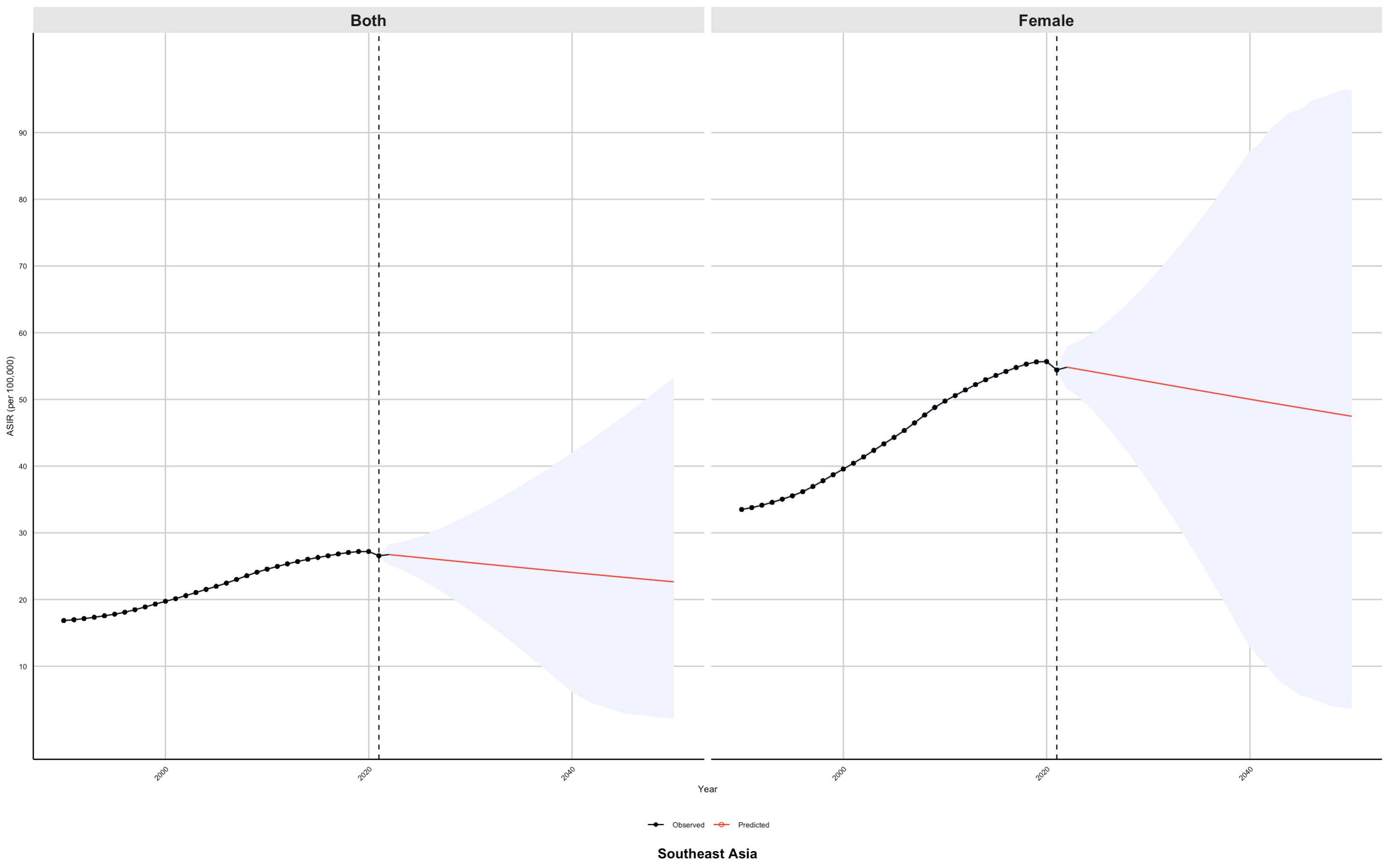

Supplement: Supplementary file 2 [file Supplementaryfile1.zip › Document/Document8-2/S 26/Southeast AsiaBAPC ASIR.png]

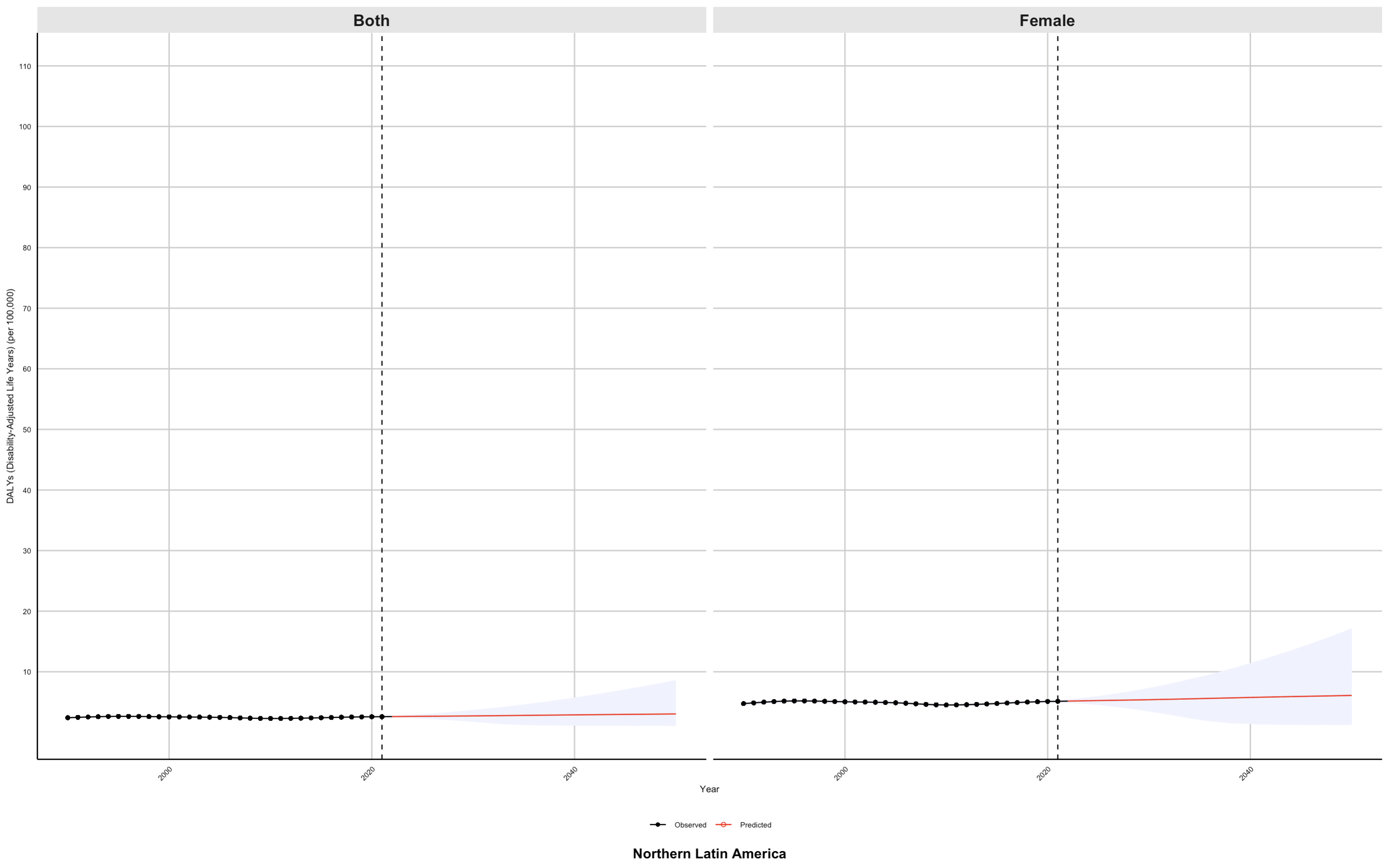

Supplement: Supplementary file 2 [file Supplementaryfile1.zip › Document/Document8-2/S 26/Northern Latin AmericaBAPC DALYs.png]

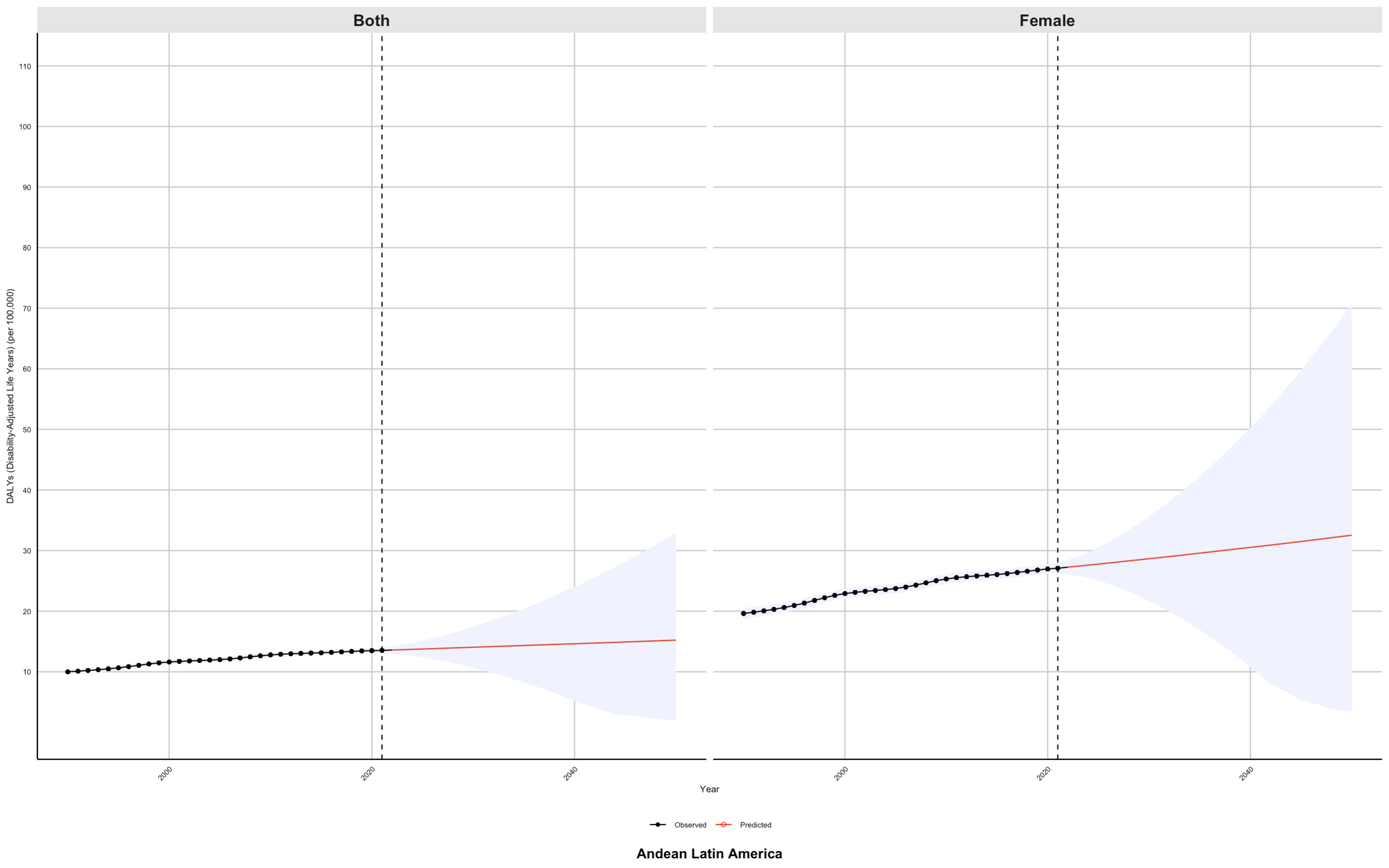

Supplement: Supplementary file 2 [file Supplementaryfile1.zip › Document/Document8-2/S 26/Andean Latin AmericaBAPC DALYS.png]

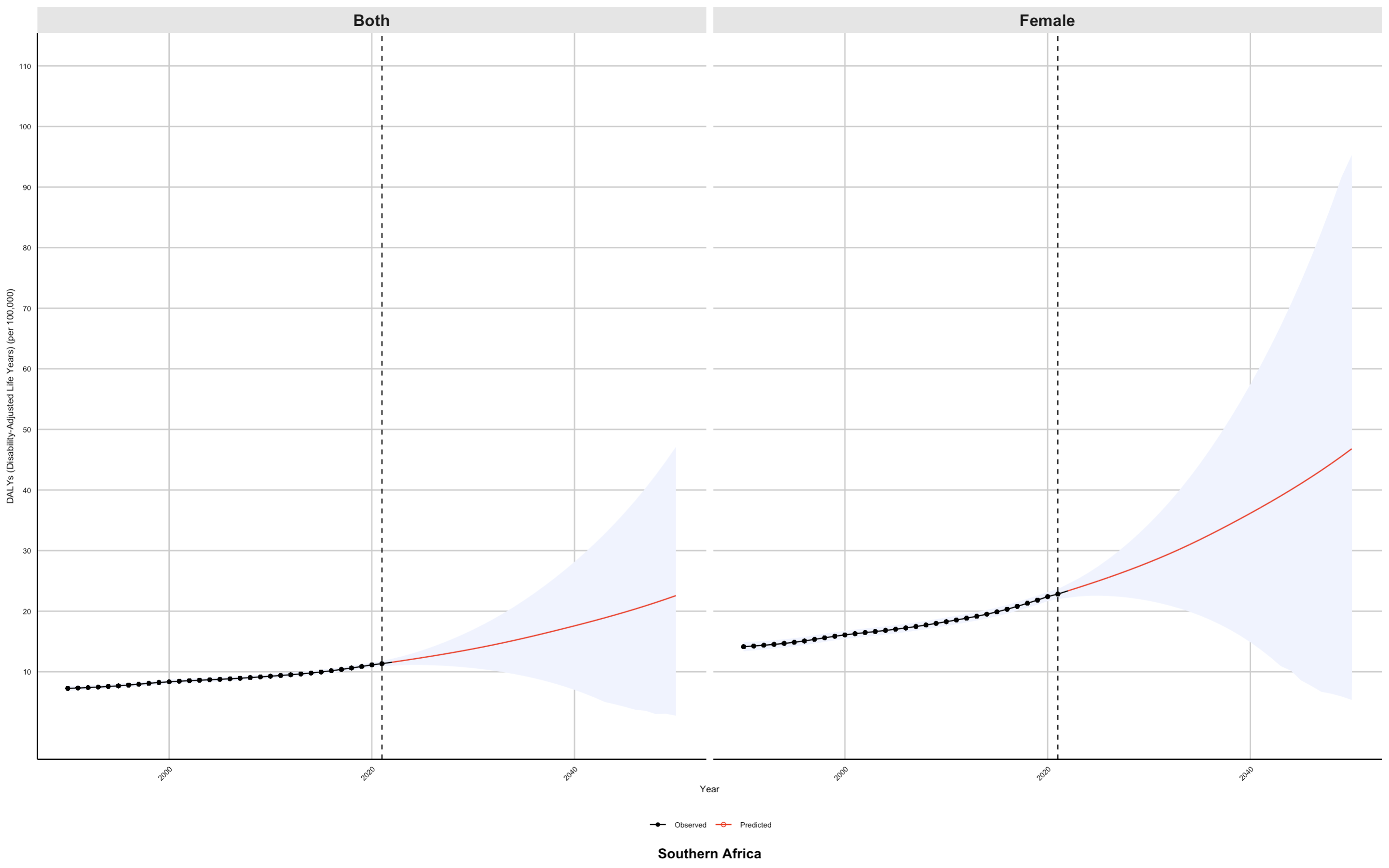

Supplement: Supplementary file 2 [file Supplementaryfile1.zip › Document/Document8-2/S 26/Southern AfricaBAPC DALYs.png]

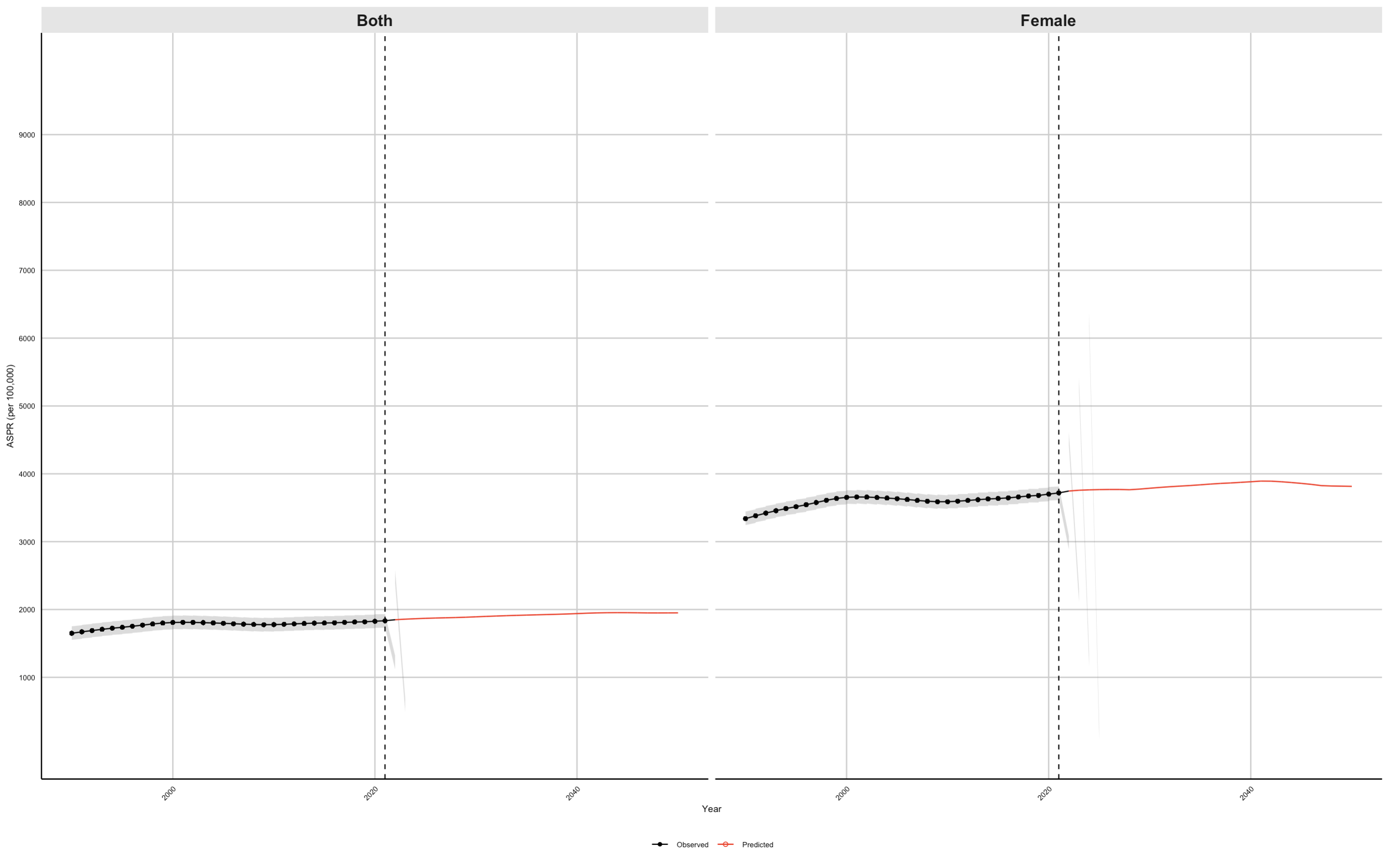

Supplement: Supplementary file 2 [file Supplementaryfile1.zip › Document/Document8-2/S 26/Western EuropeBAPC ASPR.png]

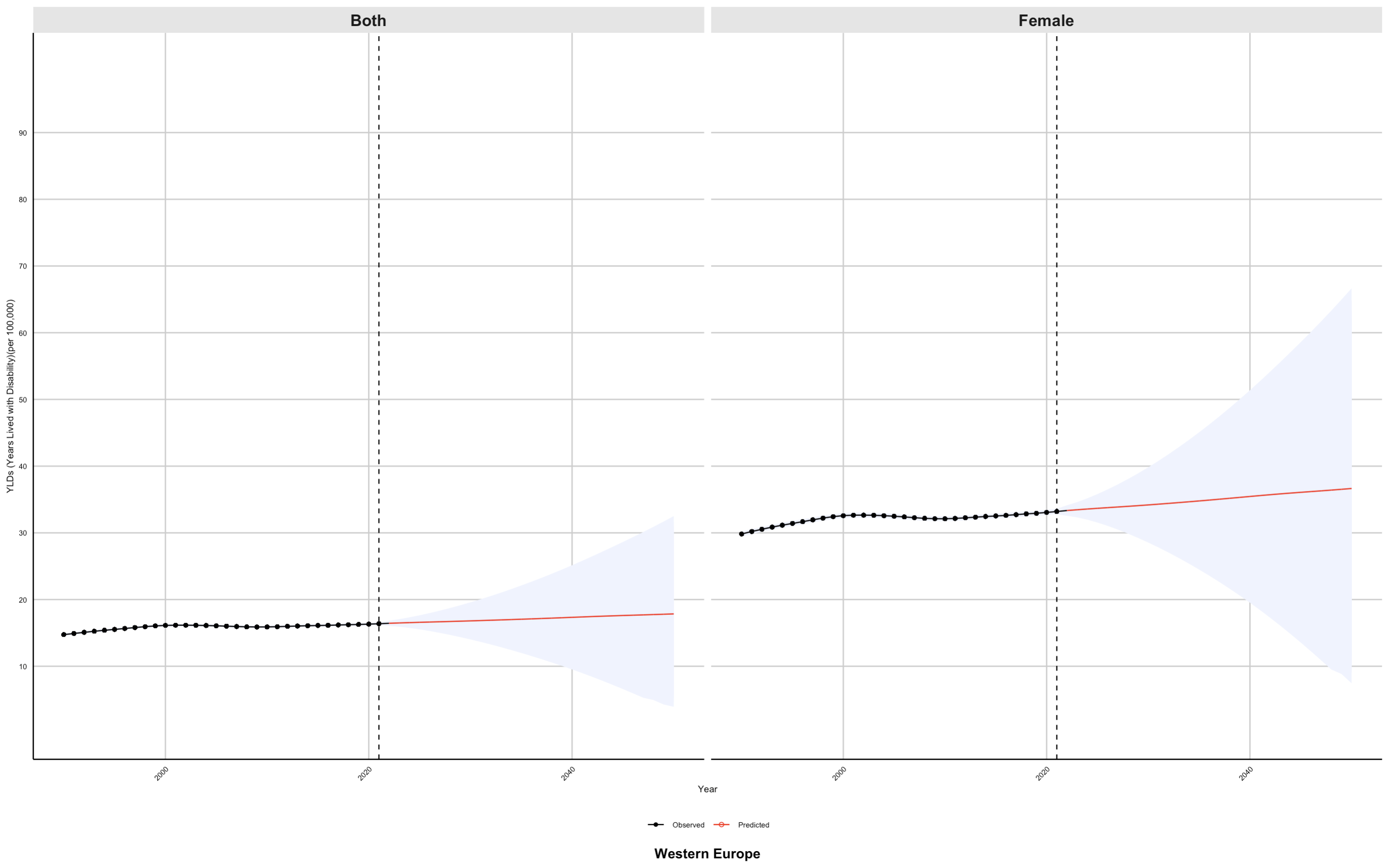

Supplement: Supplementary file 2 [file Supplementaryfile1.zip › Document/Document8-2/S 26/Western EuropeBAPC YLDs.png]

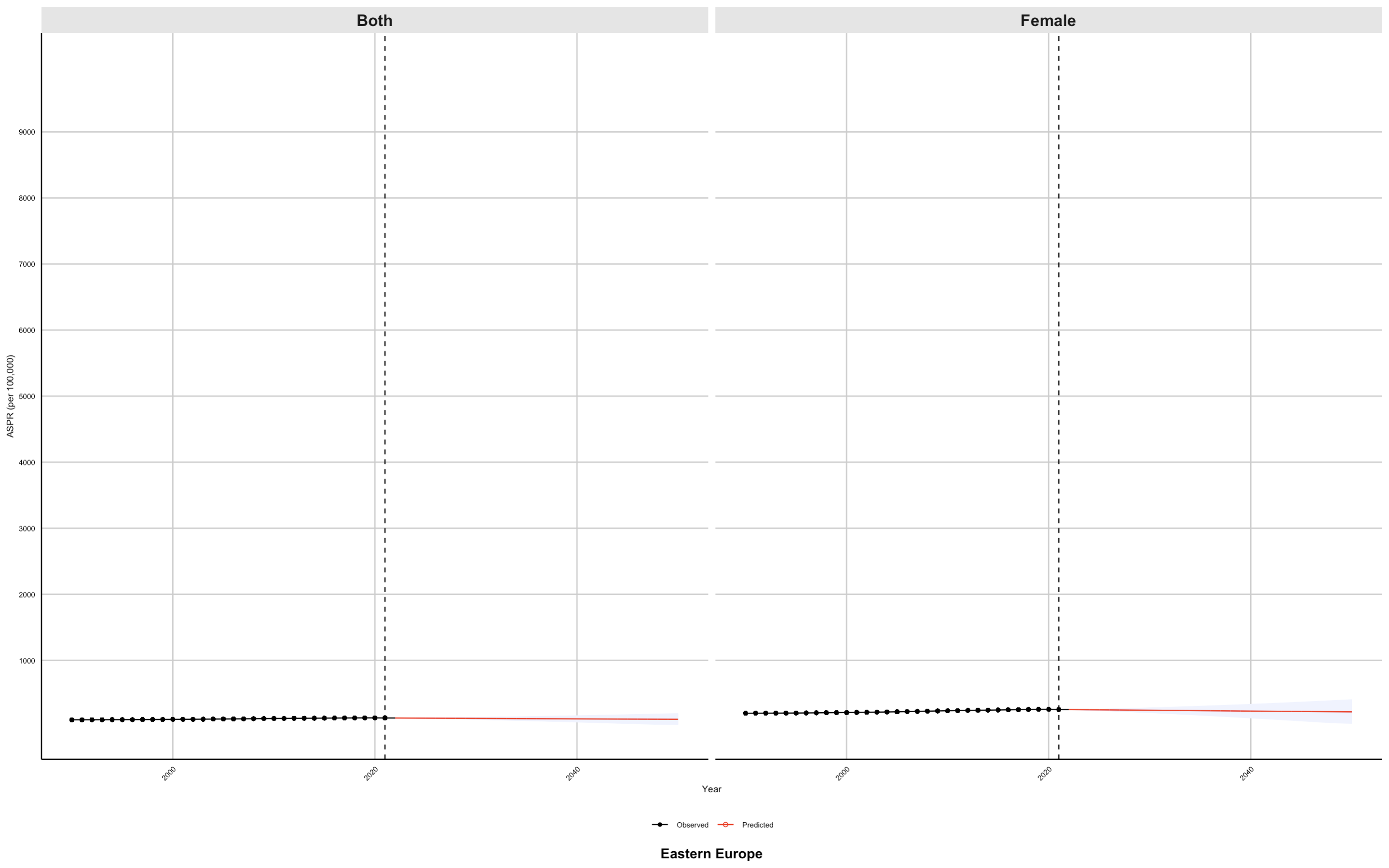

Supplement: Supplementary file 2 [file Supplementaryfile1.zip › Document/Document8-2/S 26/Eastern Europe BPAC ASPR.png]

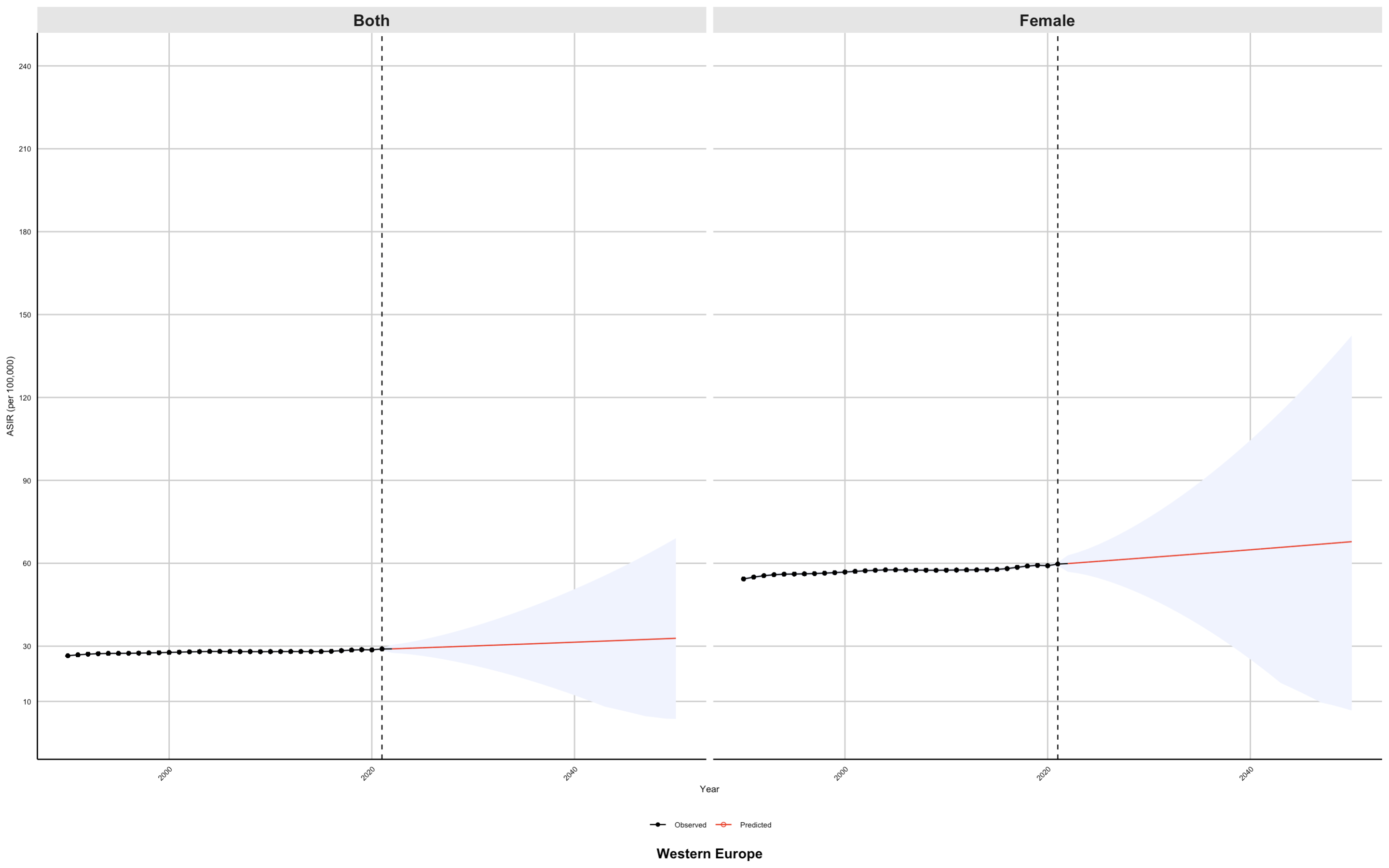

Supplement: Supplementary file 2 [file Supplementaryfile1.zip › Document/Document8-2/S 26/Western EuropeBAPC ASIR.png]

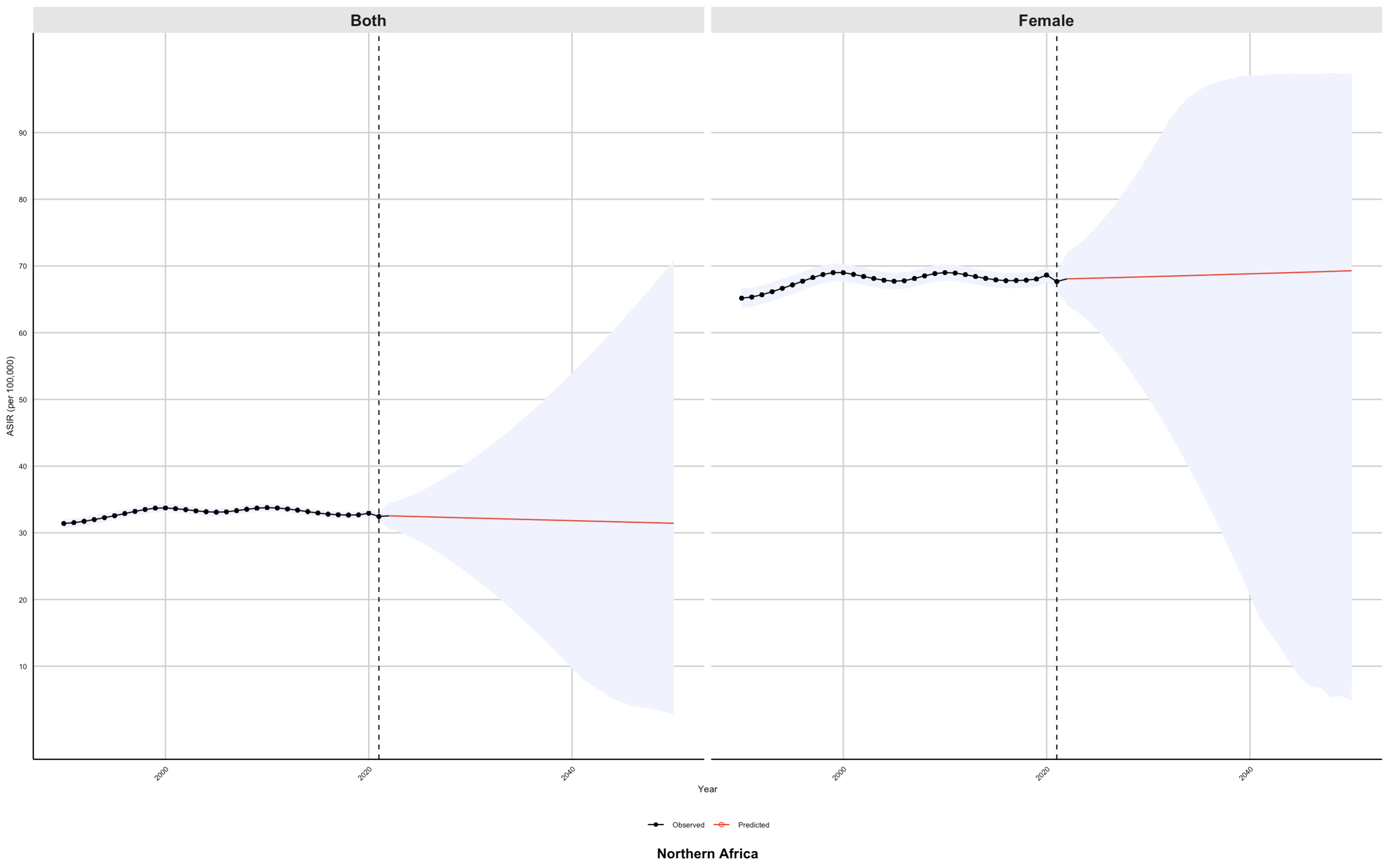

Supplement: Supplementary file 2 [file Supplementaryfile1.zip › Document/Document8-2/S 26/Northern Africa BPAC ASIR.png]

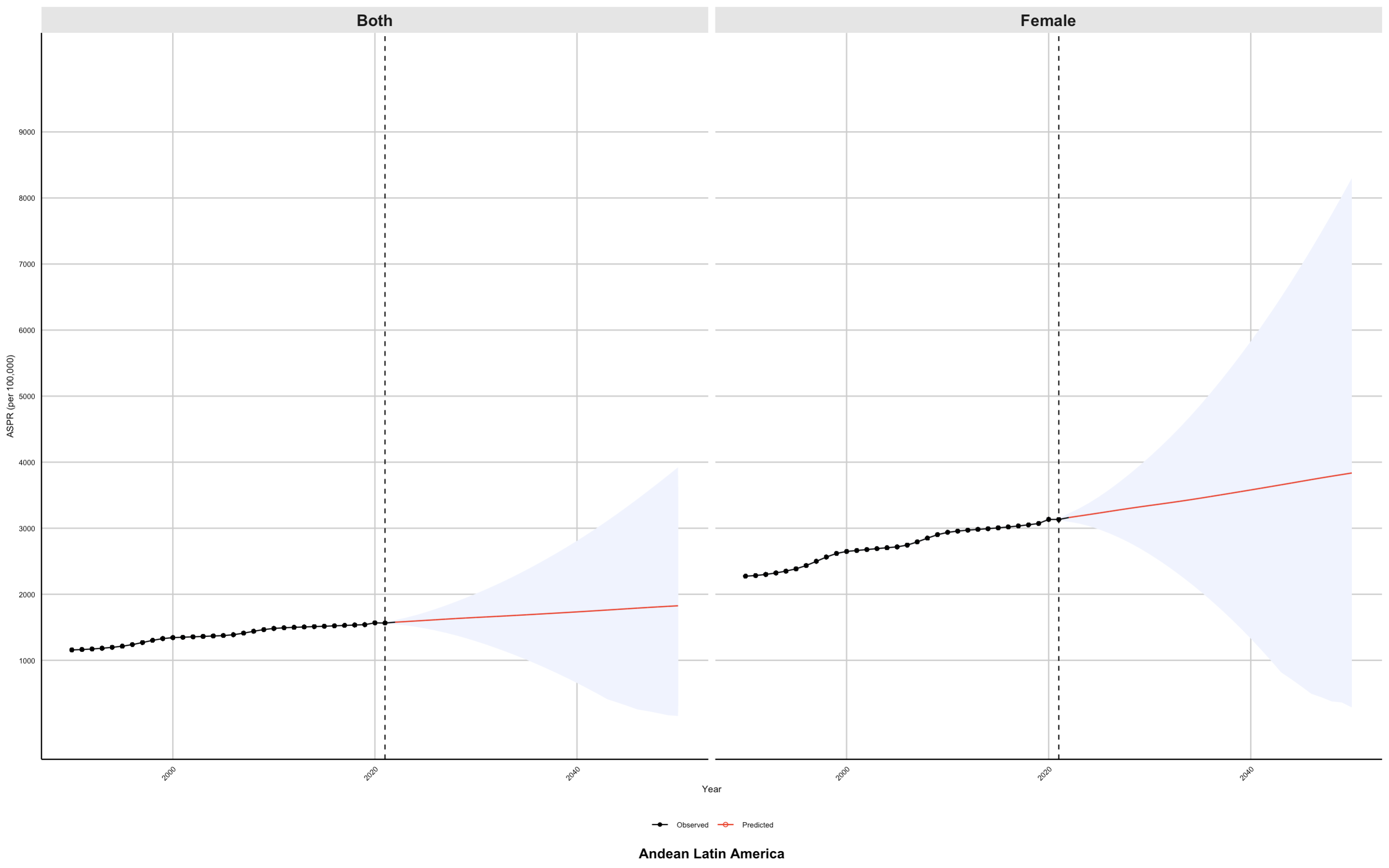

Supplement: Supplementary file 2 [file Supplementaryfile1.zip › Document/Document8-2/S 26/Andean Latin AmericaBAPC ASPR.png]

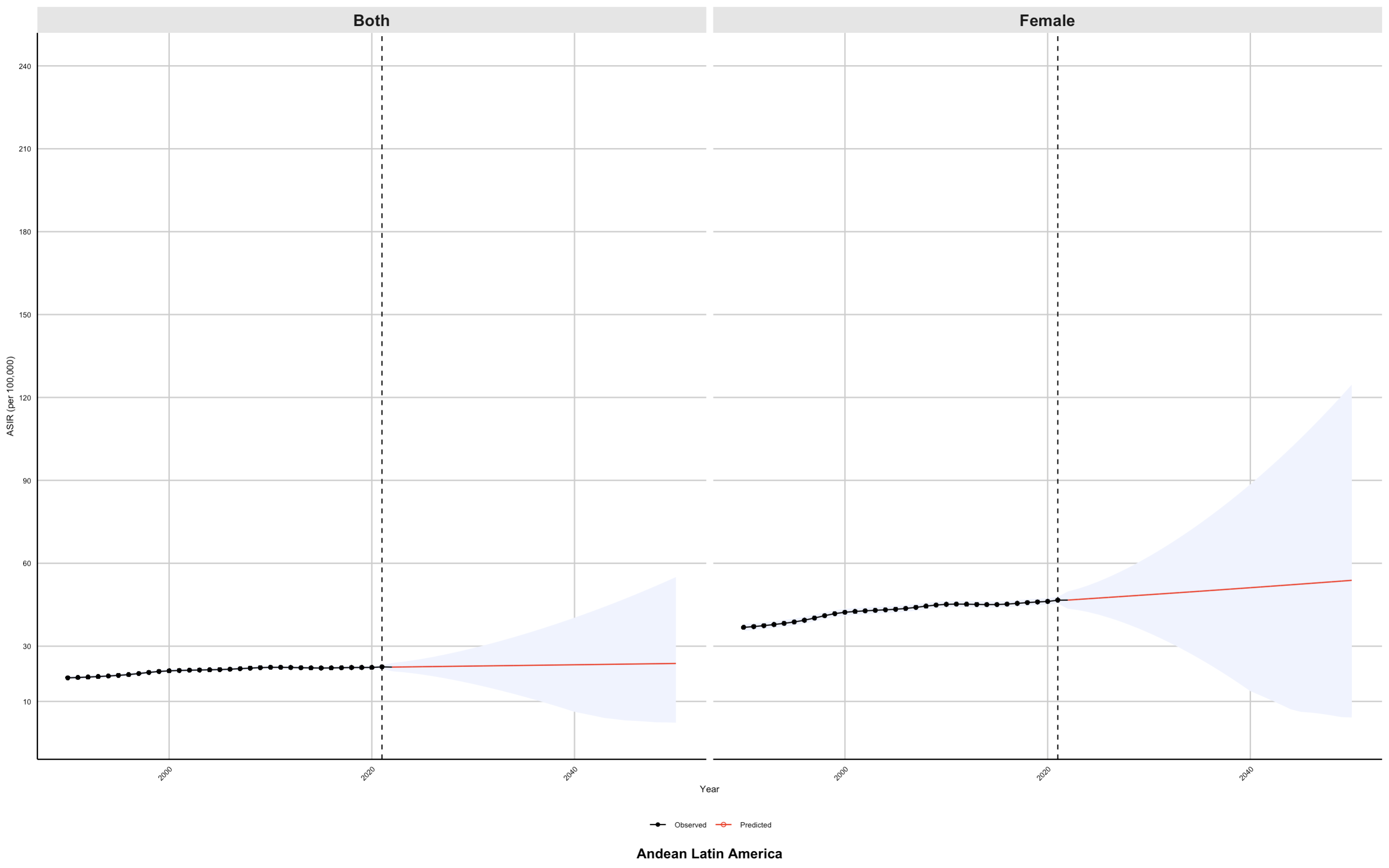

Supplement: Supplementary file 2 [file Supplementaryfile1.zip › Document/Document8-2/S 26/Andean Latin AmericaBAPC ASIR.png]

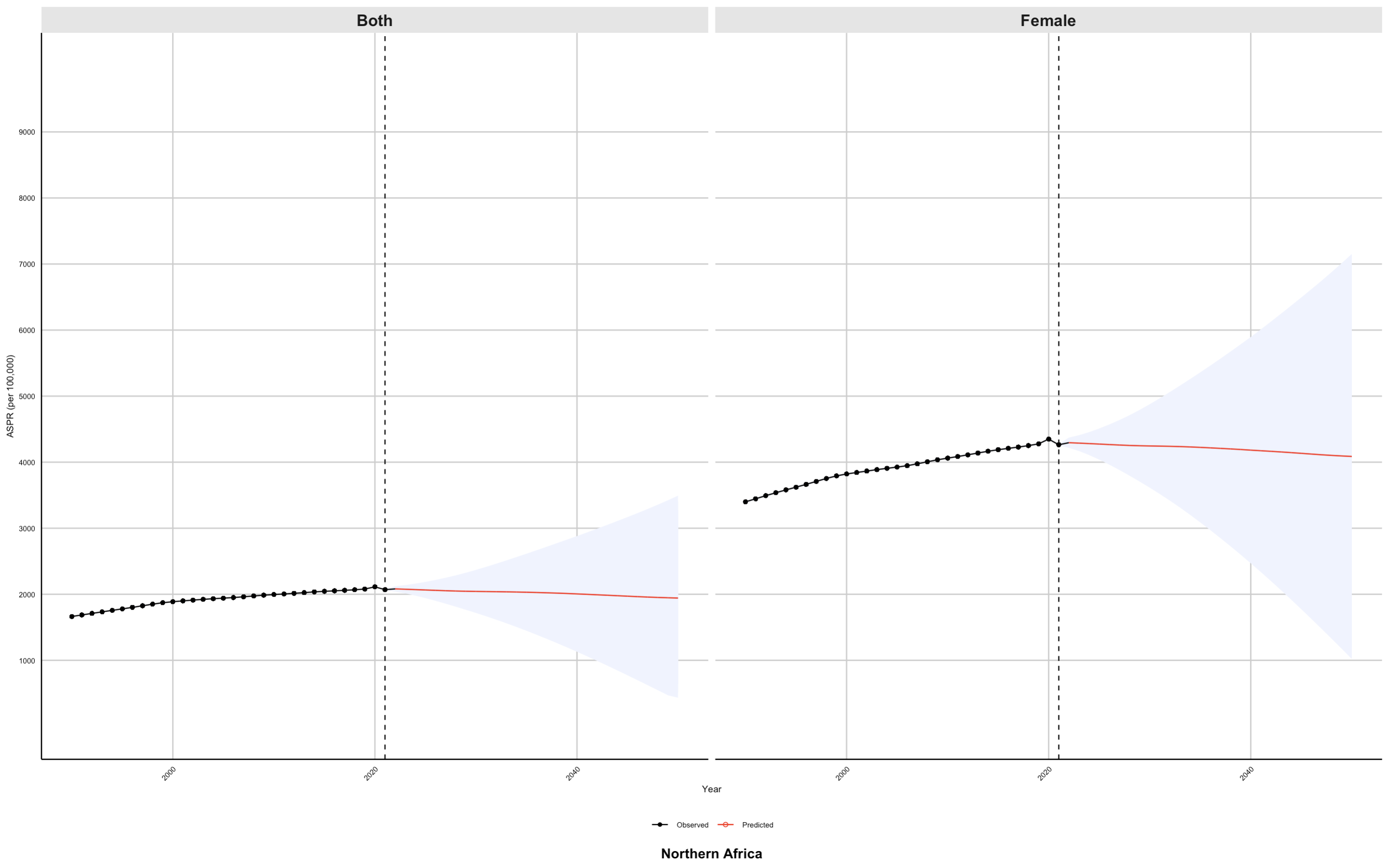

Supplement: Supplementary file 2 [file Supplementaryfile1.zip › Document/Document8-2/S 26/Northern Africa BPAC ASPR.png]

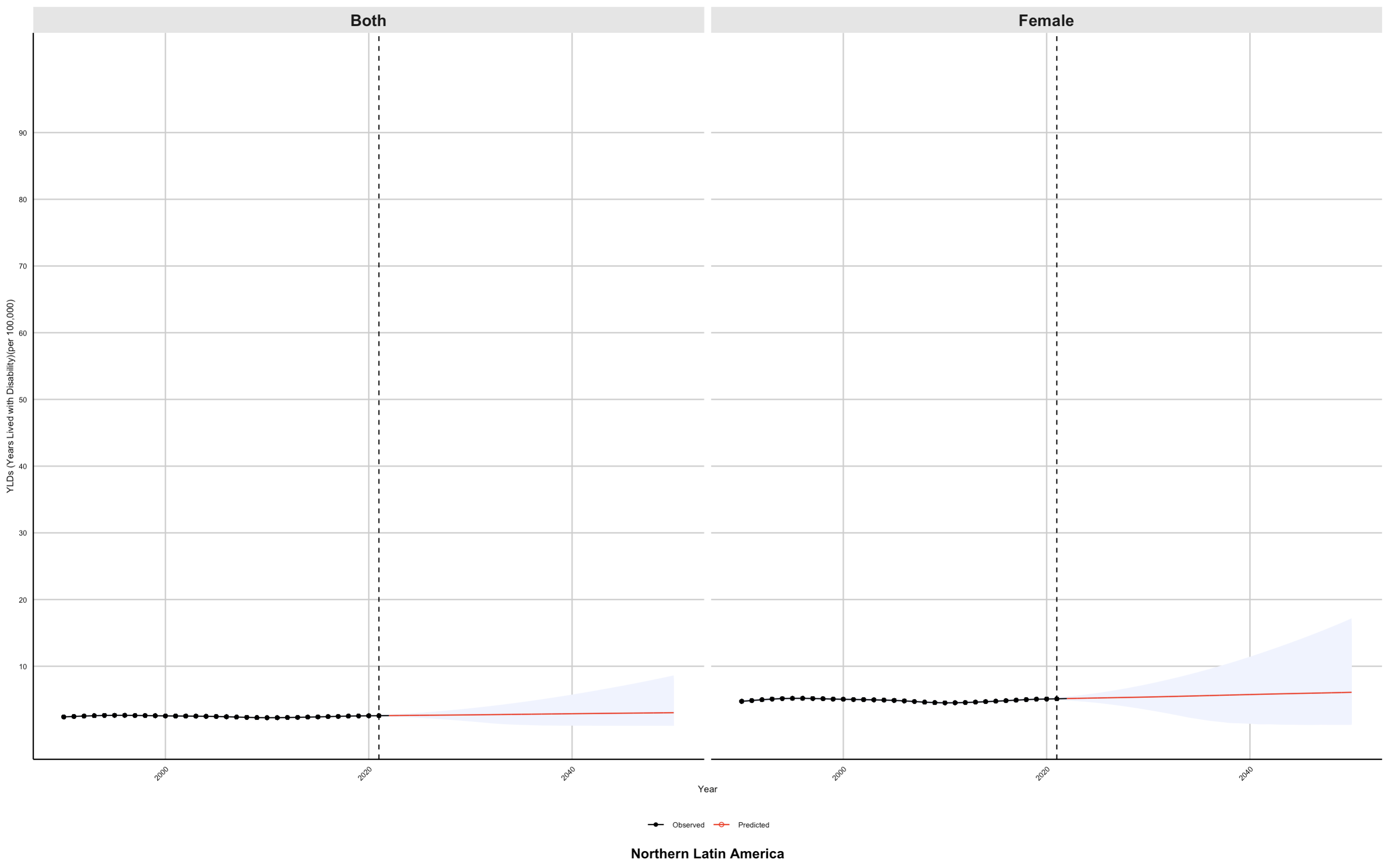

Supplement: Supplementary file 2 [file Supplementaryfile1.zip › Document/Document8-2/S 26/Northern Latin AmericaBAPC YLDs.png]

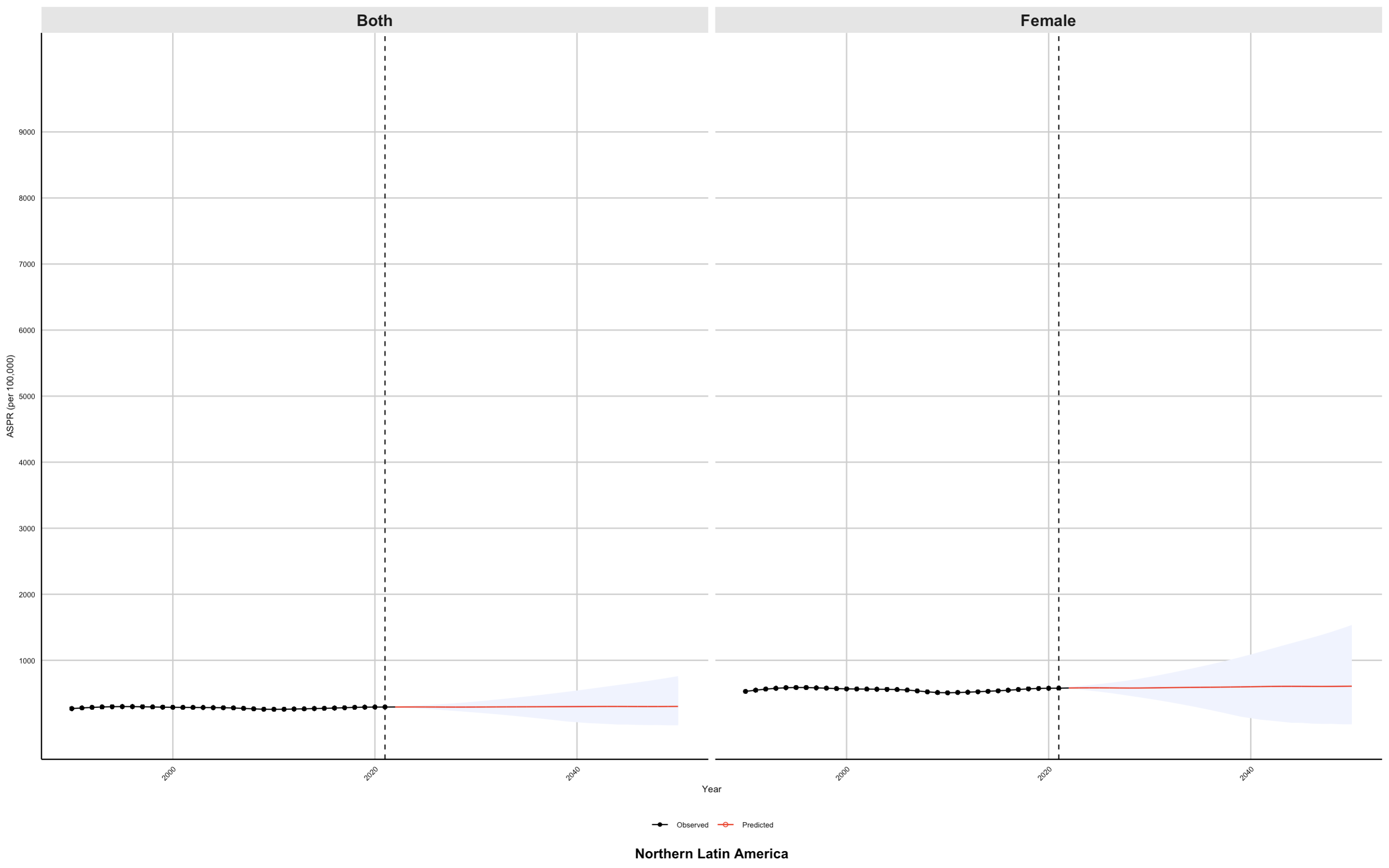

Supplement: Supplementary file 2 [file Supplementaryfile1.zip › Document/Document8-2/S 26/Northern Latin AmericaBAPC ASPR.png]

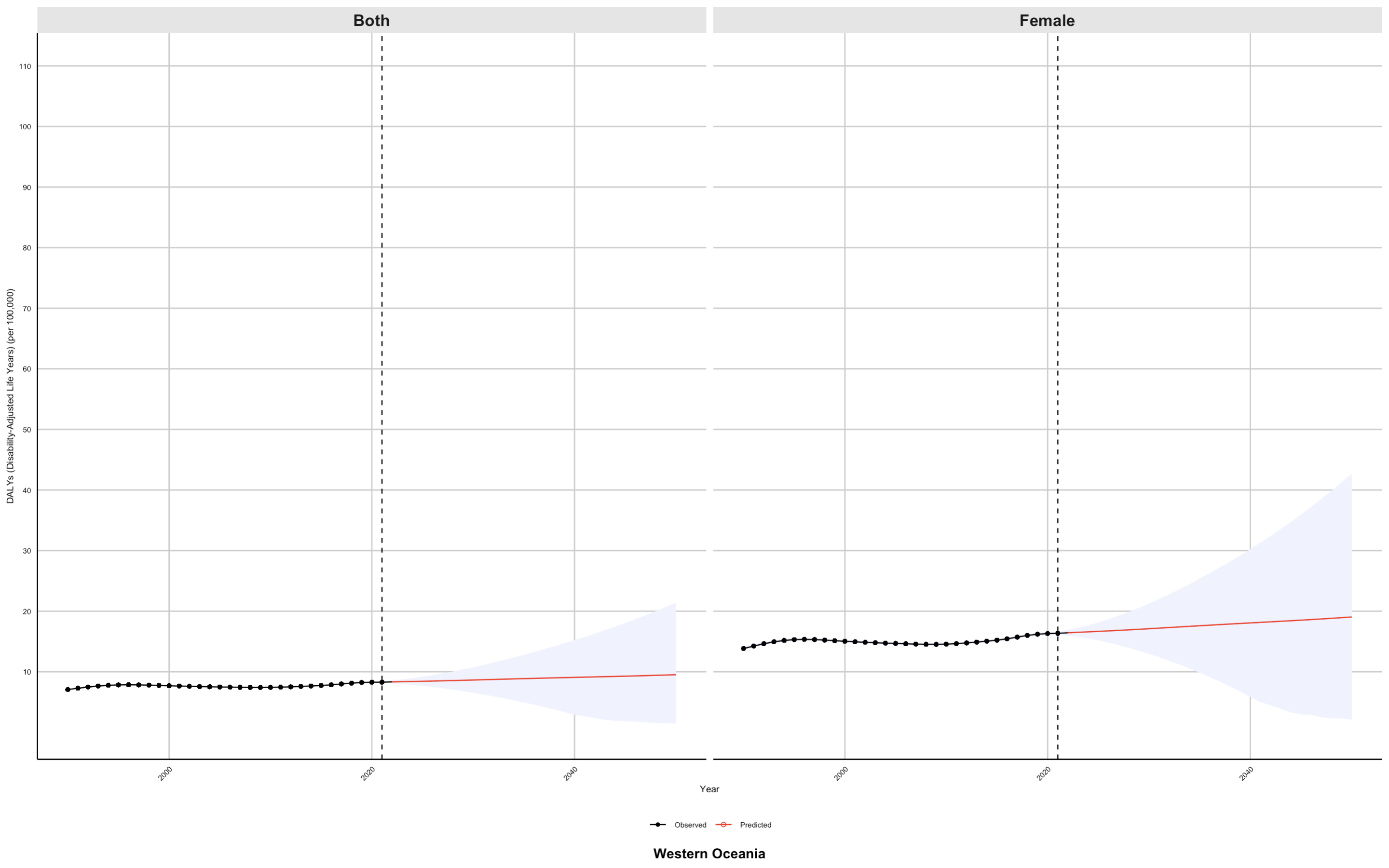

Supplement: Supplementary file 2 [file Supplementaryfile1.zip › Document/Document8-2/S 26/Western Oceania BAPC DALYs.png]

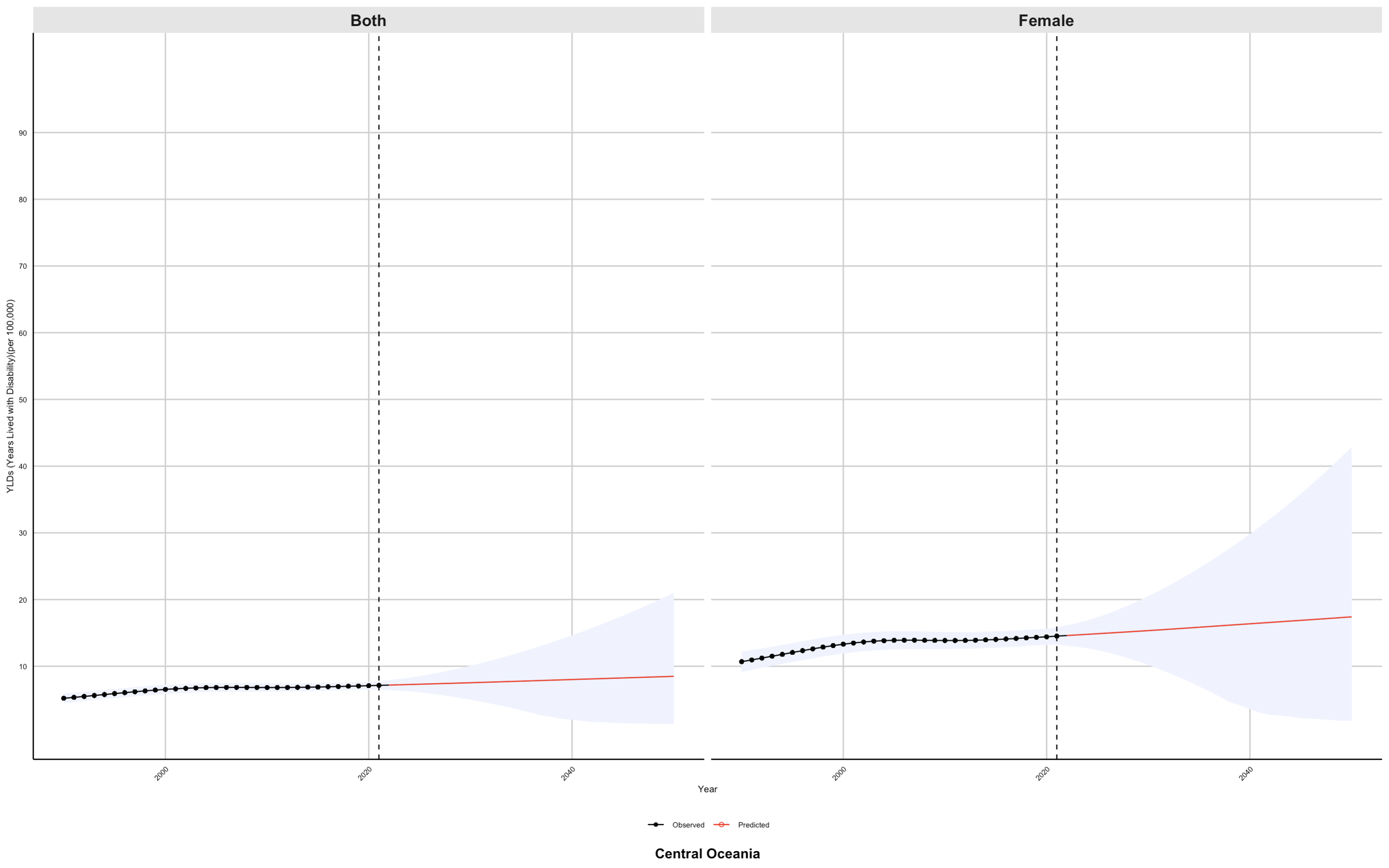

Supplement: Supplementary file 2 [file Supplementaryfile1.zip › Document/Document8-2/S 26/Central OceaniaBAPC YLDs.png]

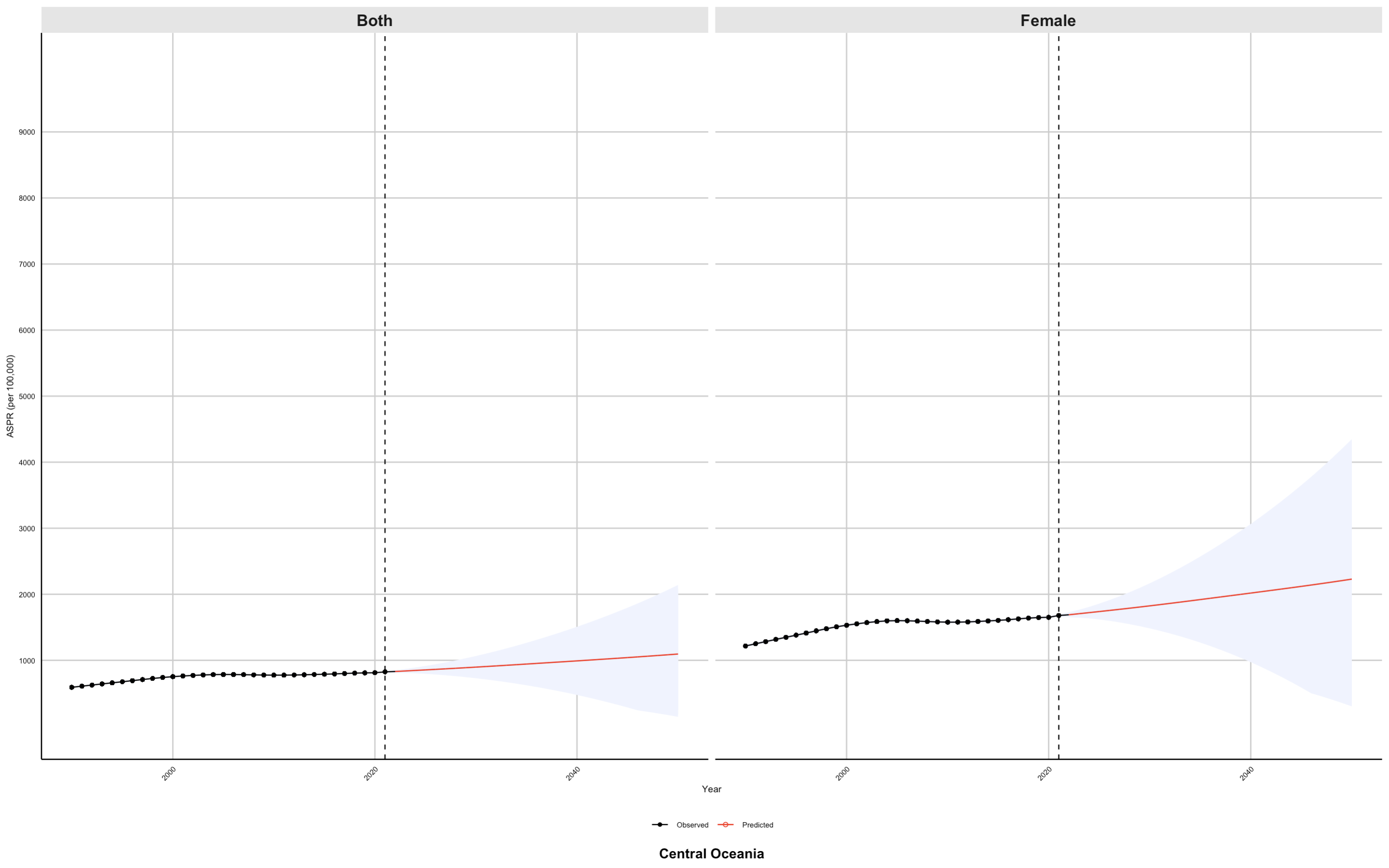

Supplement: Supplementary file 2 [file Supplementaryfile1.zip › Document/Document8-2/S 26/Central OceaniaBAPC ASPR.png]

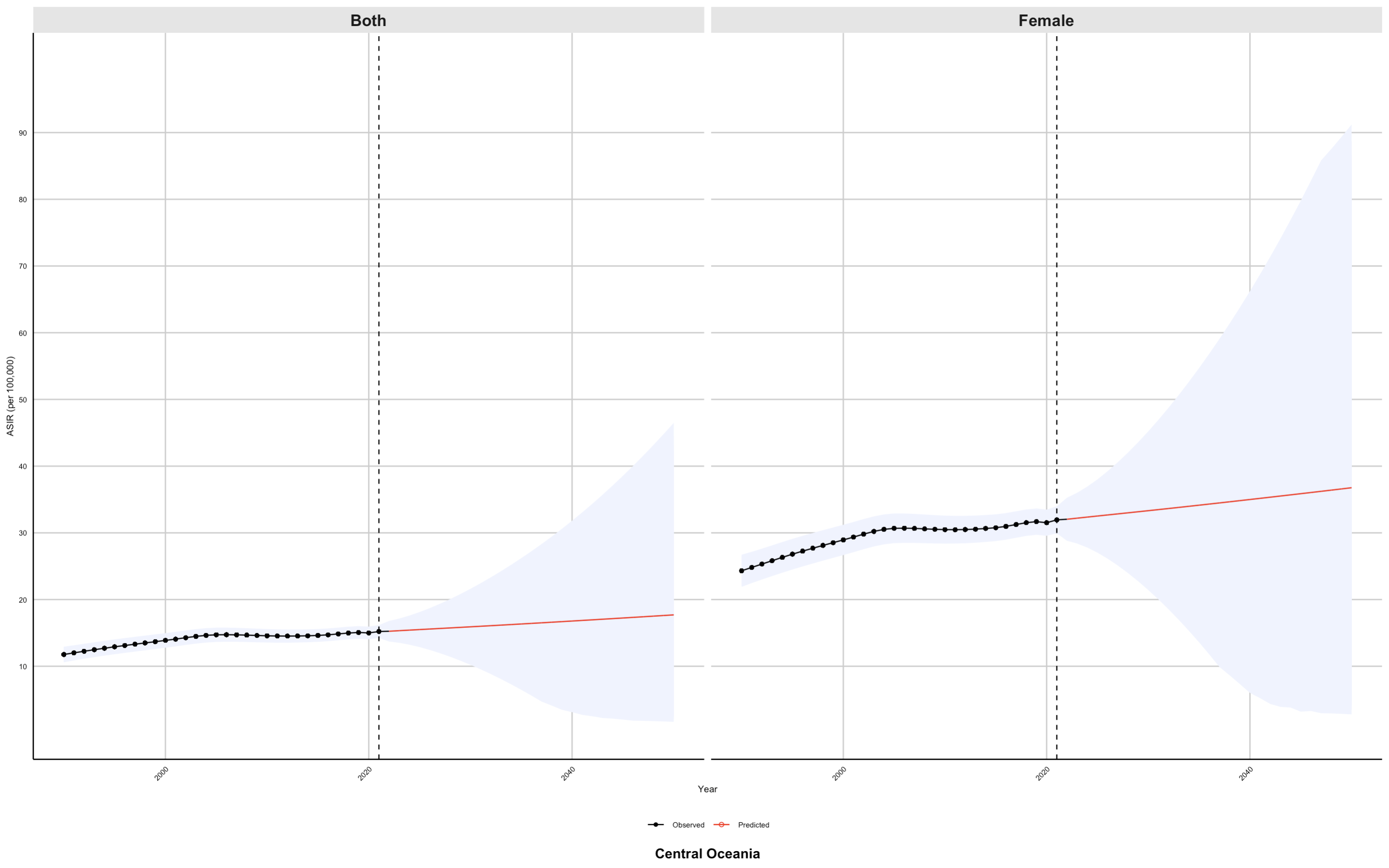

Supplement: Supplementary file 2 [file Supplementaryfile1.zip › Document/Document8-2/S 26/Central OceaniaBAPC ASIR.png]

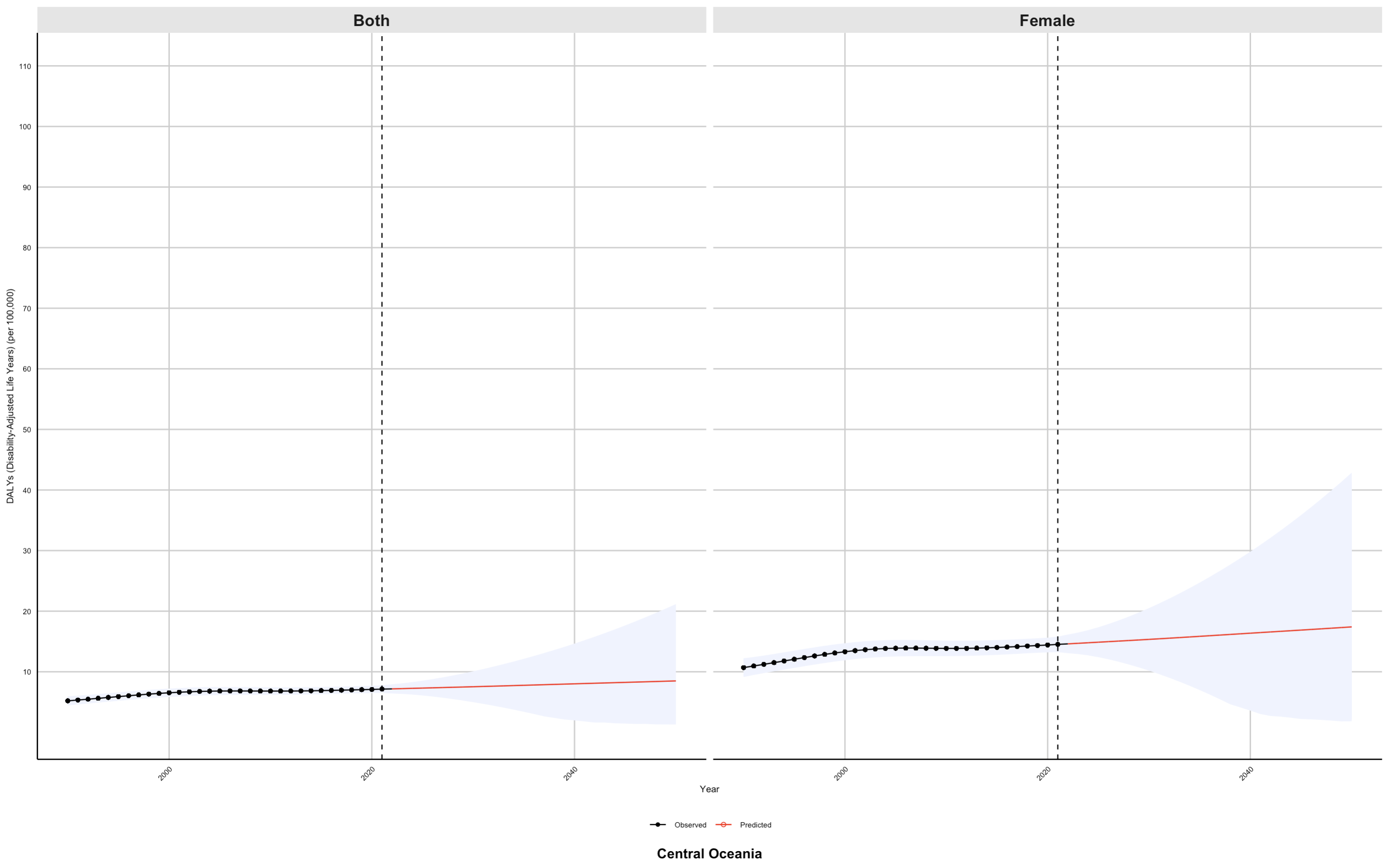

Supplement: Supplementary file 2 [file Supplementaryfile1.zip › Document/Document8-2/S 26/Central OceaniaBAPC DALYS.png]

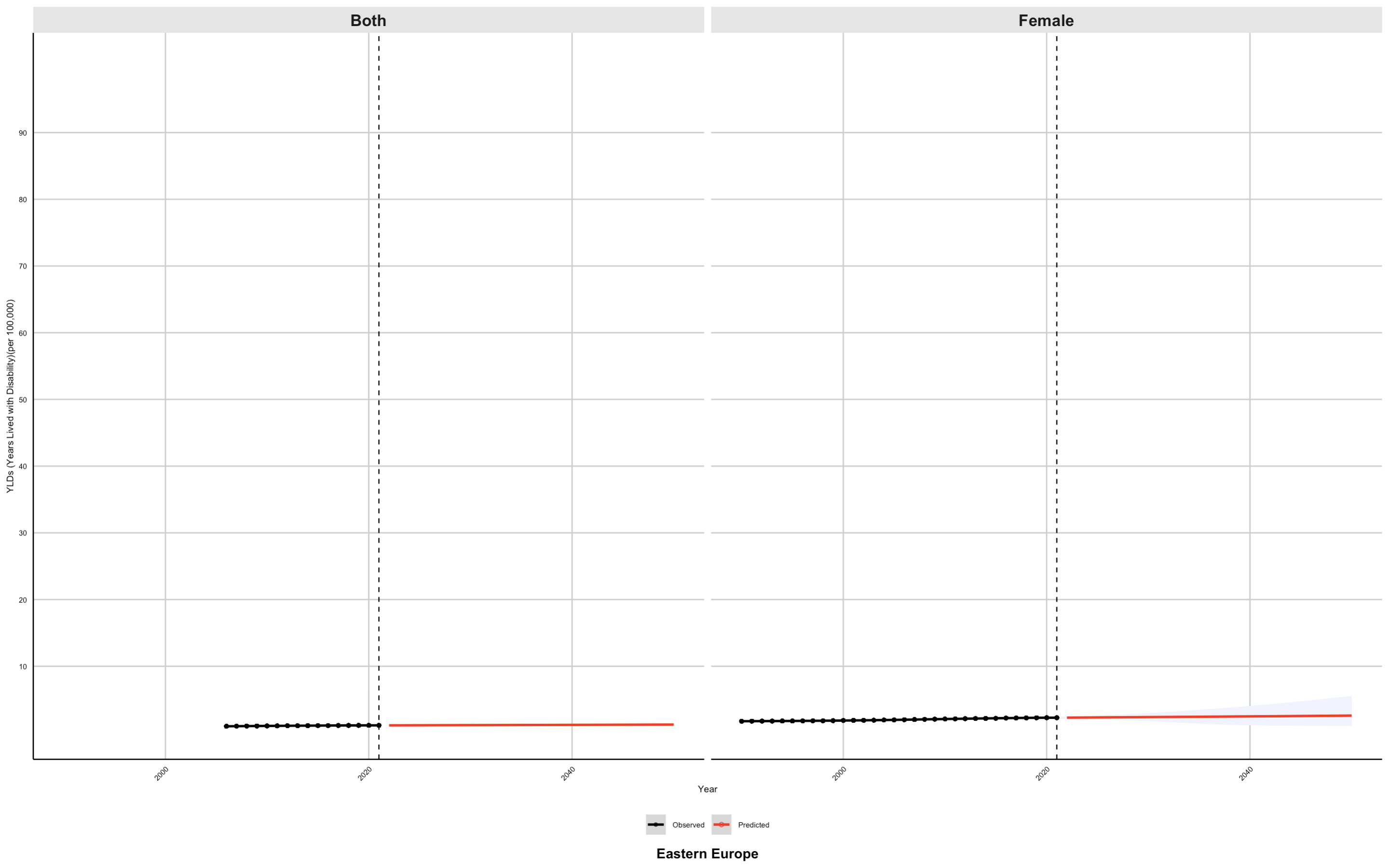

Supplement: Supplementary file 2 [file Supplementaryfile1.zip › Document/Document8-2/S 26/Eastern Europe BAPCYLDs.png]
